# Thiazol-4-one derivatives from the reaction of monosubstituted thioureas with maleimides: structures and factors determining the selectivity and tautomeric equilibrium in solution

**DOI:** 10.3762/bjoc.12.251

**Published:** 2016-11-29

**Authors:** Alena S Pankova, Pavel R Golubev, Alexander F Khlebnikov, Alexander Yu Ivanov, Mikhail A Kuznetsov

**Affiliations:** 1 Institute of Chemistry, Saint Petersburg State University, Universitetsky pr. 26, Saint Petersburg, 198504, Russia; 2 Centre for Magnetic Resonance, Saint Petersburg State University, Universitetsky pr. 26, Saint Petersburg, 198504, Russia

**Keywords:** maleimides, regioselectivity, tautomerism, thiazolidinones, thioureas

## Abstract

2-(Alkyl(aryl)amino)thiazol-4(5*H*)-ones can regioselectively be prepared from monoalkyl(aryl)thioureas and maleimides. In solution, the former heterocycles exist in a tautomeric equilibrium with 2-(alkyl(aryl)imino)thiazolidin-4-ones and the substituent on the exocyclic nitrogen atom governs the ratio of these tautomers. Isomers with the alkyl group in the endocyclic position can be obtained from *N*-methyl(ethyl)thioureas. 2D NMR spectroscopy and DFT calculations rationalize experimental results.

## Introduction

**Scheme 1:**
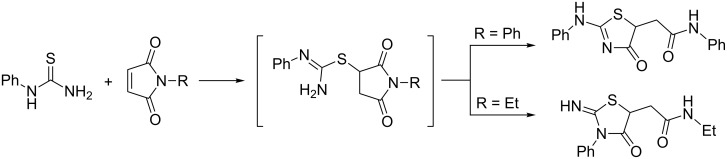
The regioselectivities of the reaction between monosubstituted thioureas and maleimides according to [9].

The thiazolidine core is one of the privileged scaffolds in a variety of pharmaceuticals with exclusively broad range of biological activities [1–7]. 2-Aminothiazolidin-4-ones can be easily prepared treating thiourea derivatives with various dielectrophiles [8]. In particular, the addition of monoalkyl(aryl)thioureas to N-substituted maleimides provides the corresponding thiazolidinylacetamides. First described by Marrian in 1949 [9] and later developed further by Augustin and co-workers [10–11], a plausible mechanism for this transformation was postulated. It includes a nucleophilic attack of the thiourea sulfur atom on the C=C bond of the maleimide followed by a proton transfer and nucleophilic attack of the thiourea nitrogen atom on one of the two carbonyl groups; the latter step is considered rate determining (Scheme 1). It was reported that the substituent on the maleimide nitrogen atom, particularly a group in the *para*-position of the phenyl ring in *N*-arylmaleimides, affects the reactivity of the maleimide toward nucleophilic addition [10]. Strange as it may seem, *N*-phenylthiourea reacts with *N*-phenylmaleimide providing thiazolidine with the exocyclic position of the phenyl group, whereas an isomer with the phenyl group on the endocyclic nitrogen atom was obtained from the same thiourea and *N*-ethylmaleimide (Scheme 1) [9]. This behavior has not been explained and there is lack of more recent information on the regioselectivity of this reaction. On the other hand, the addition of monosubstituted thioureas to maleic anhydride [12–16] or its open-chain derivatives [17–18] leads to thiazolidines with the parent thiourea’s substituent in the exocyclic position.

Another unresolved issue is the nature of the dynamic process observed in solutions of 2-aminothiazolidines. This phenomenon was first detected by UV spectroscopy [19] and believed to be caused by a proton transfer between nitrogen atoms (amino–imino tautomerism), however, contradictory information on the equilibrium ratio of tautomers exist [15,18,20–23]. (*E*/*Z*)-Isomerization of the exocyclic C=N bond of the single imino form was suggested as the cause for the dynamic effect as well [24–25]. In addition, this process complicates the interpretation of NMR spectra of thiazolidines and is a likely source of misinterpretations. For example, the reported NMR data of 2-arylaminothiazol-4-one derivatives is given as one set of signals, while the actual spectra clearly displayed signals of two distinct forms [17].

In order to remove these ambiguities, and in line with our previous work [26], we set about to thoroughly investigate the regioselectivity of the reaction between monosubstituted thioureas and maleimides and the tautomerism of the products. The results of this investigation are reported herein.

## Results and Discussion

**Scheme 2:**
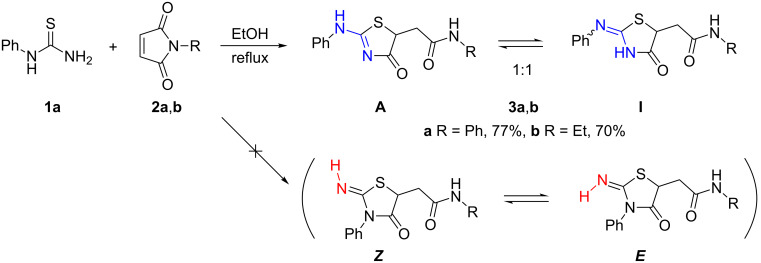
Reaction of *N*-phenylthiourea (**1a**) with maleimides **2a**,**b** (conceivable products are given in parentheses).

First, we repeated some experiments reported by Marrian [9], namely, the reactions of *N*-phenylthiourea (**1a**) with *N*-phenyl- and *N*-ethylmaleimides **2a**,**b**. The experimental protocol [9] required reflux temperature for reaction with **2a** and room temperature for **2b**. We obtained thiazolidines **3a**,**b** as single products in both cases according to the ^1^H NMR spectra of the reaction mixtures (Scheme 2). Performing the latter reaction under reflux conditions provided the same result.

The very similar ^1^H NMR spectra of both compounds had obvious features of dynamic processes in solution: two broad downfield signals in a ratio ≈ 1:1 (δ 11.16 and 11.74 ppm for **3a**, 11.11 and 11.69 ppm for **3b**, DMSO-*d*_6_) and two sets of insufficiently resolved signals of other protons. The presence of two forms follows from the ^13^C NMR spectra as well. Taking into account the differences in nucleophilicity between the unsubstituted and phenyl-substituted nitrogen atoms of thiourea **1a**, the phenyl group in compounds **3a**,**b** is more likely to be found in the exocyclic position. A possible explanation is the presence of two tautomeric forms (the amino form **A** and the imino form **I**) (Scheme 2). At the same time, the structure with endocyclic phenyl group was proposed by Marrian [9] and it could not be ruled out. In this case two interconverting *E/Z*-isomers at the exocyclic C=N bond (given in parentheses on Scheme 2) could be involved.

Therefore we have thoroughly analyzed the spectroscopic data of thiazolidine **3a** from 2D NMR experiments (NOESY, ^13^C,^1^H and ^15^N,^1^H-HSQC and HMBC) and made a full assignment of all signals. A large difference in the chemical shift (0.8 ppm) was found for the positions of the fragment originating from the thiourea’s phenyl *ortho*-protons of the two forms. However, signals of the corresponding carbon atoms did not differ significantly. The chemical shifts of the thiazolidine carbonyl C^4^ atom signals of the two forms differ by 12 ppm. The corresponding ^15^N–^1^H correlation spectra allowed determining the signal of the exocyclic amide nitrogen atom (δ ≈ 133 ppm); only one of the two thiourea nitrogen atoms could be detected in the product. A direct correlation was found between the signals of the nitrogen atom at δ ≈ 127 ppm and the proton at δ 11.23 ppm. This nitrogen atom also correlates with the phenyl *ortho*-protons (δ 7.81 ppm) meaning that both, the proton and the phenyl ring, are attached to the same nitrogen atom, which is only possible for the **A** form among the four structures shown in Scheme 2. These results allow us to exclude the structure with the endocyclic position of parent thiourea’s phenyl group and the *E*/*Z*-isomerism from consideration. However, no proof for the **I** form of **3a** was obtained because no correlations for the proton at δ 11.83 ppm were observed, possibly because of the partial or full localization of this proton on the nearest oxygen atom.

The NMR spectroscopic correlations described above were found for compound **3b** as well. According to the ^15^N,^1^H-HMBC spectrum, the signal of the exocyclic NH proton of the **A** form (δ_H_ 11.00 ppm, δ_N_ ≈ 128 ppm) correlates with the nitrogen atom at δ ≈ 235 ppm, which is obviously the C=N atom of this form. No correlations were found for the proton signal at δ 11.68 ppm of **3b**-**I** as mentioned for **3a-I**.

**Figure 1:**
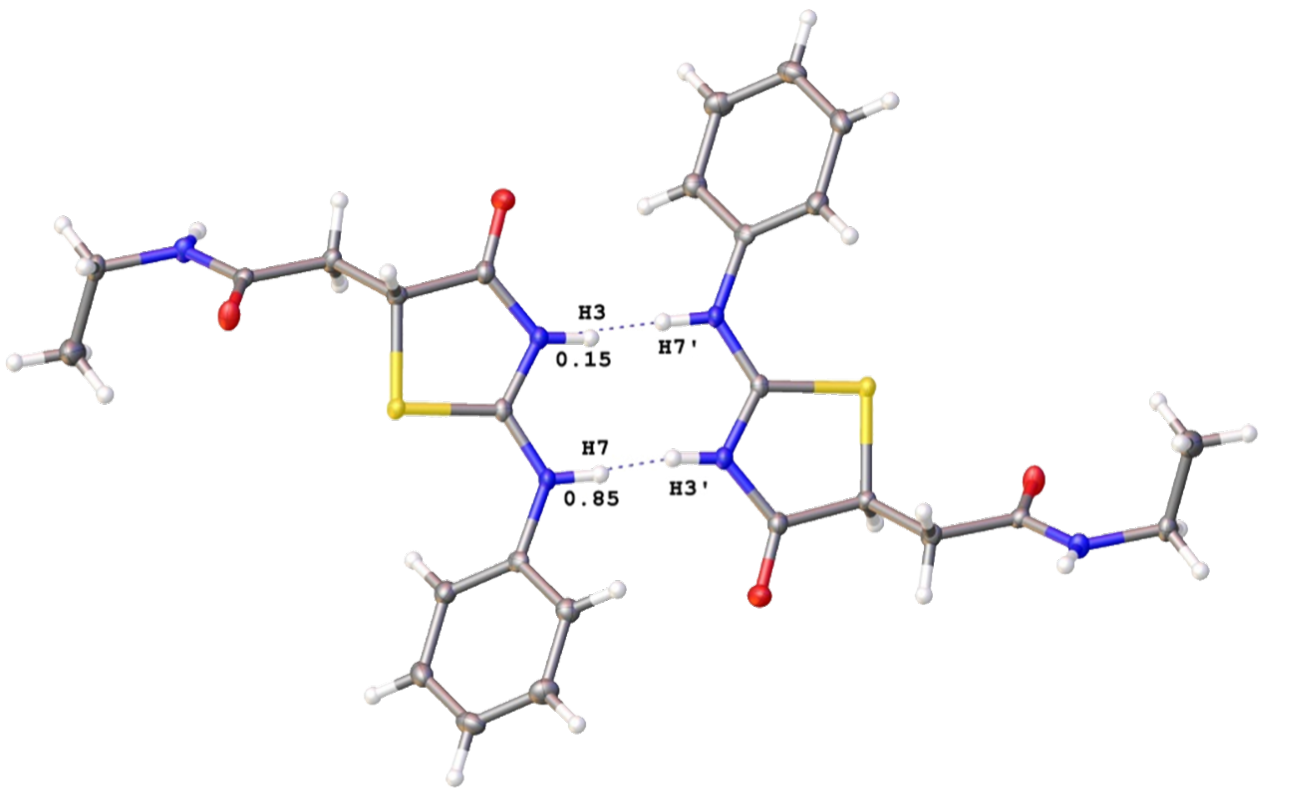
OLEX2 representation of the crystal structure of thiazolidine **3b**. Thermal ellipsoids are given at the 50% probability level.

However, X-ray analysis indicated this proton in the crystal structure of **3b** (Figure 1) [27] and there are two partially occupied positions of the “jumping” hydrogen atom in the structure of **3b** (see Supporting Information File 1). This confirms the two tautomeric **A** and **I** forms of thiazolidine **3b** rather than *E*/*Z*-isomers with the endocyclic phenyl group. While in solution the ratio of tautomers is 1:1, in the solid state the **A** form prevails.

The signal of the *ortho*-protons of the phenyl group at the nitrogen atom of **3a**,**b-I** is located 0.7–0.8 ppm upfield than those next to the NH group of the **A** form, although the corresponding *ortho*-carbon signals are very close (Δδ ≈ 1.1–1.2 ppm). The observation was also made for the related thiazolidines **3c–g**, see below.

We then varied substrates **1** and **2** to examine the influence of substitution on the reaction regioselectivity and on the ratio of the tautomeric products (Table 1). In all cases thiazolidines **3** were obtained as single products with tautomeric mixtures of the **A** and **I** forms in solution. As expected, the electronic character of the substituent at the phenyl ring of maleimide **2** (compare **2a**, Scheme 2, and **2c**,**d**, Table 1) as well as the size of an alkyl group (compare **3b**, Scheme 2, and **3e**, Table 1) did not affect the product structure or the tautomeric ratio.

**Table 1:** Reaction of monosubstituted thioureas **1a**–**f** with maleimides **2a**–**e**.

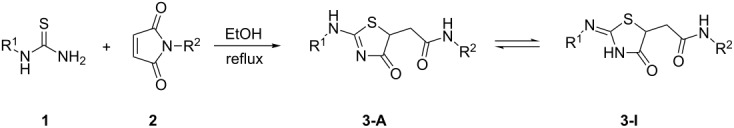						

Thiourea	R^1^	Maleimide	R^2^	Thiazolidine	Isolated yield, %	Ratio **A**:**I**

**1a**	Ph	**2c**	4-EtOC_6_H_4_	**3c**	66	1:1
**1a**	Ph	**2d**	4-O_2_NC_6_H_4_	**3d**	89	1:1
**1a**	Ph	**2e**	*c*-Hex	**3e**	56	1:1
**1b**	4-MeOC_6_H_4_	**2a**	Ph	**3f**	65	1:1
**1c**	4-O_2_NC_6_H_4_	**2a**	Ph	**3g**	73	1:1.8
**1d**	*c*-Hex	**2a**	Ph	**3h**	80	4:1
**1d**	*c*-Hex	**2b**	Et	**3i**	74	4:1
**1e**	Et	**2a**	Ph	**3j**	66	4:1
**1f**	Me	**2a**	Ph	**3k**	50	4:1

**Figure 2:**
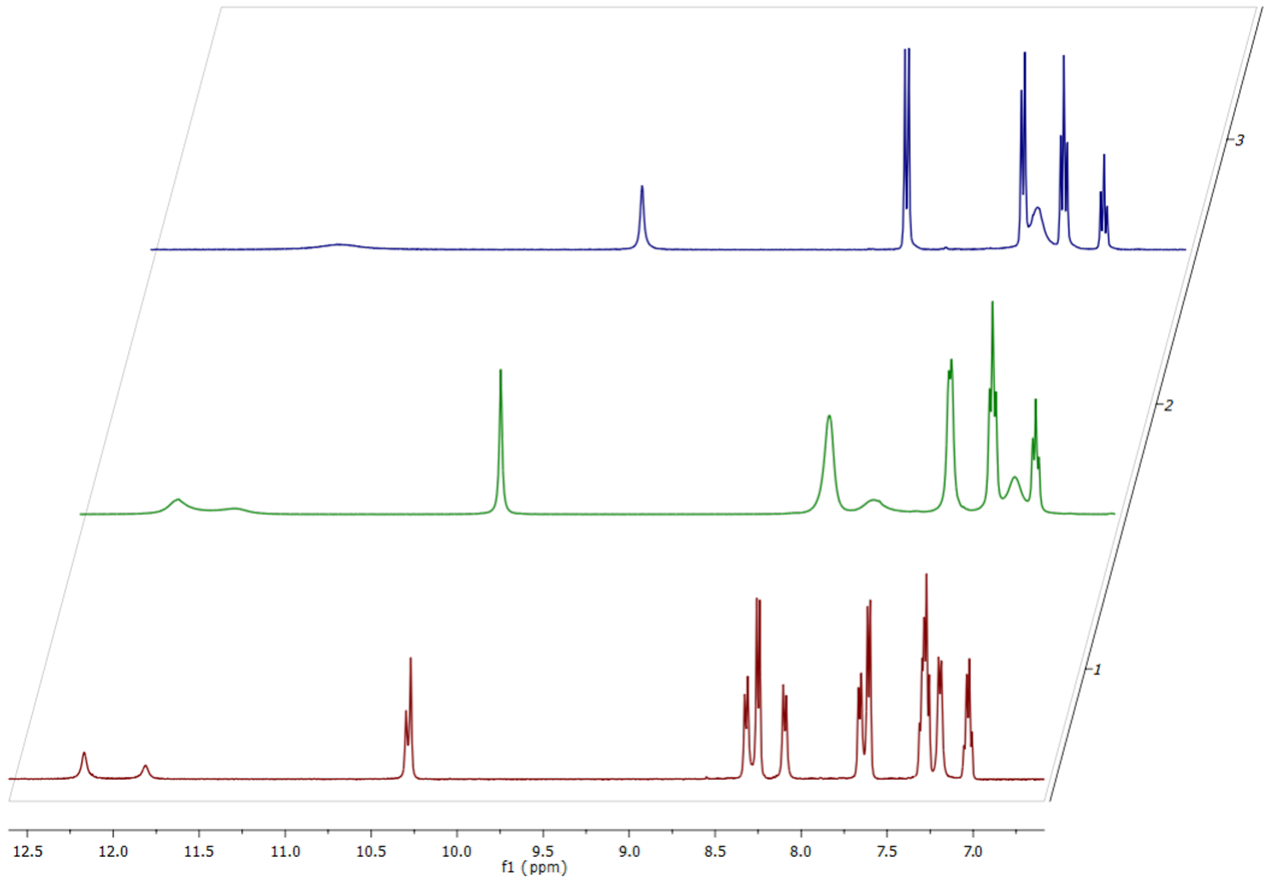
Fragments of the ^1^H NMR spectra of thiazolidine **3g** at −20 °C (1), 23 °C (2) and 120 °C (3). Spectrum (1) was recorded at 500 MHz, spectra (2) and (3) at 400 MHz, respectively.

A substituent in the thiourea is more likely to exert influence on the reaction regioselectivity and/or position of the tautomeric equilibrium. Nevertheless, the same regioselectivity was observed in the reactions of arylthioureas **1b**,**c** with *N*-phenylmaleimide (**2a**) as for phenylthiourea (**1a**) (Table 1). Thioureas **1b**,**c** appeared to be less active than **1a** because a 1.5-fold excess of maleimide **2a** was necessary to reach full conversion. Although the **A**:**I** ratio for thiazolidine **3f** was found to be 1:1 as for all other phenyl-substituted analogues **3a–e**, the nitro-substituted thiazolidine **3g** features an **A**:**I** ratio of ca. 1:1.8. The tautomerization process of thiazolidine **3g** in solution led to a very strong broadening of its signals in the ^1^H NMR spectrum at 23 °C (Figure 2). Decreasing the temperature to −20 °C slowed down the proton transfer and the two sets of signals could be clearly seen and were fully assigned to the two forms of **3g** as it had been done earlier for **3a**. On the other hand, heating the solution of thiazolidine **3g** up to 120 °C led to coalescence. At this temperature even the signals of the *ortho*-protons of the 4-nitrophenyl ring (Δδ = 0.9 ppm at −20 °C) coalesced into one (Figure 2).

The deviation of the tautomeric ratio for **3g** from the ones observed for thiazolidines **3a–f** seems to be caused by a weakening of the conjugation in the fragment NH–C=N–C=O realized in **A** form due to the strong electron-withdrawing effect of the 4-nitrophenyl group at the terminal NH group.

Introducing an alkyl group in thioureas **1d–f** had no effect on the regioselectivity of the addition to maleimide **2** forming thiazolidines **3h–k** (Table 1). Although alkyl groups generally increase the nucleophilicity of the adjacent nitrogen atom as compared to the unsubstituted one, the methyl-substituted thiazoldine **3k** having the methyl group attached to the exocyclic nitrogen was the sole product according to ^1^H NMR spectroscopy.

In contrast, the tautomeric ratio was notably changed. For the alkyl-substituted thiazolidines **3h–k** an **A**:**I** ratio of 4:1 was found according to the ^1^H NMR spectra. The exocyclic NH protons of the **A** forms gave signals at δ 9.12–9.20 ppm with ^3^*J* = 4.4–7.5 Hz, whereas a broad signal for the NH proton of the **I** form was observed more downfield (δ 9.49–9.68 ppm). Thus, the **A** form is more favorable for alkyl-substituted thiazolidines, and therefore the conjugation of the NH–C=N–C=O chain is likely more efficient than that of the N=C–NH–C=O fragment.

Aiming to improve the yield of thiazolidine **3k**, we performed this reaction under milder conditions. Surprisingly, an inverse regioselectivity was observed when the reaction was carried out at room temperature, and thiazolidine **4k** with the methyl group at the endocyclic nitrogen was obtained as a single product (Table 2). According to the ^1^H NMR spectrum of the reaction mixture, only trace amounts of thiazolidine **3k** were formed (<10% of **4k**). The thiazolidine **4k** exists in a single form, and no signs of any dynamic process were observed by NMR spectroscopy. Its structure was confirmed by the ^13^C,^1^H-HMBC spectrum that contained similar correlations between the methyl protons and both C=O and C=N carbon signals. Besides, these two carbon signals for **4k** are found more upfield than those for **3k** (by 14.4 ppm for C=O and 22.3 ppm for C=N).

**Table 2:** Reaction of monoalkylthioureas **1d–f** with *N*-phenylmaleimide (**2a**) at room temperature*.*

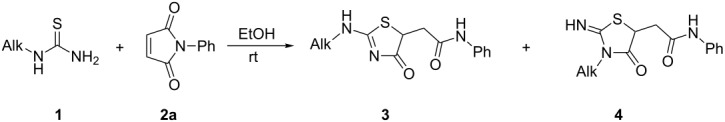				

Thiourea	Alk	Thiazolidine	Isolated yield, %	Ratio **3**:**4**^a^

**1d**	*c*-Hex	**3h**	91	1:0
**1e**	Et	**3j** + **4j**	68	0.6:1
**1f**	Me	**4k**	66	<0.1:1

^a^According to the ^1^H NMR spectra of the reaction mixtures.

Having obtained this unexpected result, we performed reactions of two other alkyl-substituted thioureas **1d,e** with *N*-phenylmaleimide (**2a**) at room temperature (Table 2). *N*-Ethylthiourea (**1e**) gave a mixture of isomers **3j** and **4j**, and *N*-cyclohexylthiourea (**1d**) – only isomer **3h** with the alkyl group on the exocyclic nitrogen atom. Steric factors seem to account for this result. Even a small size increase of an alkyl group from methyl to ethyl reduced selectivity, and cyclohexyl group yielded a single isomer.

Thiazolidine **4k** with the endocyclic nitrogen substituted with a methyl group can isomerize into isomer **3k** when refluxed in ethanol (ratio **4k**:**3k** after 2 h was 1:0.9, after 4 h it changed to 1:2.4). Whereas the formation of the isomer **4k** corresponds to the relative nucleophilicity of the nitrogen atoms of thiourea **1f**, the isomer **3k** seems to be more thermodynamically stable. The choice of the solvent turned out to be crucial for the isomerization: no change was observed after thiazolidine **4k** was heated in dioxane, even with addition of AcOH, and almost the same was observed in acetonitrile (ratio **4k**:**3k** ≈ 1:0.09 after 4 h). The reactions of both thioureas **1e** and **1f** with *N*-phenylmaleimide (**2a**) in dioxane either at room temperature or under reflux conditions provided mixtures of isomeric thiazolidines with prevalence of isomers with the alkyl group on the exocyclic nitrogen atom, respectively. Thus, the formation of thiazolidines is very sensitive to solvent properties as we demonstrated earlier [26].

DFT calculations (B3LYP/6-31+G(d,p)) confirm all conclusions given before pertaining to the effectiveness of conjugation and stability of thiazolidinones (Table 3). Isomers with an exocyclic position of the substituent R are the most stable, be it an aryl or an alkyl group. Only for R = Me or Et the isomers with the substituents bound to the endocyclic nitrogen atom are rather stable as we have observed for thiazolidines **4j,k**; in other cases its formation is unfavorable. Among pairs of tautomers, the phenyl-substituted ones have the minimum difference in stability, which agrees with the observed equimolar **A**:**I** ratio in solutions of the aryl-substituted thiazolidines **3a–f** (Scheme 2, Table 1). The less stable **I** form, when R is an alkyl group, accounts for the tautomeric ratio change in favor of the **A** form (**A**:**I** ratio for thiazolidines **3h–k** ≈ 4:1, Table 1). Only for R = 4-O_2_NC_6_H_4_ the **I** form is more stable than the **A** form which was indeed observed in case of thiazolidine **3g** (**A**:**I** ratio ≈ 1:1.8).

**Table 3:** Relative Gibbs free energies of the optimized conformations of model thiazolidines, Δ*G* (kcal/mol) [DFT B3LYP/6-31+G(d,p)), 298 K, DMSO (PCM)].

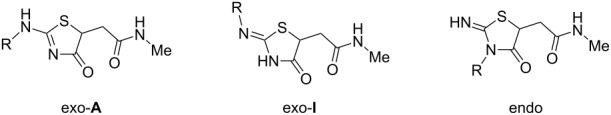			

R	exo-**A**	exo-**I**	endo

Ph	0	0.18	4.03
4-O_2_NC_6_H_4_	1.26	0	7.04
Me	0	3.33	2.68
Et	0	3.48	2.82
*c*-Hex	0	5.05	7.17

## Conclusion

We have demonstrated that the addition of monosubstituted thioureas to maleimides proceeds regioselectively to form thiazolidinones with an exocyclic position of the parent thiourea’s substituent for both aryl and alkyl groups. The substituent at the maleimide nitrogen atom has no influence on the reaction selectivity. However, the substituent on the parent thiourea determines the ratio of two tautomeric forms of the thiazolidines in solution. If it is an aryl group, the comparable stability of both forms results in an equimolar ratio of tautomers, except for the case of a strong electron-withdrawing group which changes the ratio in favor of the imino tautomer. The amino form prevails for alkyl-substituted thiazolidines.

Reactions of *N*-methyl- and *N*-ethylthioureas depend on the solvent and temperature. The best regioselectivity can be achieved in ethanol: at room temperature the less stable thiazolidines with the endocyclic nitrogen substituted by the alkyl group are formed preferentially. The reported data have revealed the delicate balance of electronic and steric factors determining the result of this seemingly simple reaction and can be used for the targeted synthesis of various aminothiazolidinones.

## Supporting Information

File 1 Experimental procedures, characterization data and copies of the ^1^H, ^13^C and 2D NMR spectra; X-ray analysis data for thiazolidine **3b**.
