# Supplementary material for: Thiazol-4-one derivatives from the reaction of monosubstituted thioureas with maleimides: structures and factors determining the selectivity and tautomeric equilibrium in solution
Source: Beilstein J Org Chem. 2016 Nov 29;12:2563–9. doi: 10.3762/bjoc.12.251 (PMC5238611; doi:10.3762/bjoc.12.251)
Supplement: File 1 — Experimental procedures, characterization data and copies of the 1H, 13C and 2D NMR spectra; X-ray analysis data for thiazolidine 3b. [file Beilstein_J_Org_Chem-12-2563-s001.pdf]

Supporting Information

for

Thiazol-4-one derivatives from the reaction of monosubstituted thioureas with maleimides: structures and factors determining the selectivity and tautomeric equilibrium in solution

Alena S. Pankova[1]*, Pavel R. Golubev[1], Alexander F. Khlebnikov[1], Alexander Yu. Ivanov[2], and Mikhail A. Kuznetsov[1]

Address: [1]Institute of Chemistry, Saint Petersburg State University, Universitetsky pr. 26, Saint Petersburg, 198504 Russia and [2]Centre for Magnetic Resonance, Saint Petersburg State University, Universitetsky pr. 26, Saint Petersburg, 198504 Russia

Email: Alena S. Pankova* - a.pankova@spbu.ru

*Corresponding author

Experimental procedures, characterization data and copies of the ^{1}H, ^{13}C and 2D NMR spectra; X-ray analysis data for thiazolidine **3b**

General information S2

Experimental procedures and characterization data S2

Copies of NMR spectra S11

X-ray analysis for thiazolidine **3b** S45

References S48

General information: NMR spectroscopic data were recorded with Bruker Avance 400 and 500 spectrometers (400.13 and 500.03 MHz for ^{1}H, 100.61 and 125.76 MHz for ^{13}C, respectively) in DMSO-d_6. Spectra were referenced to the solvent residual proton (δ_H = 2.50 ppm) and solvent carbon signals (δ_C = 39.52 ppm). DEPT spectra were used for carbon atom signal assignment. The signals of the aryl ring of the parent thiourea are referred to as "A" and the ones of the aryl ring of the parent maleimide as "B". The diastereotopic protons of the CH_2 group of thiazolidinones are referred to as H^a and H^b. Melting points were determined with a Stuart SMP30 instrument. Mass spectra were recorded with a Bruker Maxis HRMS-ESI-qTOF spectrometer (electrospray ionization, ESI, mode). Single crystal X-ray data were obtained using an Agilent Technologies SuperNova Atlas diffractometer. For TLC analysis Alugram SIL G/UV$_{254}$ (Macherey-Nagel) plates were used.

Compound 3a

The stirred mixture of *N*-phenylthiourea (**1a**, 152 mg, 1 mmol) and *N*-phenylmaleimide (**2a**, 173 mg, 1 mmol) in EtOH (5 mL) was refluxed for 3 h. Then it was poured into water (40 mL), the formed precipitate was collected by filtration, washed with water and recrystallized from EtOH/water 1:1. Colorless crystals, yield 250 mg (77%); mp 216–217 °C (dec.) (lit. mp 216.5-217.5 °C (dec.) [1]). According to its NMR spectra, amide **3a** exists as a tautomeric mixture of 2-(4-oxo-2-phenylimino-1,3-thiazolidin-5-yl)-*N*-phenylacetamide (**3a**-**I**) and 2-(2-anilino-4-oxo-4,5-dihydro-1,3-thiazol-5-yl)-*N*-phenylacetamide (**3a**-**A**) in a 1:1 ratio. 2D NOESY, ^{13}C-^{1}H HSQC and HMBC, ^{15}N-^{1}H HSQC and HMBC spectra were used for signals assignment.

^{1}H NMR (400 MHz, DMSO-d_6): δ = 2.78-2.92 (m, 2 H, H^a); 3.22-3.33 (m, 2 H, H^b); 4.49-4.51 (m, 2 H, 2 SCH); 6.99-7.04 (m, 4 H, H^{Ar}); 7.14-7.16 (m, 2 H, H^{Ar}); 7.29-7.39 (m, 8 H, H^{Ar}); 7.52-7.58 (m, 4 H, H^{Ar}); 7.70-7.72 (m, 2 H, H^{Ar}); 10.11 (s, 1 H, $Ph^BN\underline{H}$); 10.14 (s, 1 H, $Ph^BN\underline{H}$); 11.16 (br s, 1 H, NH); 11.74 (br s, 1 H, NH) ppm.

^{1}H NMR (500 MHz, acetone-d_6/DMSO-d_6 3:1, 0 °C): δ = 2.84 (dd, J = 16.7, 11.4 Hz, 1 H, H^a of **A**); 2.94 (dd, J = 16.7, 10.7 Hz, 1 H, H^a of **I**); 3.35 (dd, J = 16.7, 3.3 Hz, 1 H, H^b of **I**); 3.44 (dd, J = 16.7, 3.3 Hz, 1 H, H^b of **A**); 4.50 (dd, J = 10.7, 3.3 Hz, 1 H, SCH of **I**); 4.51 (dd, J = 11.4, 3.3 Hz, 1 H, SCH of **A**); 7.01-7.06 (m, 4 H, H^{oA} of **I**, 2 H^{pB}); 7.11-7.17 (m, 2 H, 2 H^{pA}); 7.26-7.32 (m, 4 H, 2 H^{mB}); 7.34-7.37 (m, 2 H, H^{mA} of **I**); 7.37-7.40 (m, 2 H, H^{mA} of **A**); 7.61 (d, J = 8.2 Hz, 2 H, H^{oB}); 7.66 (d, J = 8.2 Hz, 2 H, H^{oB}); 7.81 (d, J = 8.1 Hz, 2 H, H^{oA} of **A**);

10.19 (s, 1 H, Ph^BNH); 10.23 (s, 1 H, Ph^BNH); 11.23 (s, 1 H, $Ph^AN\underline{H}$ of **A**); 11.83 (s, 1 H, NH of **I**) ppm.
^{13}C NMR (100 MHz, DMSO-d_6): δ = 39.0 (CH_2); 39.6 (CH_2); 46.0 (SCH of **I**); 50.0 (SCH of **A**); 119.1 (C^{oB}); 120.4 (C^{oA} of **A**), 121.6 (C^{oA} of **I**); 123.4 (C^{pB}); 124.6 (C^{pA}); 124.7 (C^{pA}); 128.7 (C^{mB}); 129.0 (C^{mA}), 129.2 (C^{mA}); 138.7 (C^i); 138.8 (C^i); 146.3 (br s, C=N of **I**); 160.7 (br s, C=N of **A**); 168.0 (Ph^BNHC=O of **I**); 168.5 (Ph^BNHC=O of **A**); 177.9 (C=O of **I**); 189.5 (C=O of **A**) ppm.
HRMS (ESI), *m/z*: calcd for $C_{17}H_{15}N_3O_2S$ $[M+H]^+$ 326.0958, found 326.0962.

Compound 3b

The stirred mixture of *N*-phenylthiourea (**1a**, 152 mg, 1 mmol) and *N*-ethylmaleimide (**2b**, 125 mg, 1 mmol) in EtOH (5 mL) was refluxed for 3 h. Then it was poured into water (40 mL), the formed precipitate was collected by filtration, washed with water and recrystallized from EtOH/iPrOH/dioxane 1:1:0.3. Colorless crystals, yield 195 mg (70%), mp 224-226 °C (dec.) (lit. mp 237 °C (dec.) [1]). When this reaction was performed at room temperature (stirring for 4 d [1]) product **3b** was obtained in 67% yield. According to the ^1H NMR spectrum, amide **3b** exists as tautomeric mixture of *N*-ethyl-2-(4-oxo-2-phenylimino-1,3-thiazolidine-5-yl)acetamide (**3b-I**) and 2-(2-anilino-4,5-dihydro-4-oxo-1,3-thiazol-5-yl)-*N*-ethylacetamide (**3b-A**) in a 1:1 ratio. 2D NOESY and ^{15}N-^1H HMBC spectra were used for signals assignment.
^1H NMR (400 MHz, DMSO-d_6): δ = 0.96-1.03 (m, 6 H, 2 CH_3); 2.46-2.60 (m, 2 H, 2 H^a); 2.93-3.10 (m, 6 H, 2 NCH_2, 2 H^b); 4.36-4.38 (m, 2 H, 2 SCH); 6.97-6.99 (m, 2 H, H^o of **I**); 7.14-7.16 (m, 2 H, 2 H^p); 7.34-7.40 (m, 4 H, H^m); 7.69-7.71 (m, 2 H, H^o of **A**); 8.01-8.04 (m, 2 H, 2 EtN$\underline{H}$); 11.11 (br s, 1 H, PhN$\underline{H}$ of **A**); 11.69 (br s, 1 H, NH of **I**) ppm.
^1H NMR (500 MHz, $CDCl_3$/DMSO-d_6 2.5:1, 0 °C): δ = 0.97 (t, *J* = 7.3 Hz, 3 H, CH_3); 1.01 (t, *J* = 7.3 Hz, 3 H, CH_3); 2.45 (dd, *J* = 16.7, 12.0 Hz, 2 H, 2 H^a); 3.01-3.13 (m, 6 H, 2 NCH_2, 2 H^b); 4.21-4.27 (m, 2 H, 2 SCH); 6.93 (d, *J* = 7.6 Hz, 2 H, H^o of **I**); 7.02-7.05 (m, 2 H, 2 H^p); 7.21-7.25 (m, 4 H, H^m); 7.65 (d, *J* = 7.9 Hz, 2 H, H^o of **A**); 7.89 (br s, 1 H, EtN$\underline{H}$); 7.92 (br s, 1 H, EtN$\underline{H}$); 11.00 (s, 1 H, PhN$\underline{H}$ of **A**); 11.68 (s, 1 H, NH of **I**) ppm.
^{13}C NMR (100 MHz, DMSO-d_6): δ = 14.6 (CH_3); 33.5 (NCH_2); 38.0 (CH_2); 38.6 (CH_2); 46.3 (SCH); 50.4 (SCH); 120.4 (C^o); 121.5 (C^o); 124.6 (C^p); 124.7 (C^p); 129.0 (C^m); 129.2 (C^m); 138.7 (br s, C^i); 146.4 (br s, C=N); 168.5 (EtNHC=O); 169.1 (EtNHC=O); 178.0 (C=O); 189.5 (C=O) ppm.
HRMS (ESI), *m/z*: calcd for $C_{13}H_{15}N_3O_2S$ $[M+H]^+$ 278.0958, found 278.0961.

Compound 3c

The stirred mixture of *N*-phenylthiourea (**1a**, 152 mg, 1 mmol) and *N*-(4-ethoxyphenyl)maleimide (**2c**, 217 mg, 1 mmol) in EtOH (5 mL) was refluxed for 3 h. Then it was poured into water (40 mL), the formed precipitate collected by filtration, washed with water and recrystallized from EtOH/water 1:1. Colorless crystals, yield 244 mg (66%), mp 224-225 °C (dec.). According to the ^{1}H NMR spectrum, amide **3c** exists as tautomeric mixture of *N*-(4-ethoxyphenyl)-2-(4-oxo-2-phenylimino-1,3-thiazolidin-5-yl)acetamide (**3c-I**) and 2-(2-anilino-4-oxo-4,5-dihydro-1,3-thiazol-5-yl)-*N*-(4-ethoxyphenyl)acetamide (**3c-A**) in a 1:1 ratio.

^{1}H NMR (400 MHz, DMSO-d_6): δ = 1.29 (br s, 6 H, 2 CH_3); 2.74-2.88 (m, 2 H, 2 H^a); 3.18-3.32 (m, 2 H, 2 H^b); 3.96 (br s, 4 H, 2 CH_2); 4.47-4.50 (m, 2 H, 2 SCH); 6.85 (br s, 4 H, H^{mB}); 7.00 (br s, 2 H, H^{oA} of **I**); 7.14-7.16 (m, 2 H, H^{pA}); 7.35-7.47 (m, 8 H, H^{Ar}); 7.70-7.72 (m, 2 H, H^{oA} of **A**); 9.97 (s, 1 H, Ar^BN<u>H</u>); 10.00 (s, 1 H, Ar^BN<u>H</u>); 11.17 (br s, 1 H, NH); 11.74 (br s, 1 H, NH) ppm.

^{13}C NMR (100 MHz, DMSO-d_6): δ = 14.7 (CH_3); 38.8 (br s, CH_2); 39.4 (br s, CH_2); 46.1 (br s, SCH); 50.1 (br s, SCH); 63.1 (OCH_2); 114.4 (C^{mB}); 120.4 (br s, C^{oA}); 120.6 (C^{oB}); 121.6 (br s, C^{oA}); 124.7 (br s, C^{pA}); 129.0 (br s, C^{mA}); 129.2 (br s, C^{mA}); 131.8 (br s, C^{iB}); 131.9 (br s, C^{iB}); 138.7 (br s, C^{iA}); 154.5 (C^{pB}); 167.4 (br s, Ar^BNHC=O); 167.9 (br s, Ar^BNHC=O); 177.9 (br s, C=O); 189.5 (br s, C=O) ppm. Other carbon signals cannot be clearly detected.

HRMS (ESI), *m/z*: calcd for $C_{19}H_{19}N_3O_3S$ $[M+H]^+$ 370.1220, found 370.1213.

Compound 3d

The stirred mixture of *N*-phenylthiourea (**1a**, 152 mg, 1 mmol) and *N*-(4-nitrophenyl)maleimide (**2d**, 218 mg, 1 mmol) in EtOH (5 mL) was refluxed for 3 h. Then it was poured into water (40 mL), the formed precipitate collected by filtration, washed with water and recrystallized from EtOH/water 1:1. Colorless crystals, yield 331 mg (89%), mp 226-228 °C (dec.). According to ^{1}H NMR spectrum, amide **3d** exists as tautomeric mixture of *N*-(4-nitrophenyl)-2-(4-oxo-2-phenylimino-1,3-thiazolidin-5-yl)acetamide (**3d-I**) and 2-(2-anilino-4-oxo-4,5-dihydro-1,3-thiazol-5-yl)-*N*-(4-nitrophenyl)acetamide (**3d-A**) in a 1:1 ratio.

^{1}H NMR (400 MHz, DMSO-d_6): δ = 2.90-3.03 (m, 2 H, 2 H^a); 3.29-3.41 (m, 2 H, 2 H^b); 4.52-4.54 (m, 2 H, 2 SCH); 7.00 (d, J = 6.9 Hz, 2 H, H^{oA} of **I**); 7.14-7.18 (m, 2 H, H^{pA}); 7.33-7.41 (m, 4 H, H^{mA}); 7.71 (d, J = 7.4 Hz, 2 H, H^{oA} of **A**); 7.76-7.83 (m, 4 H, H^{oB}); 8.19-8.23 (m, 4

H, 4 H^{mB}); 10.74 (s, 1 H, $Ar^{B}N\underline{H}$); 10.77 (s, 1 H, $Ar^{B}N\underline{H}$); 11.18 (br s, 1 H, NH); 11.78 (br s, 1 H, NH) ppm.
^{13}C NMR (100 MHz, DMSO-d_6): δ = 39.1 (br s, CH_2); 45.7 (br s, SCH); 49.6 (br s, SCH); 118.8 (C^{oB}); 120.4 (br s, C^{oA}); 121.6 (br s, C^{oA}); 124.7 (br s, C^{pA}); 125.0 (C^{mB}); 129.0 (br s, C^{mA}); 129.2 (br s, C^{mA}); 138.7 (br s, C^{iA}); 142.3 (C^{pB}); 144.8 (br s, C^{iB}); 144.9 (br s, C^{iB}); 169.2 (br s, Ar^{B}NHC=O); 169.7 (br s, Ar^{B}NHC=O); 177.7 (br s, C=O); 189.3 (br s, C=O) ppm. Other carbon signals cannot be clearly detected.
HRMS (ESI), *m/z*: calcd for $C_{17}H_{14}N_4O_4S$ $[M+Na]^+$ 393.0628, found 393.0617.

Compound 3e

The stirred mixture of *N*-phenylthiourea (**1a**, 152 mg, 1 mmol) and *N*-cyclohexylmaleimide (**2e**, 179 mg, 1 mmol) in EtOH (5 mL) was refluxed for 6 h, then stirred at rt for 24 h. Then it was poured into water (40 mL), the formed precipitate collected by filtration, washed with water and recrystallized from EtOH/water 1:1. Colorless crystals, yield 186 mg (56%), mp 237-239 °C (dec.). According to ^{1}H NMR spectrum, amide **3e** exists as tautomeric mixture of *N*-cyclohexyl-2-(4-oxo-2-phenylimino-1,3-thiazolidin-5-yl)acetamide (**3e-I**) and 2-(2-anilino-4-oxo-4,5-dihydro-1,3-thiazol-5-yl)-*N*-cyclohexylacetamide (**3e-A**) in a 1:1 ratio.
^{1}H NMR (400 MHz, DMSO-d_6): δ = 1.11-1.23 (m, 10 H, c-Hex); 1.52-1.70 (m, 10 H, c-Hex); 2.45-2.60 (m, 2 H, 2 H^a); 2.92-3.05 (m, 2 H, 2 H^b); 3.49 (br s, 2 H, 2 NCH); 4.35-4.38 (m, 2 H, 2 SCH); 6.97-6.99 (m, 2 H, H^o of **I**); 7.14 (br s, 2 H, H^p); 7.36 (br s, 4 H, H^m); 7.69 (br s, 2 H, H^o of **A**); 7.88-7.93 (m, 2 H, 2 c-HexNH); 11.14 (br s, 1 H, NH); 11.64 (br s, 1 H, NH) ppm.
^{13}C NMR (100 MHz, DMSO-d_6): δ = 24.4 (c-Hex-$C^{3,5}$); 25.2 (c-Hex-C^4); 32.3 (c-Hex-$C^{2,6}$); 38.0 (br s, CH_2); 38.7 (br s, CH_2); 46.3 (br s, SCH); 47.7 (NCH); 50.5 (br s, SCH); 120.4 (C^o); 121.5 (C^o); 124.6 (br s, C^p); 129.0 (C^m); 129.2 (C^m); 138.7 (br s, C^i); 167.8 (br s, c-HexNHC=O); 168.3 (br s, c-HexNHC=O); 178.0 (br s, C=O); 189.6 (br s, C=O) ppm.
HRMS (ESI), *m/z*: calcd for $C_{17}H_{21}N_3O_2S$ $[M+H]^+$ 332.1427, found 332.1429.

Compound 3f

The stirred mixture of *N*-(4-methoxyphenyl)thiourea (**1b**, 182 mg, 1 mmol) and *N*-phenylmaleimide (**2a**, 260 mg, 1.5 mmol) in EtOH (5 mL) was refluxed for 7 h, then stirred at rt for 24 h. Then it was poured into water (40 mL), the formed precipitate collected by filtration, washed with water and recrystallized from iPrOH. Colorless crystals, yield 230 mg

(65%), mp 208-209 °C (dec.). According to ^{1}H NMR spectrum, amide **3f** exists as tautomeric mixture of 2-(2-(4-methoxyphenyl)imino-4-oxo-1,3-thiazolidin-5-yl)-*N*-phenylacetamide (**3f-I**) and 2-(2-(4-methoxyphenyl)amino-4-oxo-4,5-dihydro-1,3-thiazol-5-yl)-*N*-phenylacetamide (**3f-A**) in a 1:1 ratio.

^{1}H NMR (400 MHz, DMSO-d_6): δ = 2.75-2.87 (m, 2 H, H^{a}); 3.21-3.33 (m, 2 H, H^{b}); 3.74 (s, 3 H, OCH_3); 3.75 (s, 3 H, OCH_3); 4.44-4.48 (m, 2 H, 2 SCH); 6.92-7.07 (m, 8 H, H^{Ar}); 7.26-7.32 (m, 4 H, 2 H^{mB}); 7.53 (d, *J* = 7.9 Hz, 2 H, H^{oB}); 7.57 (d, *J* = 7.9 Hz, 2 H, H^{oB}); 7.61 (d, *J* = 9.0 Hz, 2 H, H^{oA} of **A**); 10.10 (s, 1 H, Ph^{B}N<u>H</u>); 10.14 (s, 1 H, Ph^{B}N<u>H</u>); 11.04 (br s, 1 H, NH); 11.58 (br s, 1 H, NH) ppm.

^{13}C NMR (100 MHz, DMSO-d_6): δ = 39.2 (CH_2); 39.7 (CH_2); 50.1 (SCH); 55.2 (CH_3); 55.3 (CH_3); 114.1 (C^{mA}); 114.5 (C^{mA}); 119.07 (C^{oB}); 119.10 (C^{oB}); 122.0 (C^{oA}); 123.4 (C^{pB}); 123.6 (br s, C^{oA}); 128.7 (C^{mB}); 131.9 (C^{iA}); 138.7 (C^{iB}); 138.8 (C^{iB}); 156.3 (C^{pA}); 156.8 (C^{pA}); 168.1 (Ph^{B}NHC=O); 168.6 (Ph^{B}NHC=O); 177.1 (C=O); 189.2 (C=O) ppm. Other carbon signals cannot be clearly detected.

HRMS (ESI), *m/z*: calcd for $C_{18}H_{17}N_3O_3S$ $[M+H]^+$ 356.1063, found 356.1077.

Compound 3g

The stirred mixture of *N*-(4-nitrophenyl)thiourea (**1c**, 197 mg, 1 mmol) and *N*-phenylmaleimide (**2a**, 260 mg, 1.5 mmol) in EtOH (6 mL) was refluxed for 8 h, then stirred at rt for 48 h. Then it was poured into water (40 mL), the formed precipitate collected by filtration, washed with water and recrystallized from iPrOH. Pale yellow crystals, yield 270 mg (73%), mp 220-222 °C (dec.). According to ^{1}H NMR spectrum, amide **3g** exists as tautomeric mixture of 2-(2-(4-nitrophenyl)imino-4-oxo-1,3-thiazolidin-5-yl)-*N*-phenylacetamide (**3g-I**) and 2-(2-(4-nitrophenyl)amino-4-oxo-4,5-dihydro-1,3-thiazol-5-yl)-*N*-phenylacetamide (**3g-A**) in a ~1.8:1 ratio at 23 °C.

^{1}H NMR (400 MHz, DMSO-d_6, 23 °C): δ = 2.967 (br s, 1 H, H^{a}); 3.26-3.33 (m, 1 H, H^{b}); 4.58-4.60 (m, 1 H, SCH); 7.02-7.06 (m, 1 H, H^{pB}); 7.16 (br s, 1.28 H, H^{oA} of **I**); 7.27-7.31 (m, 2 H, H^{mB}); 7.53-7.55 (m, 2 H, H^{oB}); 7.98 (br s, 0.72 H, H^{oA} of **A**); 8.24 (br s, 2 H, H^{mA}); 10.15 (s, 1 H, Ph^{B}N<u>H</u>); 11.70 (br s, 0.36 H, NH of **A**); 12.02 (br s, 0.64 H, NH of **I**) ppm.

^{1}H NMR (500 MHz, acetone-d_6/DMSO-d_6 3:1, -20 °C): δ = 2.91 (dd, *J* = 16.5, 11.7 Hz, 0.39 H, H^{a} of **A**); 3.04 (dd, *J* = 16.9, 10.6 Hz, 0.61 H, H^{a} of **I**); 3.38-3.47 (m, 1 H, H^{b}); 4.53-4.56 (m, 0.39 H, SCH of **A**); 4.59 (dd, *J* = 10.6, 2.9 Hz, 0.61 H, SCH of **I**); 7.01-7.05 (m, 1 H, H^{pB}); 7.19 (d, *J* = 8.6 Hz, 1.22 H, H^{oA} of **I**); 7.25-7.31 (m, 2 H, H^{mB}); 7.60 (d, *J* = 7.9 Hz, 1.22 H, H^{oB} of **I**); 7.66 (d, *J* = 7.8 Hz, 0.78 H, H^{oB} of **A**); 8.09 (d, *J* = 8.8 Hz, 0.78 H, H^{oA} of **A**);

8.25 (d, J = 8.6 Hz, 1.22 H, H^{mA} of **I**); 8.32 (d, J = 8.8 Hz, 0.78 H, H^{mA} of **A**); 10.27 (s, 0.61 H, $Ph^{B}N\underline{H}$ of **I**); 10.30 (s, 0.39 H, $Ph^{B}N\underline{H}$ of **A**); 11.81 (br s, 0.39 H, NH of **A**); 12.17 (br s, 0.61 H, NH of **I**) ppm.

^{1}H NMR (400 MHz, DMSO-d_6, 120 °C): δ = 2.91 (dd, J = 16.4, 9.7 Hz, 1 H, H^{a}); 3.29 (dd, J = 16.4, 3.9 Hz, 1 H, H^{b}); 4.58 (dd, J = 9.7, 3.9 Hz, 1 H, SCH); 7.06 (t, J = 7.4 Hz, 1 H, H^{pB}); 7.27-7.31 (m, 2 H, H^{mB}); 7.44 (br s, 2 H, H^{oA}); 7.52-7.54 (m, 2 H, H^{oB}); 8.20 (d, J = 9.0 Hz, 2 H, H^{mA}); 9.74 (s, 1 H, $Ph^{B}N\underline{H}$); 11.50 (br s, 1 H, NH) ppm.

^{13}C NMR (100 MHz, DMSO-d_6, 23 °C): δ = 45.5 (br s, SCH); 119.1 (C^{oB}); 122.1 (br s, C^{oA}); 123.4 (C^{pB}); 125.2 (C^{mA}); 128.8 (C^{mB}); 138.7 (C^{iB}); 143.5 (br s, C); 167.9 (br s, Ph^{B}NHC=O) ppm. Other carbon signals cannot be clearly detected.

HRMS (ESI), *m/z*: calcd for $C_{17}H_{14}N_4O_4S$ $[M+Na]^+$ 393.0628, found 393.0627.

Compound 3h

The stirred mixture of *N*-cyclohexylthiourea (**1d**, 158 mg, 1 mmol) and *N*-phenylmaleimide (**2a**, 173 mg, 1 mmol) in EtOH (5 mL) was refluxed for 4 h. Then it was poured into water (40 mL), the formed precipitate collected by filtration, washed with water and recrystallized from EtOH/water 1:1. Colorless crystals, yield 264 mg (80%), mp 209-210 °C. When this reaction was performed at rt (stirring for 48 h) amide **3h** was obtained in 91% yield (pure enough without recrystallization). According to ^{1}H NMR spectrum, amide **3h** exists as tautomeric mixture of 2-(2-cyclohexylamino-4-oxo-4,5-dihydro-1,3-thiazol-5-yl)-*N*-phenylacetamide (**3h-A**) and 2-(2-cyclohexylimino-4-oxo-1,3-thiazolidin-5-yl)-*N*-phenylacetamide (**3h-I**) in a ~4:1 ratio.

^{1}H NMR (400 MHz, DMSO-d_6): δ = 1.12-1.33 (m, 5 H, c-Hex); 1.55-1.58 (m, 1 H, c-Hex); 1.69-1.71 (m, 2 H, c-Hex); 1.86-1.88 (m, 2 H, c-Hex); 2.67 (dd, J = 16.4, 11.3 Hz, 1 H, H^{a}); 3.23 (br s, 0.2 H, NCH of **I**); 3.25 (dd, J = 16.4, 3.4 Hz, 1 H, H^{b}); 3.79 (br s, 0.8 H, NCH of **A**); 4.36 (dd, J = 11.3, 3.4 Hz, 1 H, SCH); 7.04 (t, J = 7.4 Hz, 1 H, H^{p}); 7.28-7.32 (m, 2 H, H^{m}); 7.55 (d, J = 7.8 Hz, 2 H, H^{o}); 9.14 (d, J = 7.3 Hz, 0.8 H, c-HexN<u>H</u> of **A**); 9.68 (br s, 0.2 H, NH of **I**); 10.09 (s, 0.8 H, PhN<u>H</u> of **A**); 10.12 (s, 0.2 H, PhN<u>H</u> of **I**) ppm.

^{13}C NMR of **3h-A** (100 MHz, DMSO-d_6): δ = 24.3 (c-Hex-$C^{3,5}$); 24.9 (c-Hex-C^{4}); 31.9 (c-Hex-$C^{2,6}$); 40.1 (CH_2); 50.6 (SCH); 53.7 (NCH); 119.1 (C^{o}); 123.3 (C^{p}); 128.7 (C^{m}); 138.9 (C^{i}); 168.7 (PhNHC=O); 178.4 (C=N); 188.3 (C=O) ppm.

HRMS (ESI), *m/z*: calcd for $C_{17}H_{21}N_3O_2S$ $[M+H]^+$ 332.1427, found 332.1429.

Compound 3i

The stirred mixture of *N*-cyclohexylthiourea (**1d**, 158 mg, 1 mmol) and *N*-ethylmaleimide (**2b**, 125 mg, 1 mmol) in EtOH (5 mL) was refluxed for 4 h. Then it was poured into water (40 mL), the resulting solution was saturated with NaCl and extracted with EtOAc (3 × 10 mL). The organic phase was dried over Na_2SO_4, solvents were evaporated under reduced pressure until 5 mL remained. Upon cooling to ~0 °C the formed precipitate was collected by filtration, washed with cold EtOAc (~3 mL) and dried. Colorless crystals, yield 210 mg (74%), mp 161-163 °C. According to ^1H NMR spectrum, amide **3i** exists as tautomeric mixture of 2-(2-cyclohexylamino-4-oxo-4,5-dihydro-1,3-thiazol-5-yl)-*N*-ethylacetamide (**3i-A**) and 2-(2-cyclohexylimino-4-oxo-1,3-thiazolidin-5-yl)-*N*-ethylacetamide (**3i-I**) in a ~4:1 ratio.

^1H NMR (400 MHz, DMSO-d_6): δ = 1.00 (t, J = 7.2 Hz, CH_3 of **A**) and 1.01 (t, J = 7.2 Hz, CH_3 of **I**), 3 H in sum; 1.09-1.18 (m, 1 H, c-Hex); 1.21-1.32 (m, 4 H, c-Hex); 1.55-1.58 (m, 1 H, c-Hex); 1.68-1.71 (m, 2 H, c-Hex); 1.84-1.86 (m, 2 H, c-Hex); 2.36 (dd, J = 15.9, 11.6 Hz, 1 H, H^a); 2.96 (dd, J = 15.9, 3.4 Hz, 1 H, H^b); 3.03-3.10 (m, 2 H, NCH_2); 3.18 (br s, 0.2 H, NCH of **I**); 3.76 (br s, 0.8 H, NCH of **A**); 4.23 (dd, J = 11.6, 3.4 Hz, 1 H, SCH); 7.99-8.02 (m, 1 H, EtNH); 9.12 (d, J = 7.0 Hz, 0.8 H, NH of **A**); 9.63 (br s, 0.2 H, NH of **I**) ppm.

^{13}C NMR of **3i-A** (100 MHz, DMSO-d_6): δ = 14.6 (CH_3); 24.3 (c-Hex-$C^{3,5}$); 24.9 (c-Hex-C^4); 31.9 (c-Hex-$C^{2,6}$); 33.5 (NCH_2); 39.1 (CH_2); 50.9 (SCH); 53.7 (NCH); 169.2 (EtNHC=O); 178.4 (C=N); 188.4 (C=O) ppm.

HRMS (ESI), *m/z*: calcd for $C_{13}H_{21}N_3O_2S$ $[M+H]^+$ 284.1427, found 284.1433.

Compound 3j

The stirred mixture of *N*-ethylthiourea (**1e**, 156 mg, 1.5 mmol) and *N*-phenylmaleimide (**2a**, 260 mg, 1.5 mmol) in EtOH (6 mL) was refluxed for 3.5 h. After cooling, the formed precipitate was collected by filtration and washed with cold EtOH (1–2 mL). Colorless crystals, yield 276 mg (66%), mp 223-224 °C (dec.). According to ^1H NMR spectrum, amide **3j** exists as tautomeric mixture of 2-(2-ethylamino-4-oxo-4,5-dihydro-1,3-thiazol-5-yl)-*N*-phenylacetamide (**3j-A**) and 2-(2-ethylimino-4-oxo-1,3-thiazolidin-5-yl)-*N*-phenylacetamide (**3j-I**) in a ~4:1 ratio.

^1H NMR (400 MHz, DMSO-d_6): δ = 1.13 (t, J = 7.2 Hz, 2.4 H, CH_3 of **A**); 1.16 (t, J = 7.2 Hz, 0.6 H, CH_3 of **I**); 2.68 (dd, J = 16.4, 11.2 Hz, 1 H, H^a); 3.21-3.48 (m, 3 H, $C\underline{H}_2CH_3$, H^b); 4.36 (dd, J = 11.2, 3.4 Hz, 0.2 H, SCH of **I**); 4.38 (dd, J = 11.2, 3.5 Hz, 0.8 H, SCH of **A**); 7.04 (t,

J = 7.4 Hz, 1 H, H^p); 7.28-7.32 (m, 2 H, H^m); 7.55 (d, J = 7.8 Hz, 2 H, H^o); 9.20 (t, J = 5.2 Hz, 0.8 H, EtNH of **A**); 9.62 (br s, 0.2 H, NH of **I**); 10.10 (s, 0.8 H, PhNH of **A**); 10.12 (s, 0.2 H, PhNH of **I**) ppm.
^{13}C NMR (100 MHz, DMSO-d_6): δ = 14.1 (CH_3 of **A**); 14.3 (CH_3 of **I**); 39.2 (CH_2 of **I**); 39.3 (CH_2 of **A**); 39.8 (CH_2 of **I**); 40.1 (CH_2 of **A**); 50.6 (SCH of **I**); 50.8 (SCH of **A**); 119.1 (C^o); 123.3 (C^p); 128.7 (C^m); 138.8 (C^i); 168.7 (PhNHC=O); 179.2 (C=N); 188.2 (C=O) ppm.
HRMS (ESI), *m/z*: calcd for $C_{13}H_{15}N_3O_2S$ $[M+Na]^+$ 300.0777, found 300.0791.

Compound 3k

The stirred mixture of *N*-methylthiourea (**1f**, 135 mg, 1.5 mmol) and *N*-phenylmaleimide (**2a**, 260 mg, 1.5 mmol) in EtOH (6 mL) was refluxed for 4 h. Then it was poured into water (40 mL), the resulting solution was saturated with NaCl and extracted with EtOAc (3 × 10 mL). The organic phase was dried over Na_2SO_4, the solvents were evaporated under reduced pressure until 5 mL remained. Upon cooling to 0 °C, a precipitate began to form, which was isolated by slow addition of hexane (1–2 mL) and filtration. Pale yellow crystals, yield 198 mg (50%), mp 197-198 °C. According to ^{1}H NMR spectrum, amide **3k** exists as tautomeric mixture of 2-(2-methylamino-4-oxo-4,5-dihydro-1,3-thiazol-5-yl)-*N*-phenylacetamide (**3k-A**) and 2-(2-methylimino-4-oxo-1,3-thiazolidin-5-yl)-*N*-phenylacetamide (**3k-I**) in a ~4:1 ratio.
^{1}H NMR (400 MHz, DMSO-d_6): δ = 2.68 (dd, J = 16.4, 11.2 Hz, 1 H, H^a); 2.88 (s, 0.6 H, Me of **I**); 2.95 (d, J = 4.4 Hz, 2.4 H, Me of **A**); 3.26 (dd, J = 16.5, 3.5 Hz, 0.8 H, H^b of **A**); 3.28 (dd, J = 16.6, 3.6 Hz, 0.2 H, H^b of **I**); 4.36 (dd, J = 11.2, 3.6 Hz, 0.2 H, SCH of **I**); 4.39 (dd, J = 11.2, 3.5 Hz, 0.8 H, SCH of **A**); 7.04 (t, J = 7.4 Hz, 1 H, H^p); 7.28-7.32 (m, 2 H, H^m); 7.55 (d, J = 7.8 Hz, 2 H, H^o); 9.13 (q, J = 4.4 Hz, 0.8 H, MeNH of **A**); 9.49 (br s, 0.2 H, NH of **I**); 10.11 (s, 0.8 H, PhNH of **A**); 10.13 (s, 0.2 H, PhNH of **I**) ppm.
^{13}C NMR (100 MHz, DMSO-d_6): δ = 30.3 (CH_3 of **I**); 30.9 (CH_3 of **A**); 40.1 (CH_2 of **A**); 51.0 (SCH of **I**); 51.1 (SCH of **A**); 119.1 (C^o); 123.3 (C^p); 128.8 (C^m); 138.9 (C^i); 168.7 (PhNHC=O); 180.1 (C=N); 188.2 (C=O) ppm.
HRMS (ESI), *m/z*: calcd for $C_{12}H_{13}N_3O_2S$ $[M+H]^+$ 264.0801, found 264.0811.

2-(3-Ethyl-2-imino-4-oxo-1,3-thiazolidin-5-yl)-*N*-phenylacetamide (4j). The mixture of *N*-ethylthiourea (**1e**, 156 mg, 1.5 mmol) and *N*-phenylmaleimide (**2a**, 260 mg, 1.5 mmol) in EtOH (6 mL) was stirred at rt overnight. The precipitate was filtered and washed with cold

EtOH (1–2 mL). Yield 284 mg (68%). According to ^{1}H NMR spectrum, a mixture of **4j** and amide **3j** (ratio **3j-A/3j-I** ~4:1) in the ratio 1:0.6 was obtained.

^{1}H NMR of **4j** (400 MHz, DMSO-d_6): δ = 1.09 (t, J = 7.1 Hz, 3 H, CH_3); 2.89 (dd, J = 16.5, 9.5 Hz, 1 H, H^a); 3.22 (dd, J = 16.5, 3.7 Hz, 1 H, H^b); 3.58-3.70 (m, 2 H, C$\underline{H}_2$$CH_3$); 4.52 (dd, J = 9.5, 3.7 Hz, 1 H, SCH); 7.04 (t, J = 7.4 Hz, 1 H, H^p); 7.28-7.32 (m, 2 H, H^m); 7.54 (d, J = 7.7 Hz, 2 H, H^o); 9.16 (s, 1 H, =NH); 10.10 (s, 1 H, PhN$\underline{H}$) ppm.

^{13}C NMR of **4j** (100 MHz, DMSO-d_6): δ = 12.2 (CH_3); 36.2 (NCH_2); 39.1 (CH_2); 44.2 (SCH); 119.1 (C^o); 123.4 (C^p); 128.7 (C^m); 138.7 (C^i); 157.0 (C=NH); 167.8 (PhNHC=O); 173.5 (C=O) ppm.

HRMS (ESI), *m/z*: calcd for $C_{13}H_{15}N_3O_2S$ $[M+Na]^+$ 300.0777, found 300.0772.

2-(2-Imino-3-methyl-4-oxo-1,3-thiazolidin-5-yl)-*N*-phenylacetamide (4k). The mixture of *N*-methylthiourea (**1f**, 135 mg, 1.5 mmol) and *N*-phenylmaleimide (**2a**, 260 mg, 1.5 mmol) in EtOH (7 mL) was stirred at rt overnight. The precipitate was filtered and washed with cold EtOH (1–2 mL). Colorless crystals, yield 260 mg (66%), mp 174-175 °C. ^{13}C-^{1}H HMBC spectrum was used for signals assignment.

^{1}H NMR (400 MHz, DMSO-d_6): δ = 2.90 (dd, J = 16.5, 9.6 Hz, 1 H, H^a); 3.02 (s, 3 H, Me); 3.23 (dd, J = 16.5, 3.6 Hz, 1 H, H^b); 4.53 (dd, J = 9.6, 3.6 Hz, 1 H, SCH); 7.04 (t, J = 7.4 Hz, 1 H, H^p); 7.28-7.32 (m, 2 H, H^m); 7.54 (d, J = 7.7 Hz, 2 H, H^o); 9.16 (s, 1 H, =NH); 10.11 (s, 1 H, PhN$\underline{H}$) ppm.

^{13}C NMR (100 MHz, DMSO-d_6): δ = 28.0 (CH_3); 39.1 (CH_2); 44.4 (SCH); 119.1 (C^o); 123.4 (C^p); 128.7 (C^m); 138.7 (C^i); 157.8 (C=NH); 167.8 (PhNHC=O); 173.8 (C=O) ppm.

HRMS (ESI), *m/z*: calcd for $C_{12}H_{13}N_3O_2S$ $[M+Na]^+$ 286.0621, found 286.0633.

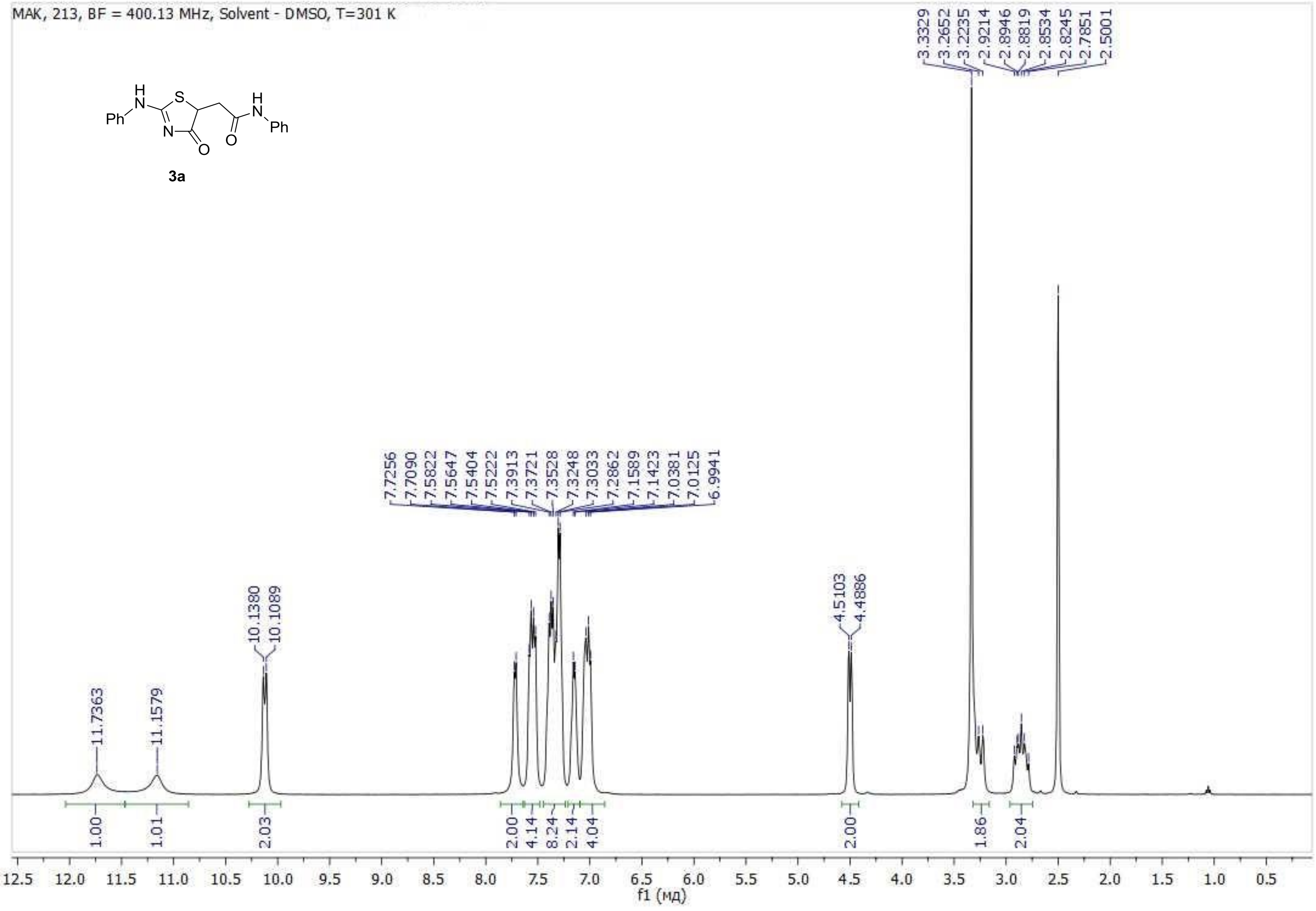

MAK, 213, BF = 400.13 MHz, Solvent - DMSO, T=301 K
3.3329
3.2652
3.2235
2.9214
2.8946
2.8819
2.8534
2.8245
2.7851
2.5001
7.7256
7.7090
7.5822
7.5647
7.5404
7.5222
7.3913
7.3721
7.3528
7.3248
7.3033
7.2862
7.1589
7.1423
7.0381
7.0125
6.9941
4.5103
4.4886
10.1380
10.1089
11.7363
11.1579
1.00
1.01
2.03
2.00
4.14
8.24
2.14
4.04
2.00
1.86
2.04
f1 (мд)

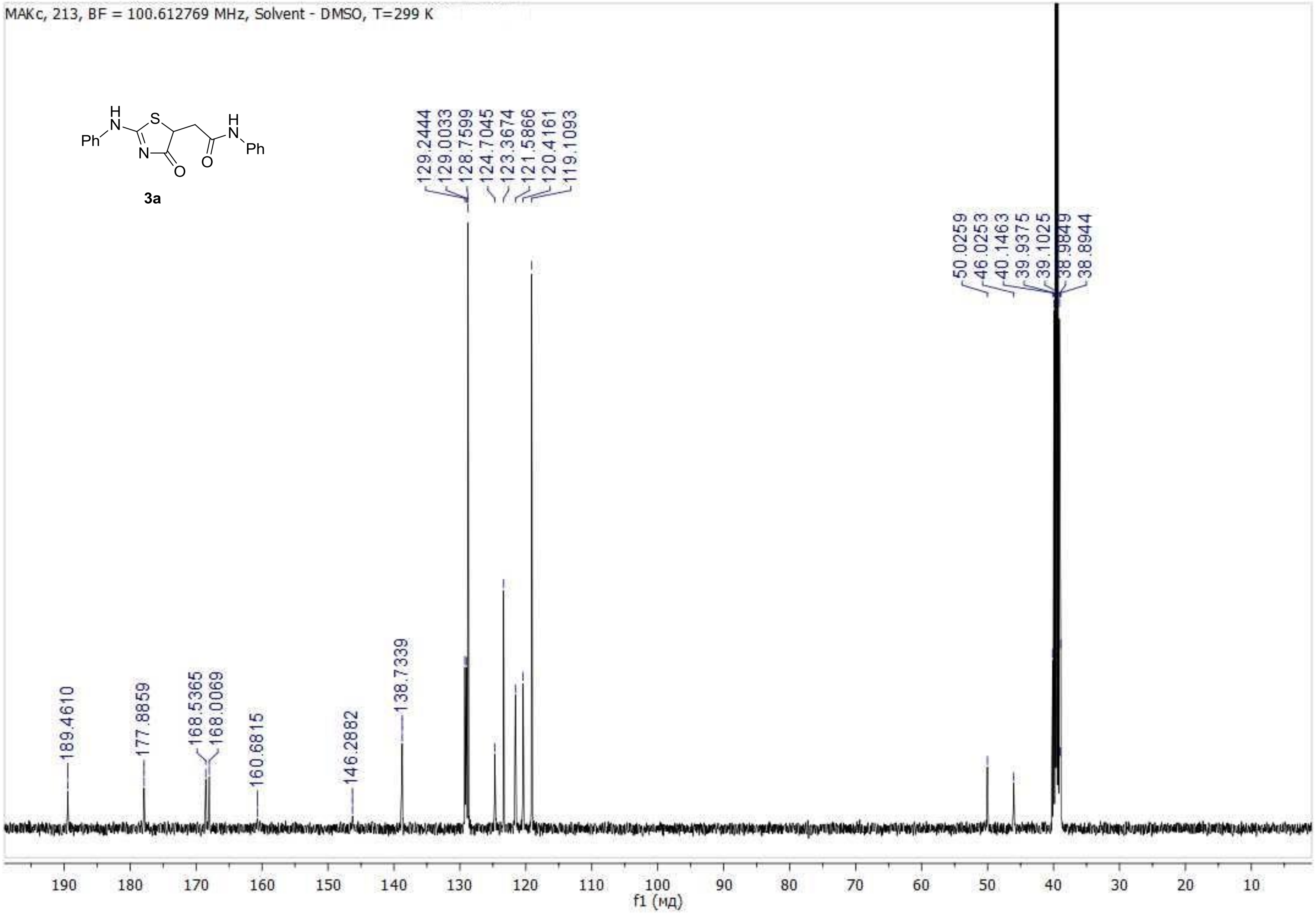
MAKc, 213, BF = 100.612769 MHz, Solvent - DMSO, T=299 K
Ph
H
N
S
N
O
H
N
Ph
O
3a
129.2444
129.0033
128.7599
124.7045
123.3674
121.5866
120.4161
119.1093
50.0259
46.0253
40.1463
39.9375
39.1025
38.9849
38.8944
189.4610
177.8859
168.5365
168.0069
160.6815
146.2882
138.7339
190
180
170
160
150
140
130
120
110
100
90
80
70
60
50
40
30
20
10
f1 (мд)

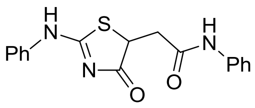

H
N
S
Ph
N
O
H
N
O
Ph

3a

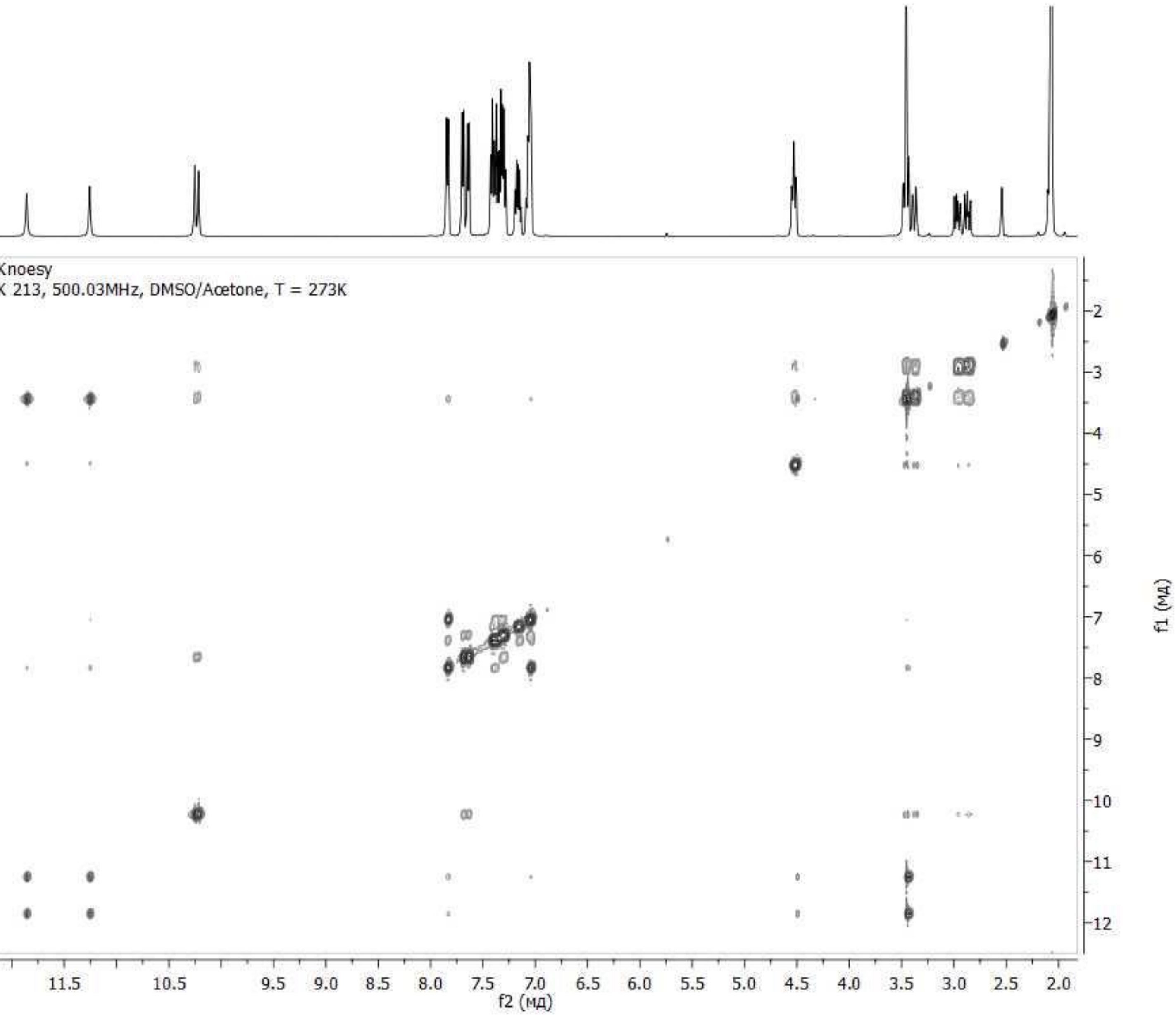

MAKnoesy
MAK 213, 500.03MHz, DMSO/Acetone, T = 273K
f2 (мд)
f1 (мд)

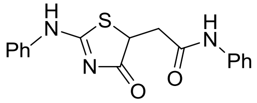

3a

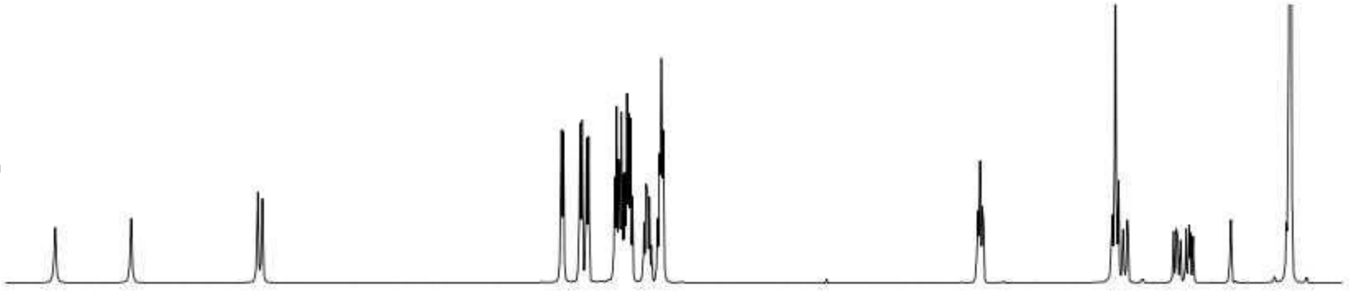

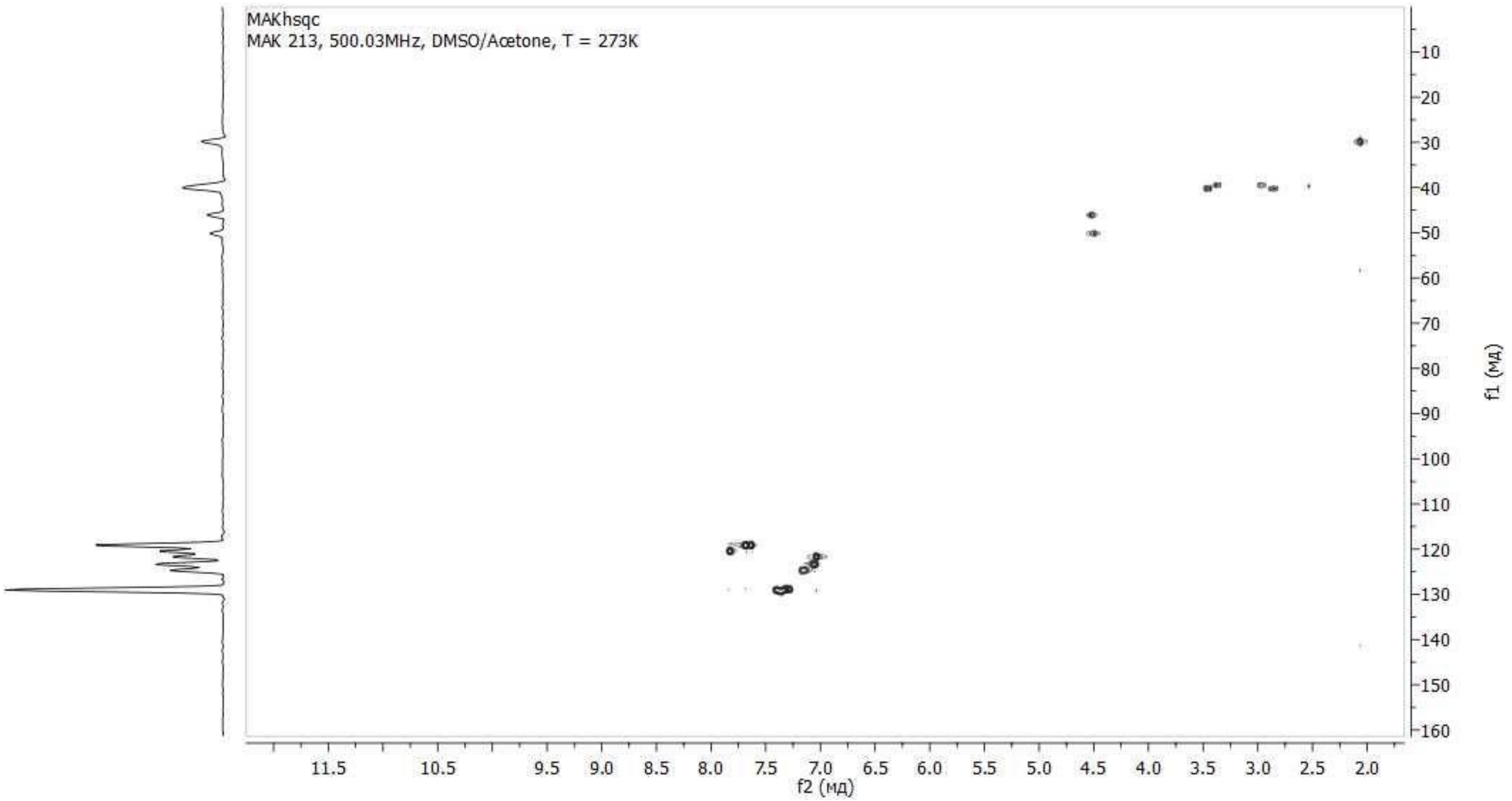

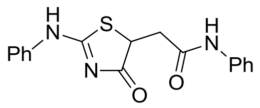

3a

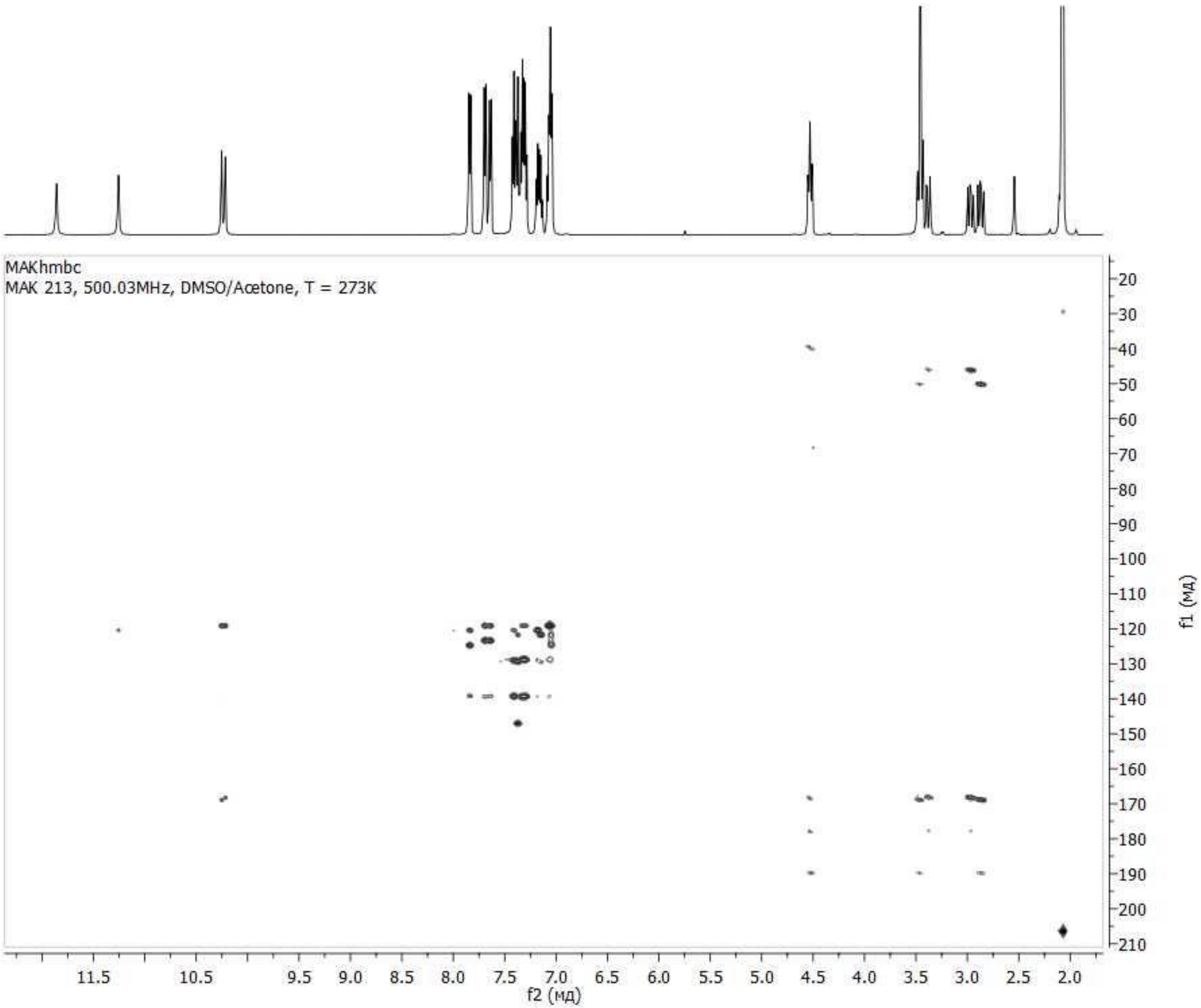

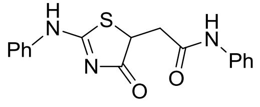

3a

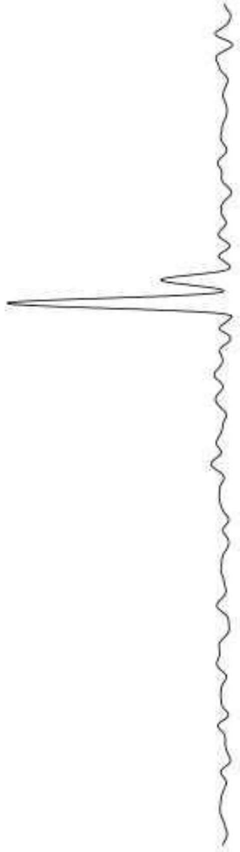

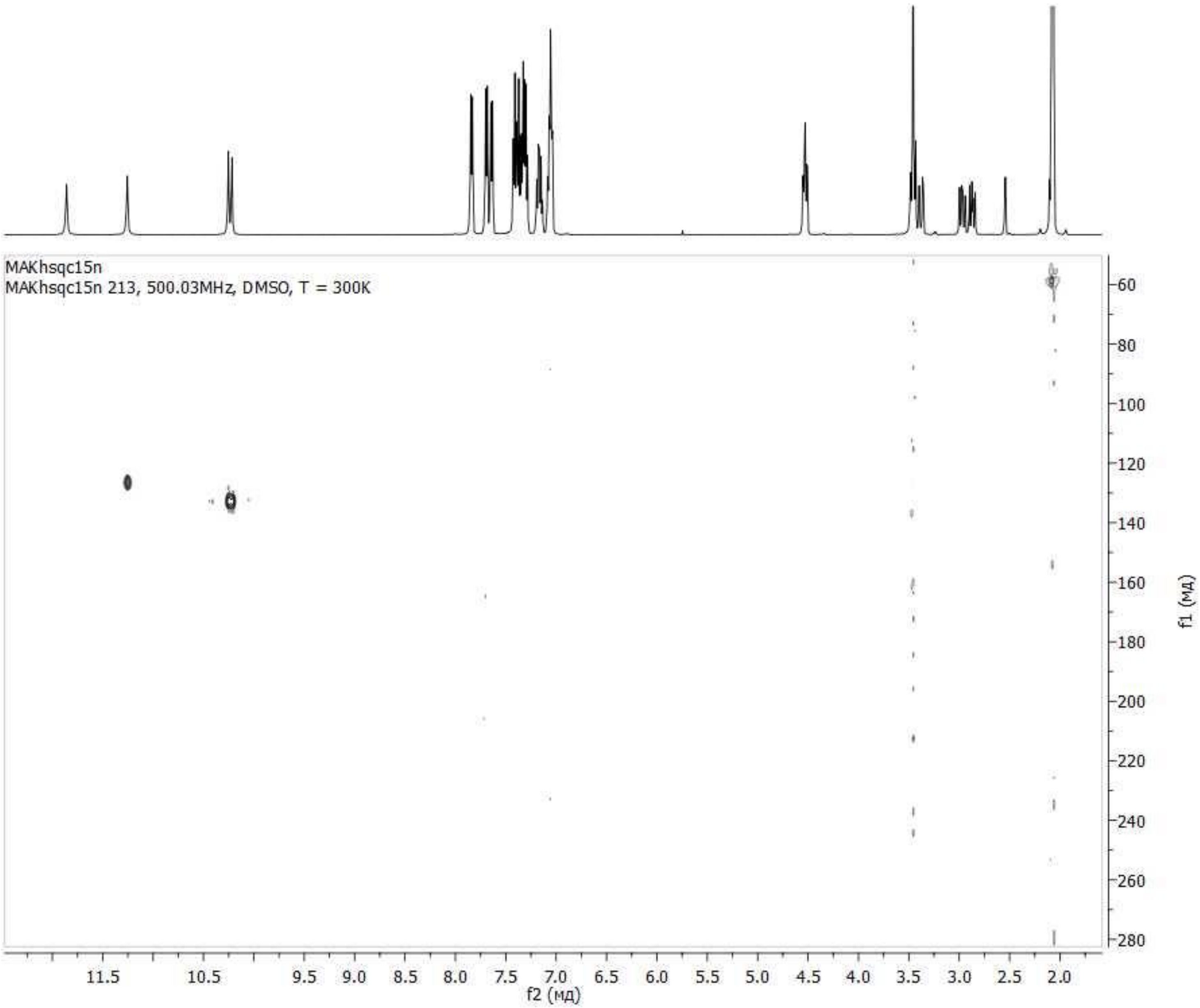

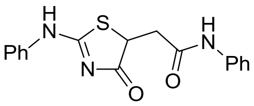

3a

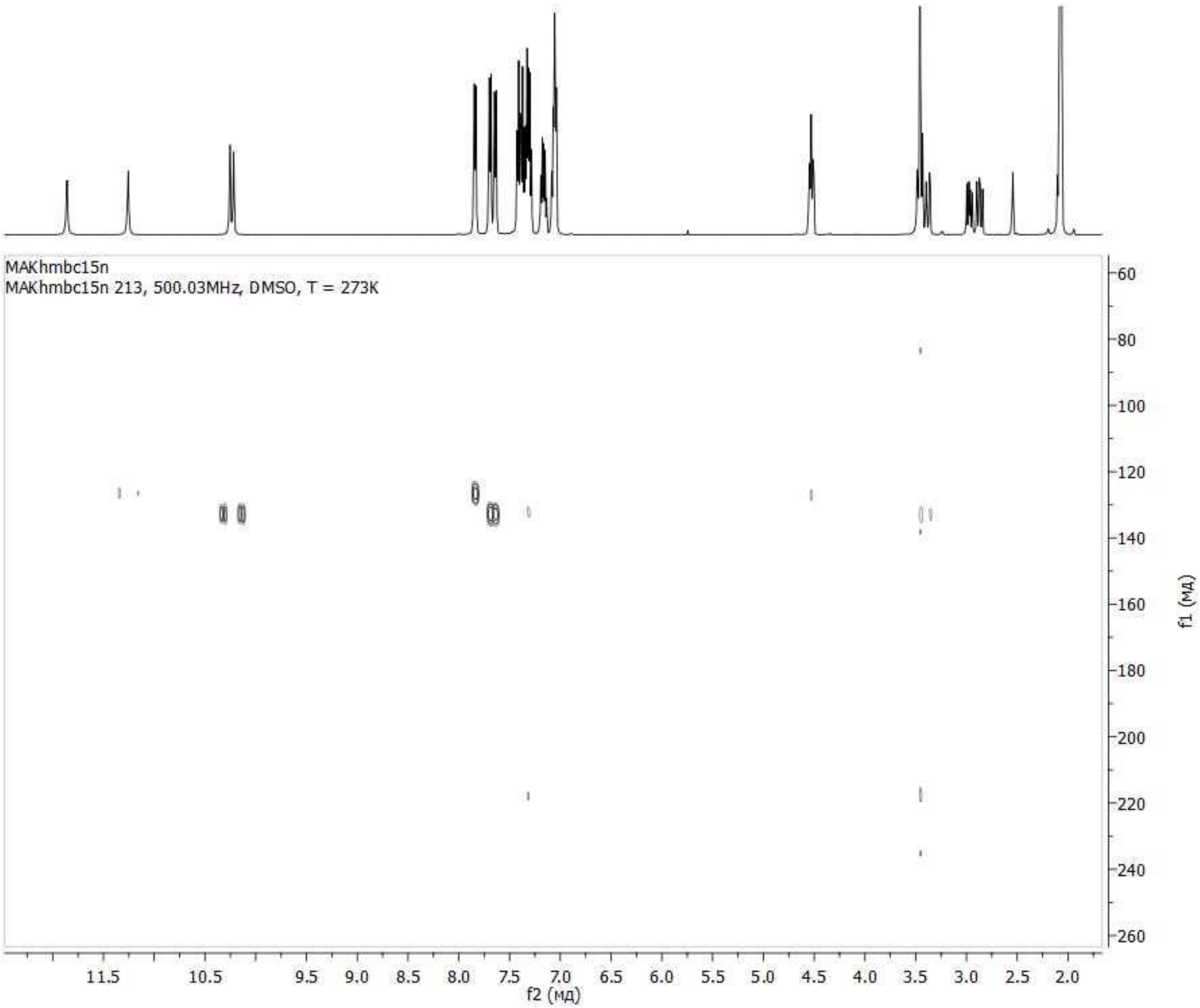

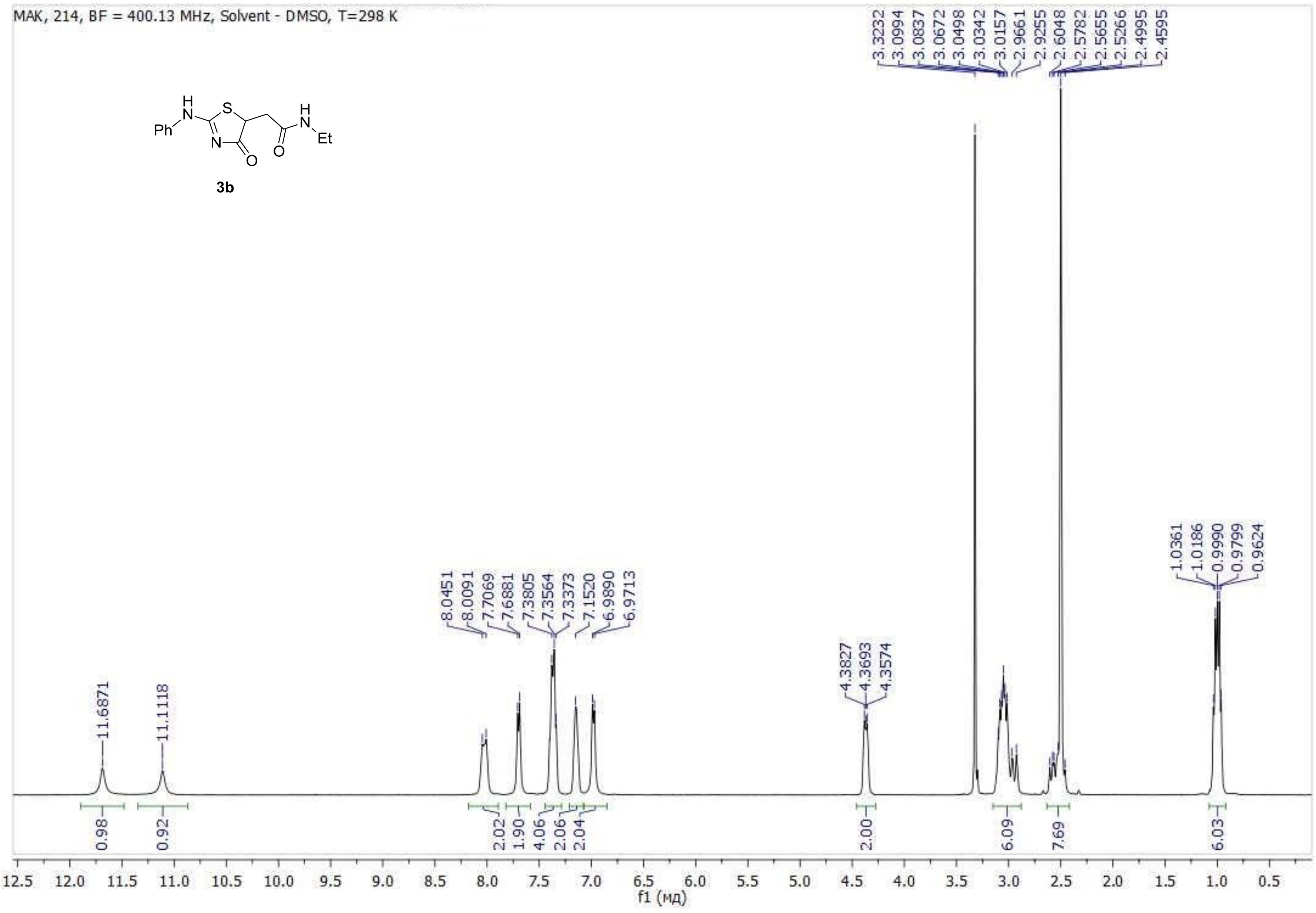
MAK, 214, BF = 400.13 MHz, Solvent - DMSO, T=298 K
3b
11.6871
11.1118
8.0451
8.0091
7.7069
7.6881
7.3805
7.3564
7.3373
7.1520
6.9890
6.9713
4.3827
4.3693
4.3574
3.3232
3.0994
3.0837
3.0672
3.0498
3.0342
3.0157
2.9661
2.9255
2.6048
2.5782
2.5655
2.5266
2.4995
2.4595
1.0361
1.0186
0.9990
0.9799
0.9624
0.98
0.92
2.02
1.90
4.06
2.06
2.04
2.00
6.09
7.69
6.03
f1 (мд)

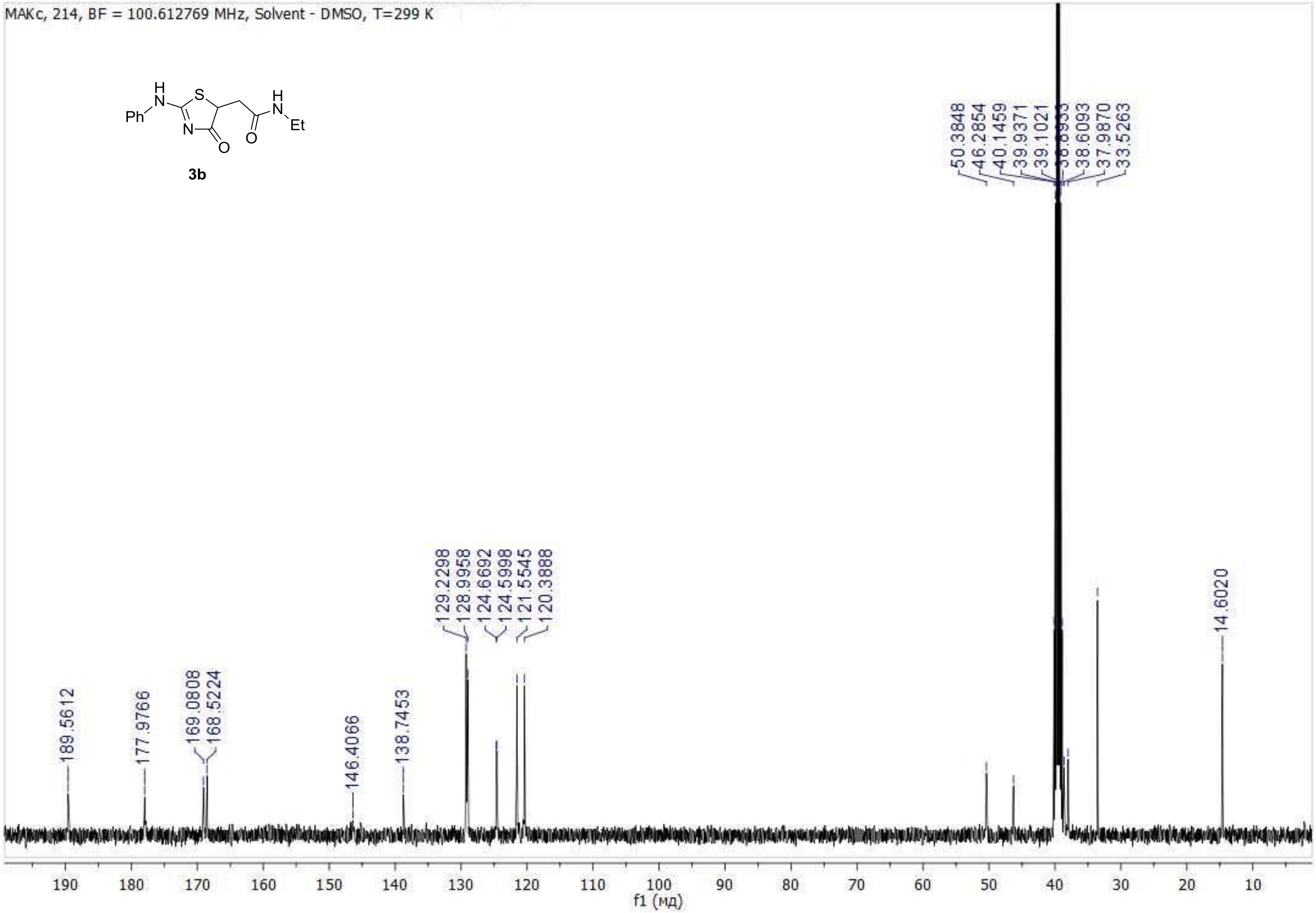
MAKc, 214, BF = 100.612769 MHz, Solvent - DMSO, T=299 K
3b
189.5612
177.9766
169.0808
168.5224
146.4066
138.7453
129.2298
128.9958
124.6692
124.5998
121.5545
120.3888
50.3848
46.2854
40.1459
39.9371
39.1021
38.6093
37.9870
33.5263
14.6020
f1 (мд)

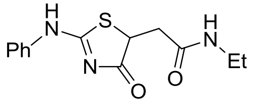
H
N
Ph
S
N
O
H
N
Et
O

3b

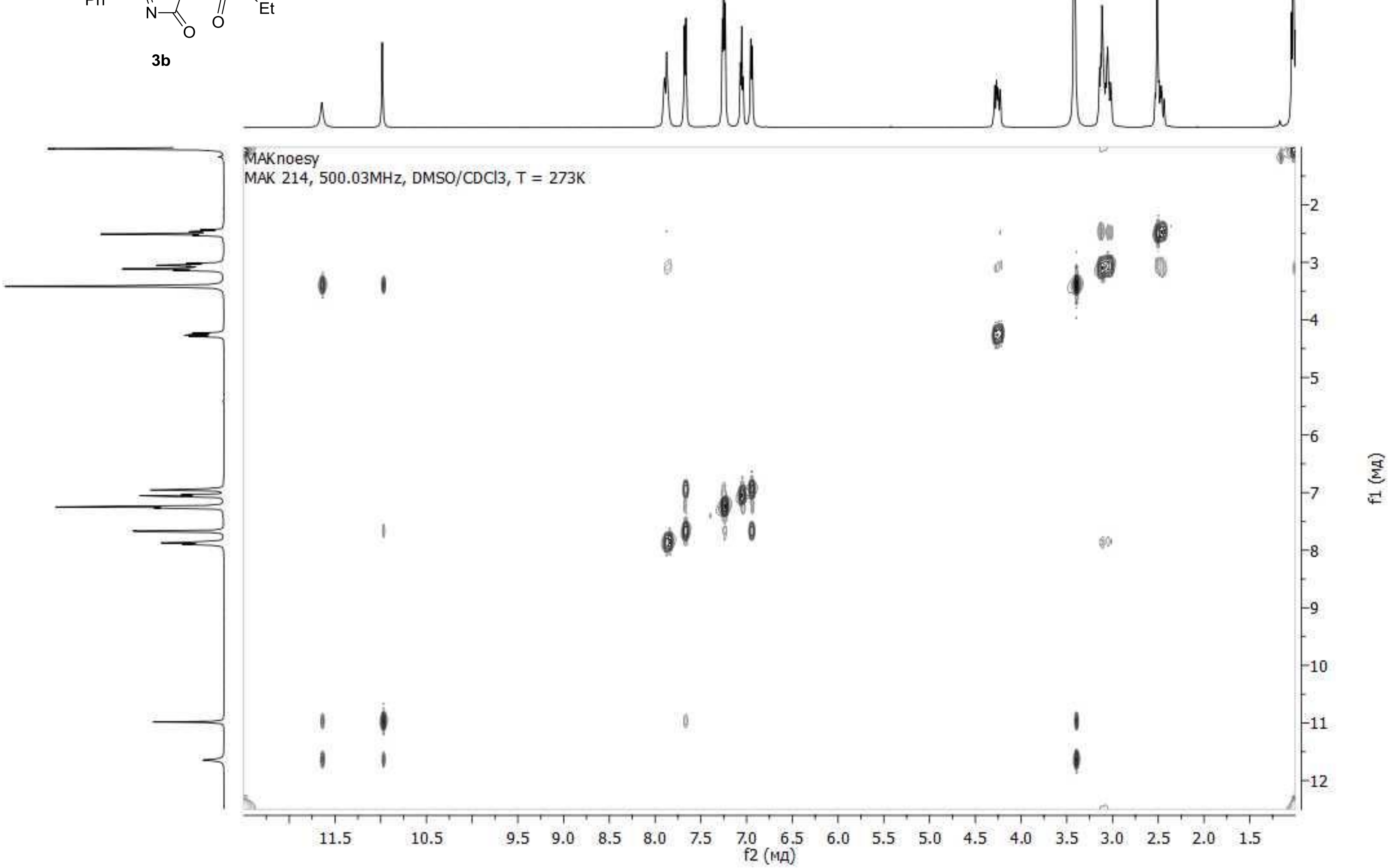
MAKnoesy
MAK 214, 500.03MHz, DMSO/CDCl3, T = 273K
f2 (мд)
f1 (мд)
11.5
10.5
9.5
9.0
8.5
8.0
7.5
7.0
6.5
6.0
5.5
5.0
4.5
4.0
3.5
3.0
2.5
2.0
1.5
2
3
4
5
6
7
8
9
10
11
12

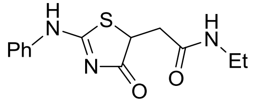

Ph
H
N
S
N
O
O
H
N
Et

3b

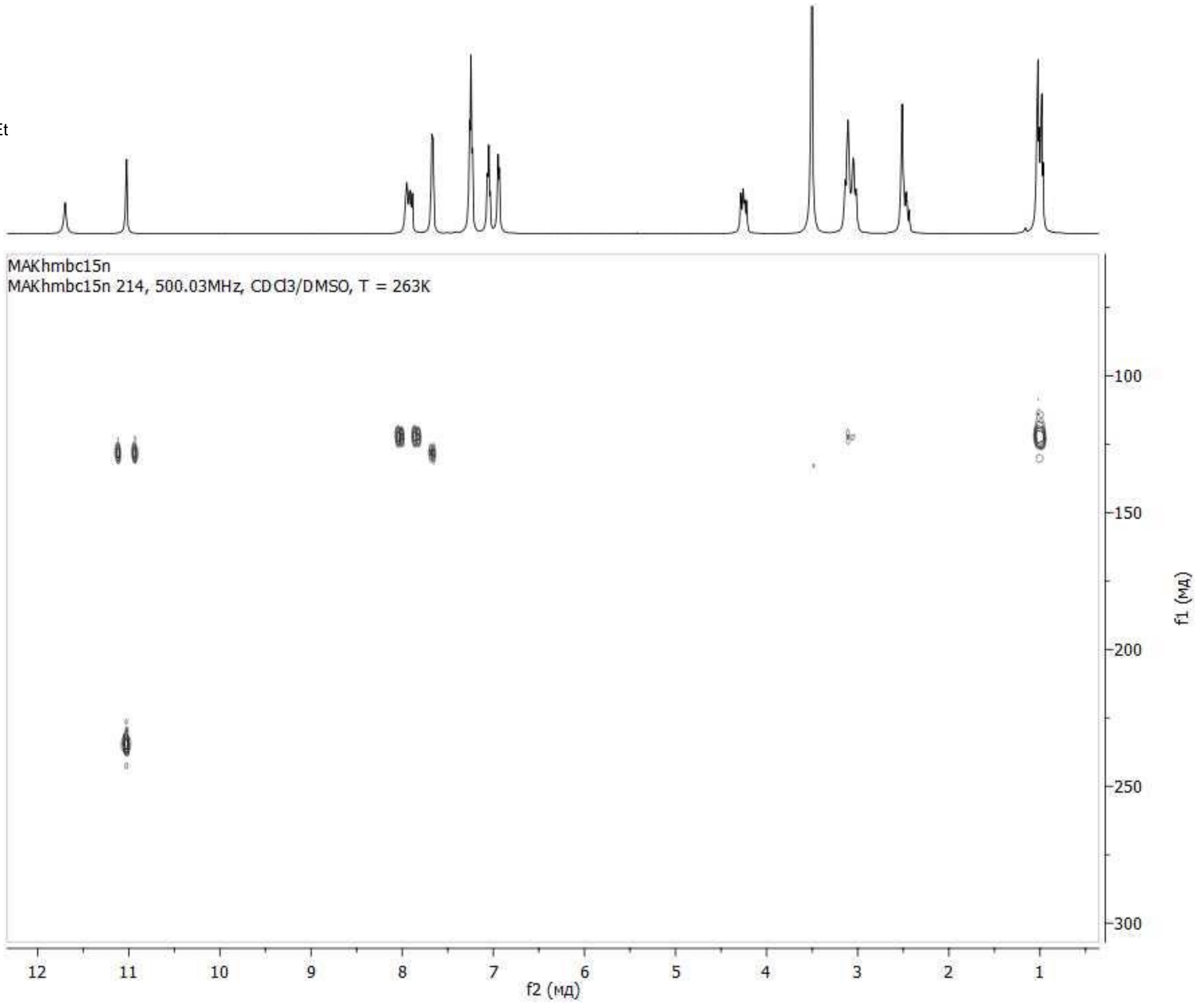

MAKhmbc15n
MAKhmbc15n 214, 500.03MHz, CDCl3/DMSO, T = 263K
f1 (мд)
f2 (мд)

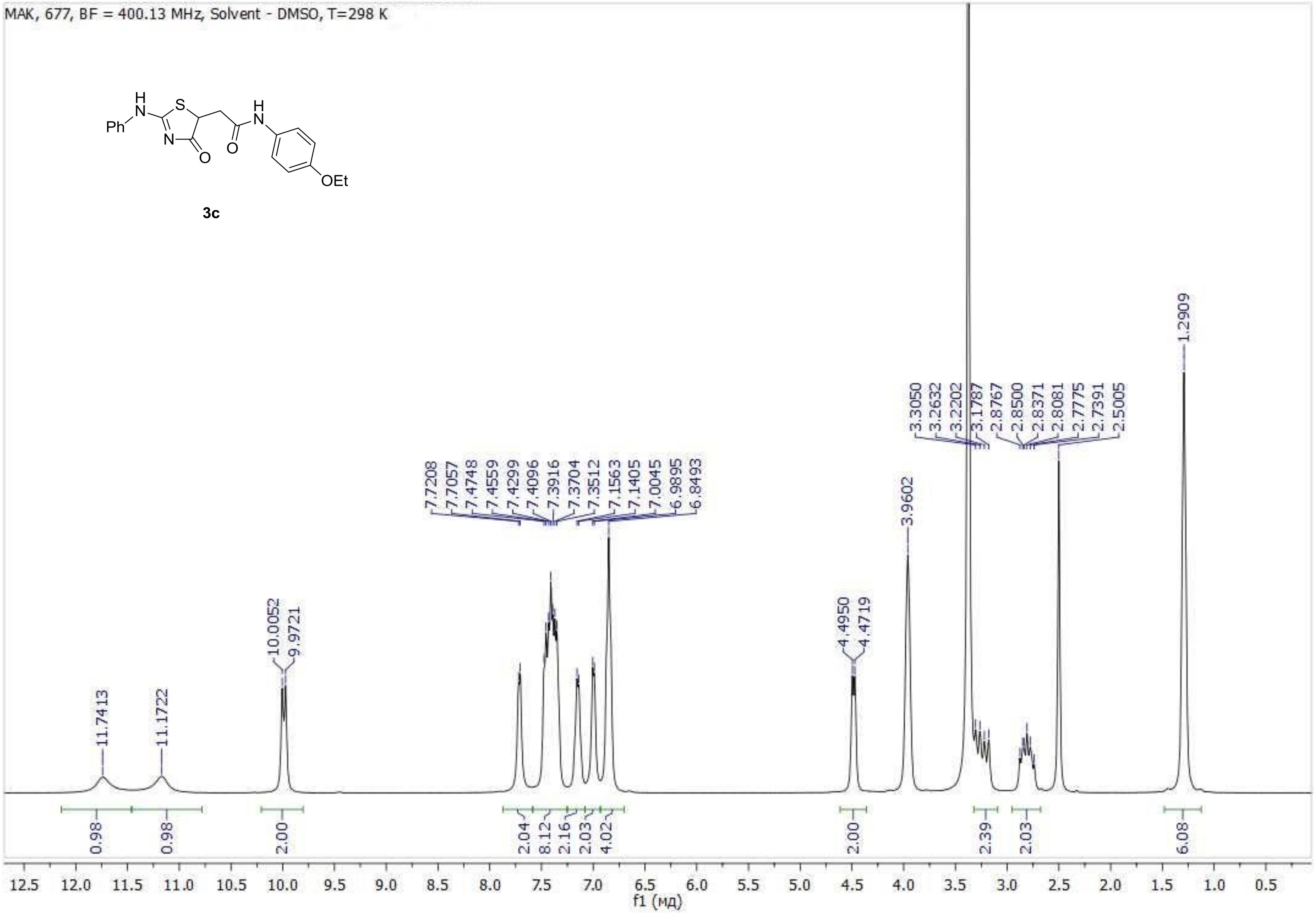
MAK, 677, BF = 400.13 MHz, Solvent - DMSO, T=298 K
H
N
Ph
S
N
O
O
H
N
OEt
3c
11.7413
11.1722
10.0052
9.9721
7.7208
7.7057
7.4748
7.4559
7.4299
7.4096
7.3916
7.3704
7.3512
7.1563
7.1405
7.0045
6.9895
6.8493
4.4950
4.4719
3.9602
3.3050
3.2632
3.2202
3.1787
2.8767
2.8500
2.8371
2.8081
2.7775
2.7391
2.5005
1.2909
0.98
0.98
2.00
2.04
8.12
2.16
2.03
4.02
2.00
2.39
2.03
6.08
12.5 12.0 11.5 11.0 10.5 10.0 9.5 9.0 8.5 8.0 7.5 7.0 6.5 6.0 5.5 5.0 4.5 4.0 3.5 3.0 2.5 2.0 1.5 1.0 0.5
f1 (мд)

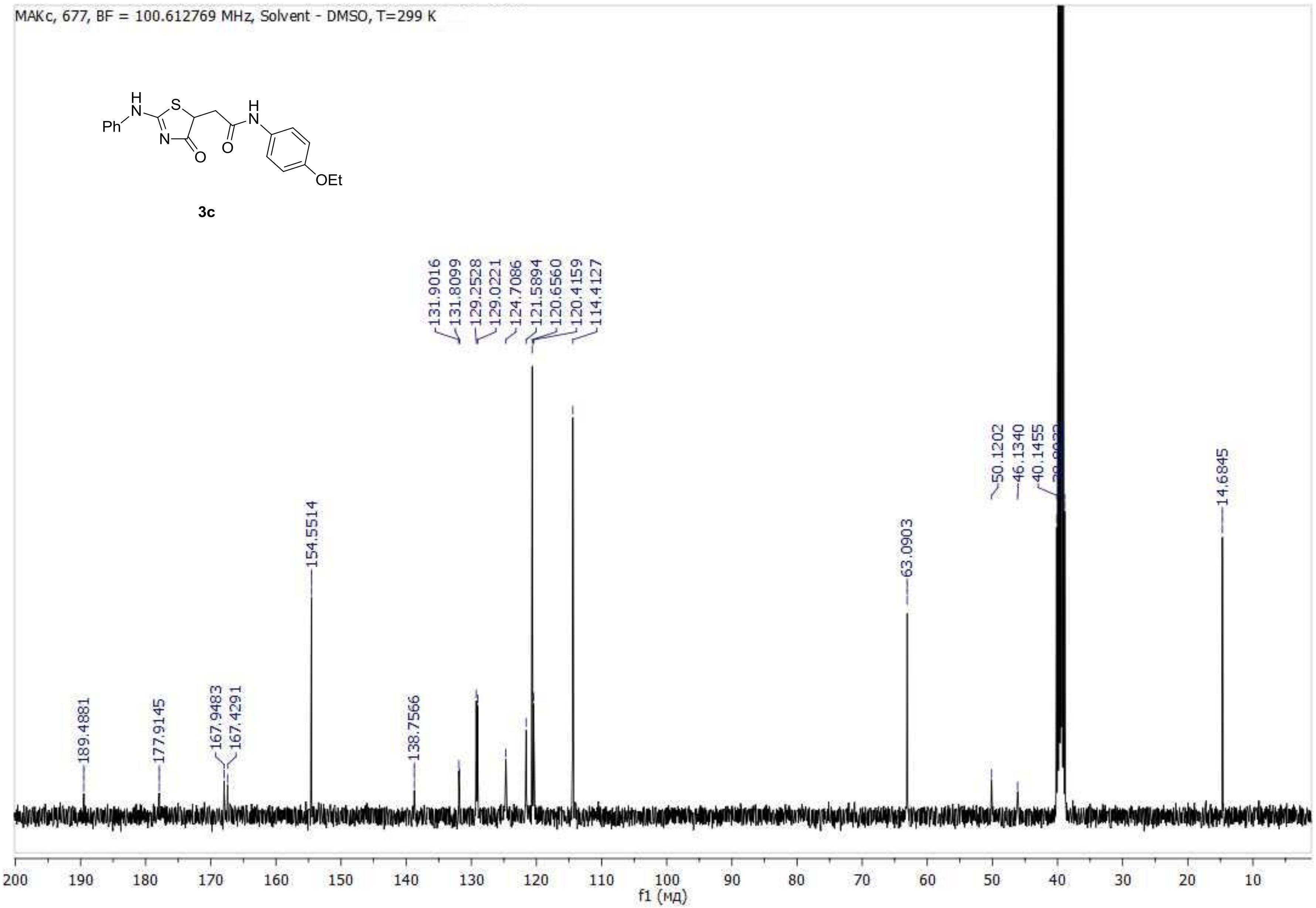

MAKc, 677, BF = 100.612769 MHz, Solvent - DMSO, T=299 K
3c
189.4881
177.9145
167.9483
167.4291
154.5514
138.7566
131.9016
131.8099
129.2528
129.0221
124.7086
121.5894
120.6560
120.4159
114.4127
63.0903
50.1202
46.1340
40.1455
14.6845
f1 (мд)

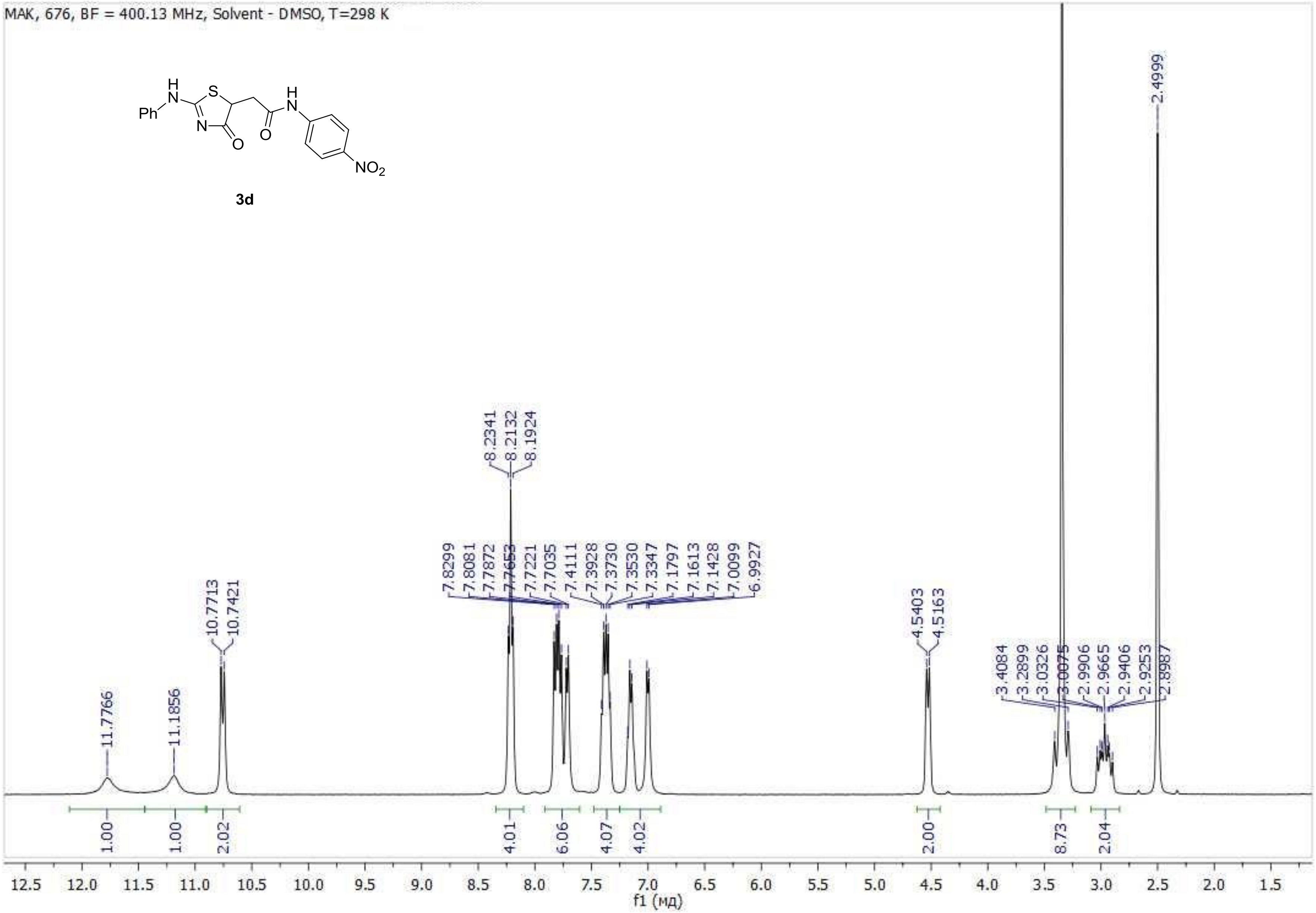
MAK, 676, BF = 400.13 MHz, Solvent - DMSO, T=298 K
3d
11.7766
11.1856
10.7713
10.7421
8.2341
8.2132
8.1924
7.8299
7.8081
7.7872
7.7653
7.7221
7.7035
7.4111
7.3928
7.3730
7.3530
7.3347
7.1797
7.1613
7.1428
7.0099
6.9927
4.5403
4.5163
3.4084
3.2899
3.0326
3.0075
2.9906
2.9665
2.9406
2.9253
2.8987
2.4999
1.00
1.00
2.02
4.01
6.06
4.07
4.02
2.00
8.73
2.04
f1 (мд)

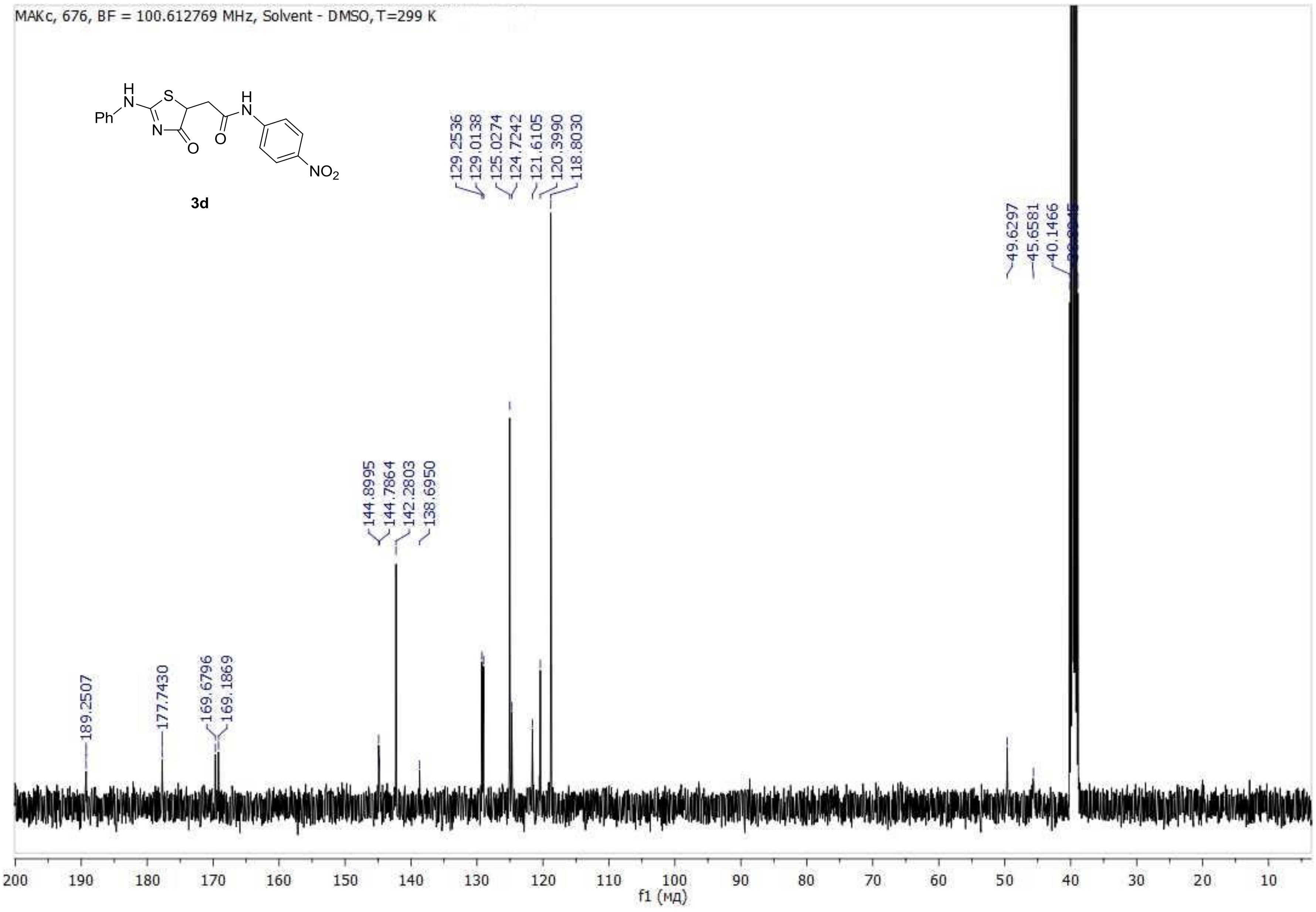

MAKc, 676, BF = 100.612769 MHz, Solvent - DMSO, T=299 K
Ph
H
N
S
N
O
O
H
N
NO2
3d
189.2507
177.7430
169.6796
169.1869
144.8995
144.7864
142.2803
138.6950
129.2536
129.0138
125.0274
124.7242
121.6105
120.3990
118.8030
49.6297
45.6581
40.1466
200 190 180 170 160 150 140 130 120 110 100 90 80 70 60 50 40 30 20 10
f1 (мд)

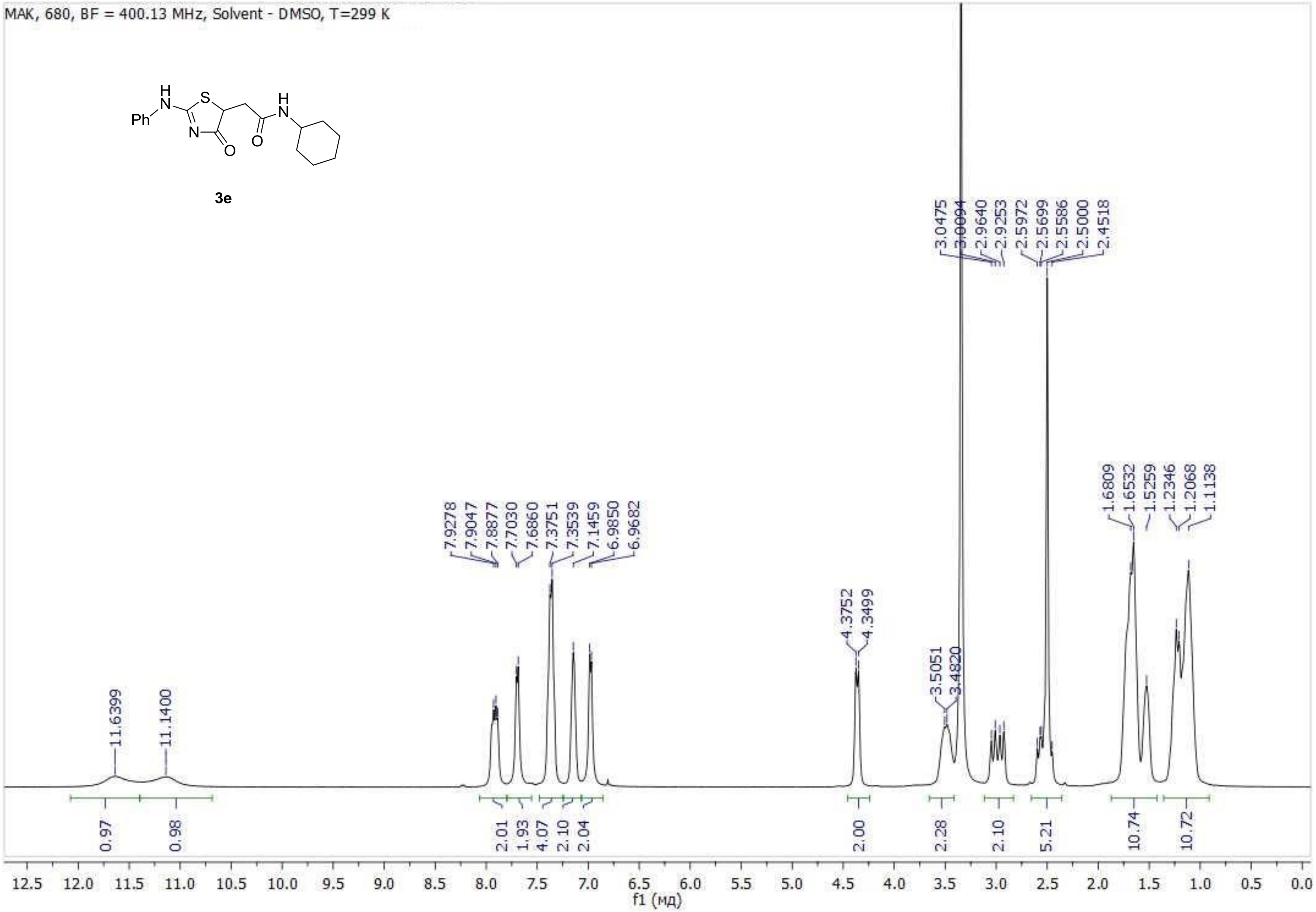
MAK, 680, BF = 400.13 MHz, Solvent - DMSO, T=299 K
3e
11.6399
11.1400
7.9278
7.9047
7.8877
7.7030
7.6860
7.3751
7.3539
7.1459
6.9850
6.9682
4.3752
4.3499
3.5051
3.4820
3.0475
3.0094
2.9640
2.9253
2.5972
2.5699
2.5586
2.5000
2.4518
1.6809
1.6532
1.5259
1.2346
1.2068
1.1138
0.97
0.98
2.01
1.93
4.07
2.10
2.04
2.00
2.28
2.10
5.21
10.74
10.72
f1 (мд)

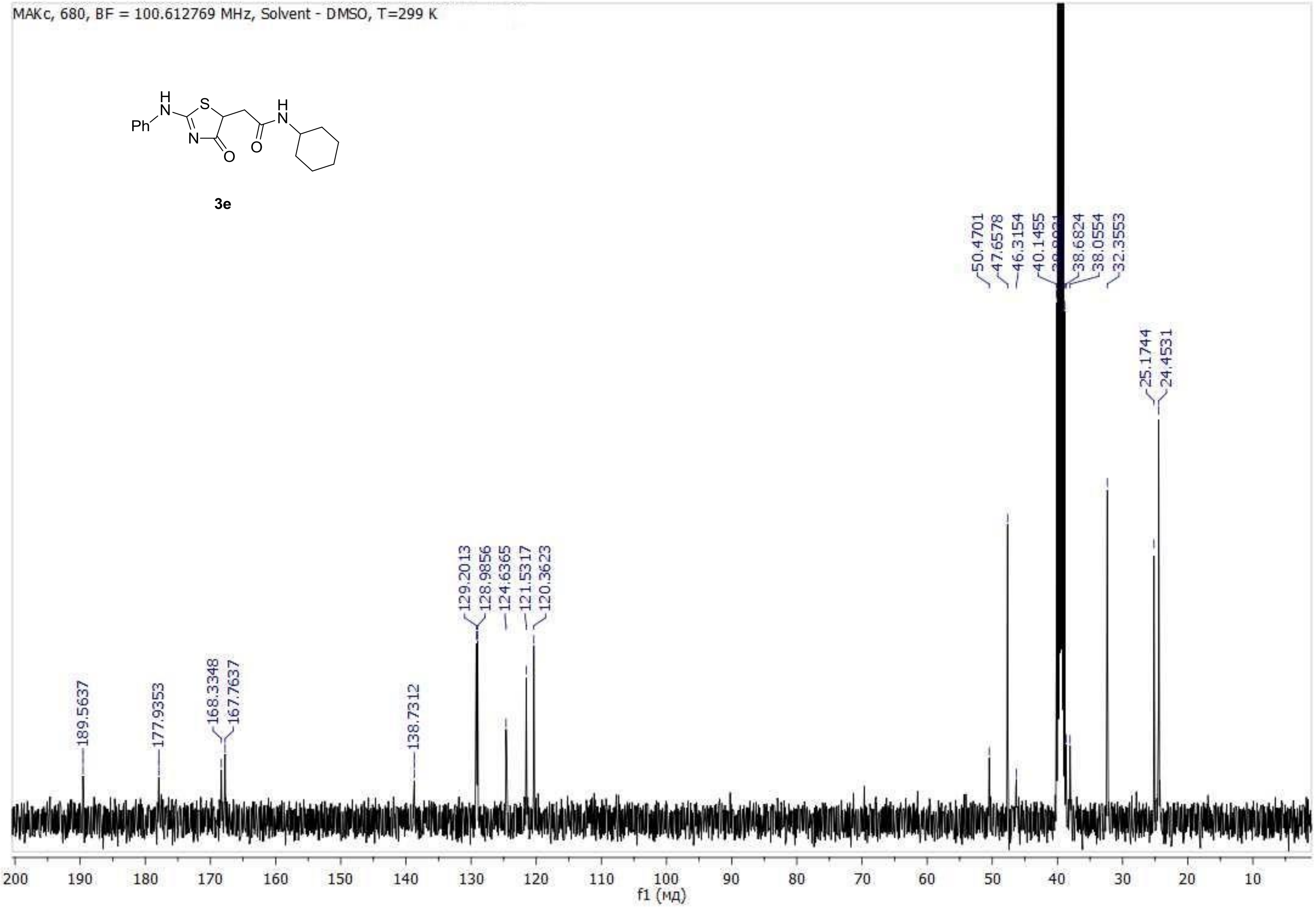
MAKc, 680, BF = 100.612769 MHz, Solvent - DMSO, T=299 K
Ph
H
N
S
N
O
H
N
O
3e
189.5637
177.9353
168.3348
167.7637
138.7312
129.2013
128.9856
124.6365
121.5317
120.3623
50.4701
47.6578
46.3154
40.1455
38.6824
38.0554
32.3553
25.1744
24.4531
200 190 180 170 160 150 140 130 120 110 100 90 80 70 60 50 40 30 20 10
f1 (мд)

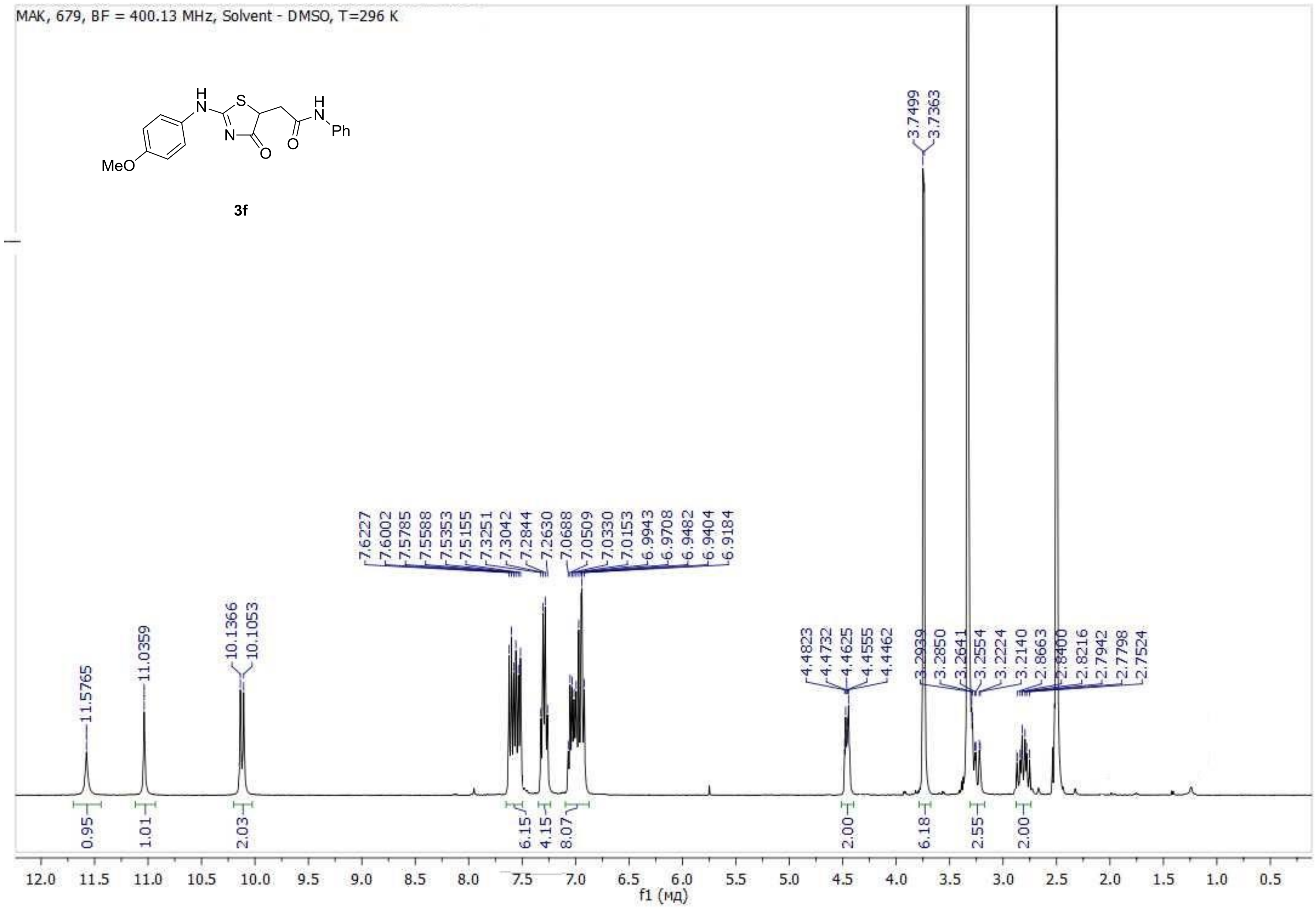
MAK, 679, BF = 400.13 MHz, Solvent - DMSO, T=296 K
MeO
N
S
H
N
O
O
Ph
3f
11.5765
11.0359
10.1366
10.1053
7.6227
7.6002
7.5785
7.5588
7.5353
7.5155
7.3251
7.3042
7.2844
7.2630
7.0688
7.0509
7.0330
7.0153
6.9943
6.9708
6.9482
6.9404
6.9184
4.4823
4.4732
4.4625
4.4555
4.4462
3.7499
3.7363
3.2939
3.2850
3.2641
3.2554
3.2224
3.2140
2.8663
2.8400
2.8216
2.7942
2.7798
2.7524
0.95
1.01
2.03
6.15
4.15
8.07
2.00
6.18
2.55
2.00
f1 (мд)

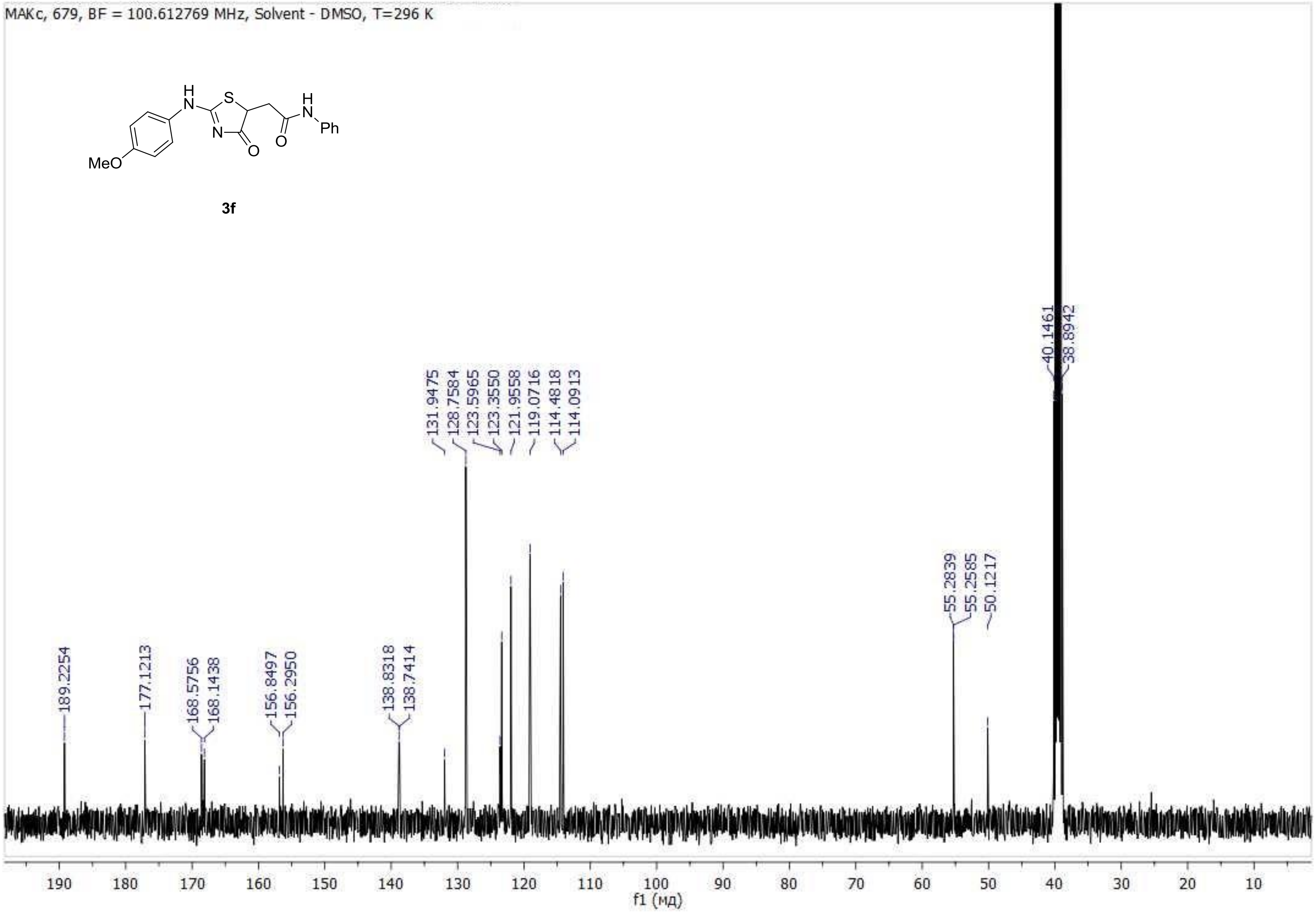

MAKc, 679, BF = 100.612769 MHz, Solvent - DMSO, T=296 K
MeO
N
H
S
N
O
O
H
N
Ph
3f
189.2254
177.1213
168.5756
168.1438
156.8497
156.2950
138.8318
138.7414
131.9475
128.7584
123.5965
123.3550
121.9558
119.0716
114.4818
114.0913
55.2839
55.2585
50.1217
40.1461
38.8942
f1 (мд)

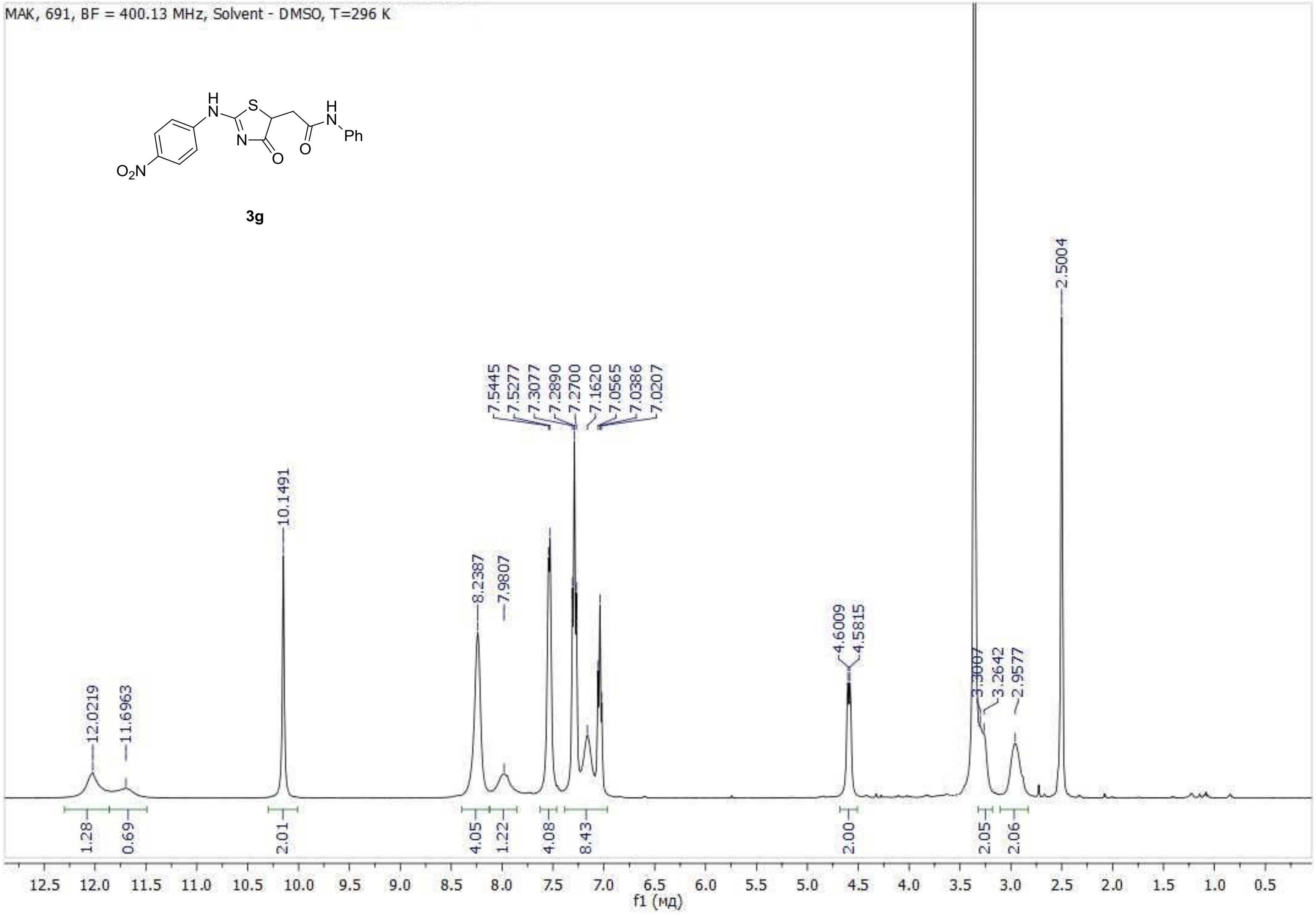

MAK, 691, BF = 400.13 MHz, Solvent - DMSO, T=296 K
3g
12.0219
11.6963
10.1491
8.2387
7.9807
7.5445
7.5277
7.3077
7.2890
7.2700
7.1620
7.0565
7.0386
7.0207
4.6009
4.5815
3.3007
3.2642
2.9577
2.5004
1.28
0.69
2.01
4.05
1.22
4.08
8.43
2.00
2.05
2.06
f1 (мд)

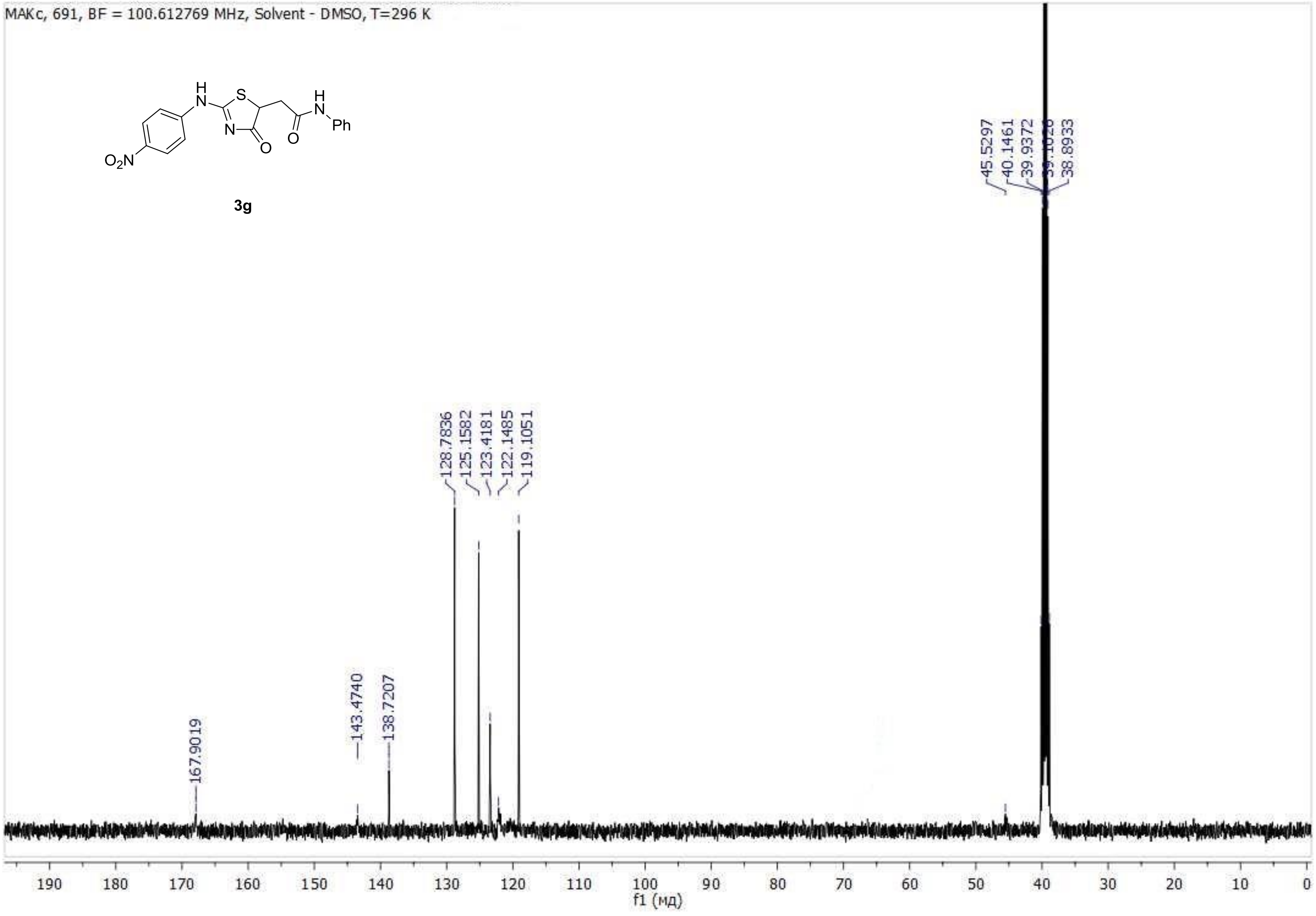
MAKc, 691, BF = 100.612769 MHz, Solvent - DMSO, T=296 K
O2N
3g
167.9019
143.4740
138.7207
128.7836
125.1582
123.4181
122.1485
119.1051
45.5297
40.1461
39.9372
38.8933
f1 (мд)

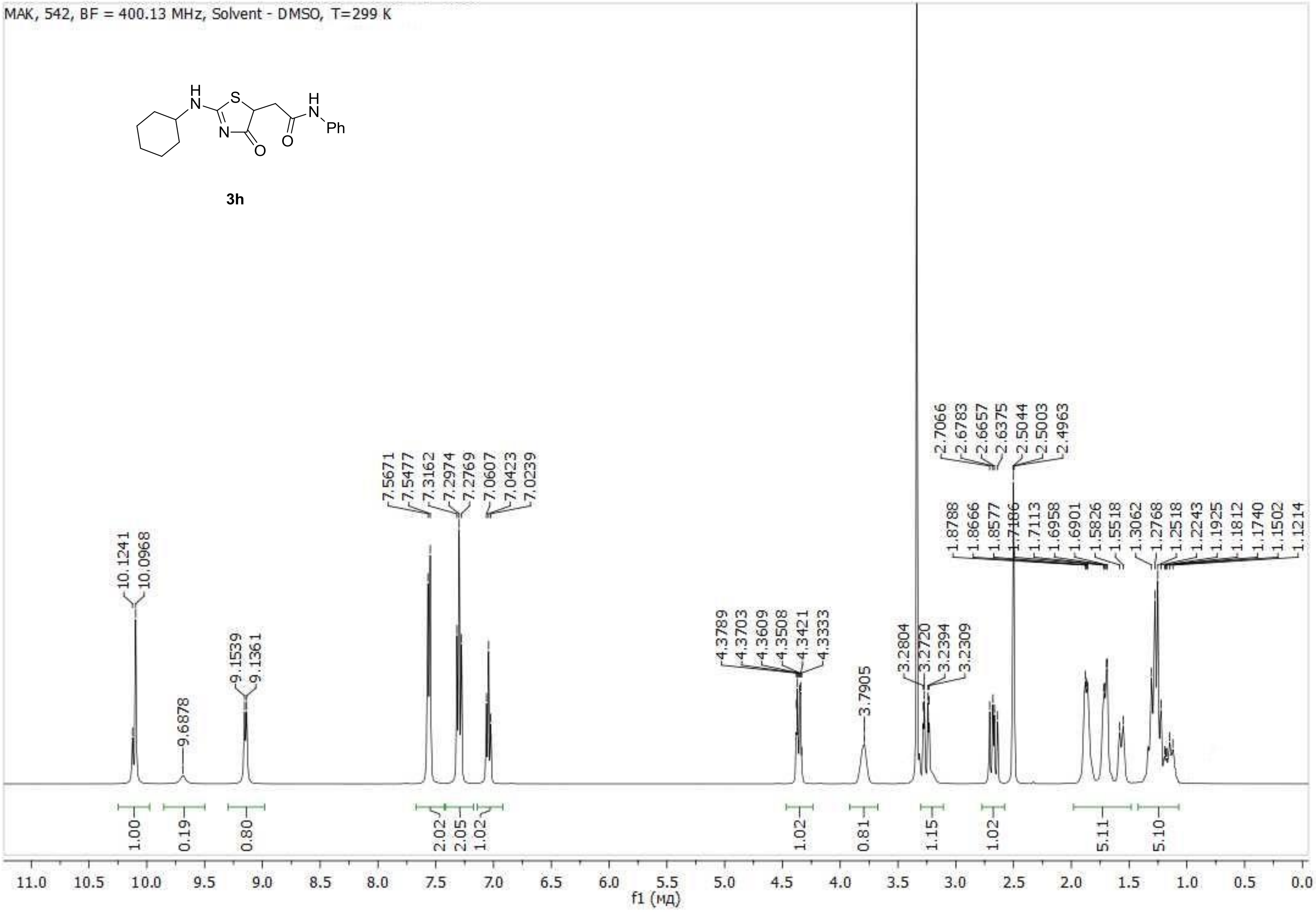
MAK, 542, BF = 400.13 MHz, Solvent - DMSO, T=299 K
3h
f1 (мд)

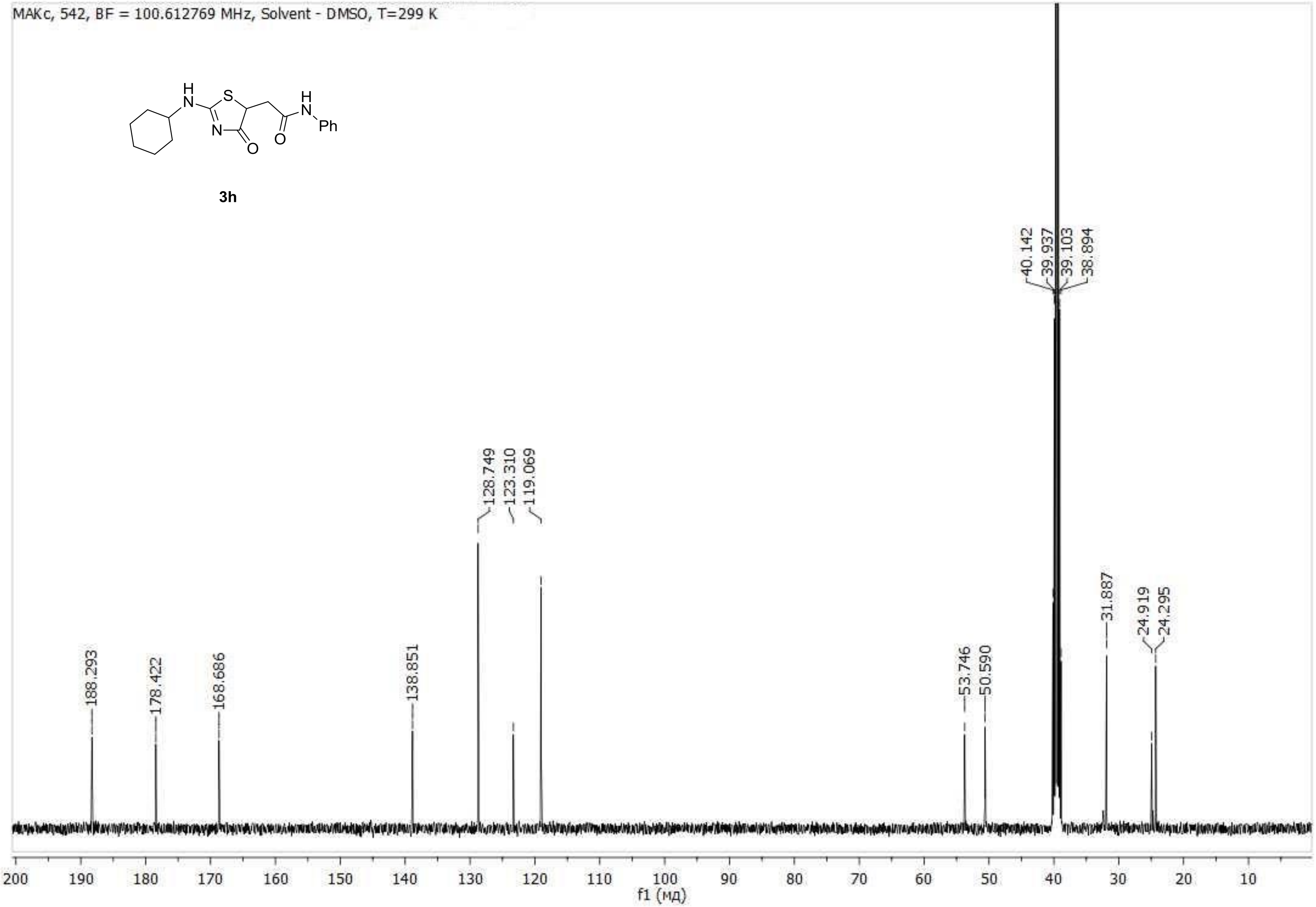
MAKc, 542, BF = 100.612769 MHz, Solvent - DMSO, T=299 K
H
N
S
N
O
H
N
O
Ph
3h
188.293
178.422
168.686
138.851
128.749
123.310
119.069
53.746
50.590
40.142
39.937
39.103
38.894
31.887
24.919
24.295
200
190
180
170
160
150
140
130
120
110
100
90
80
70
60
50
40
30
20
10
f1 (мд)

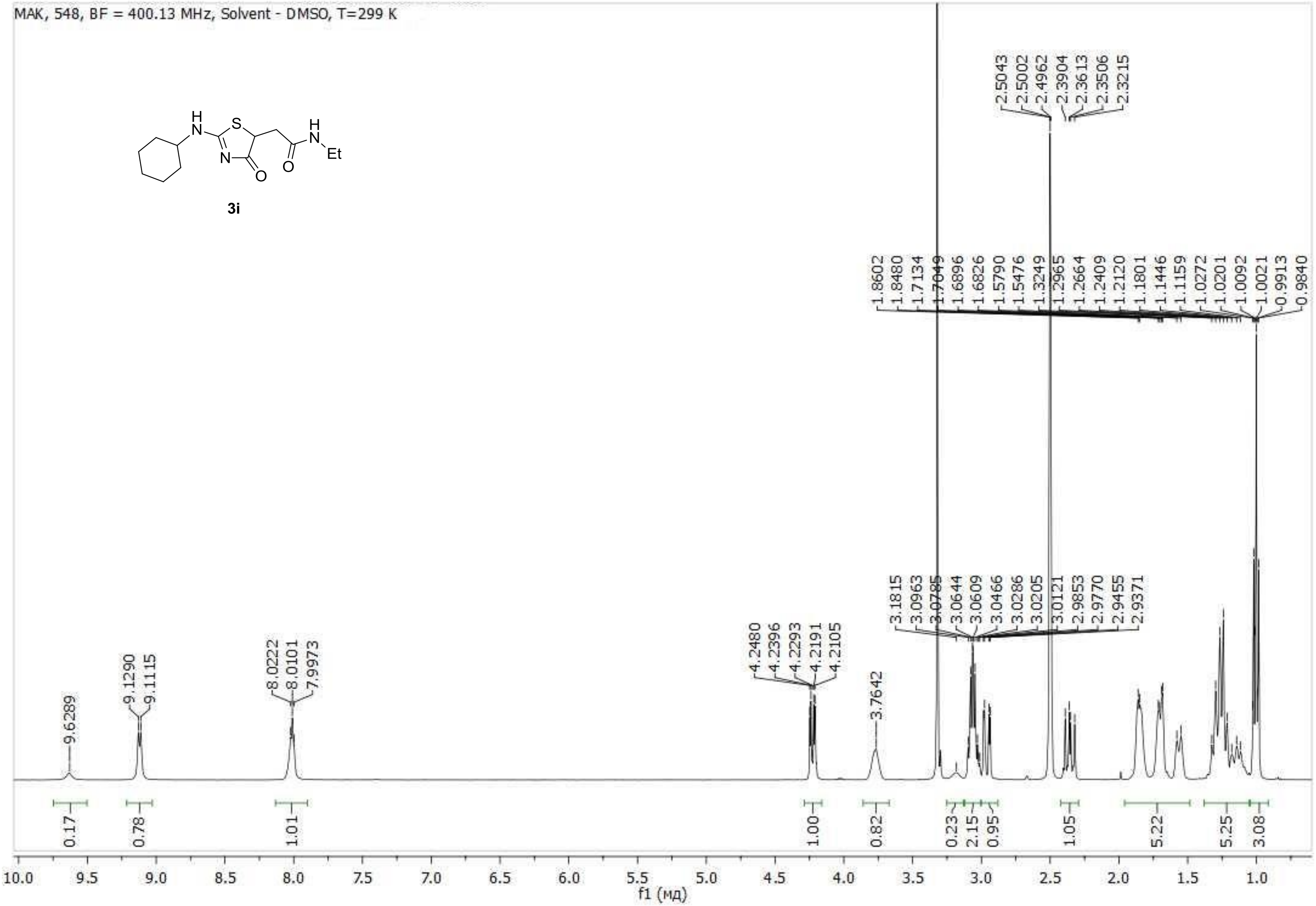

MAK, 548, BF = 400.13 MHz, Solvent - DMSO, T=299 K
3i
f1 (мд)

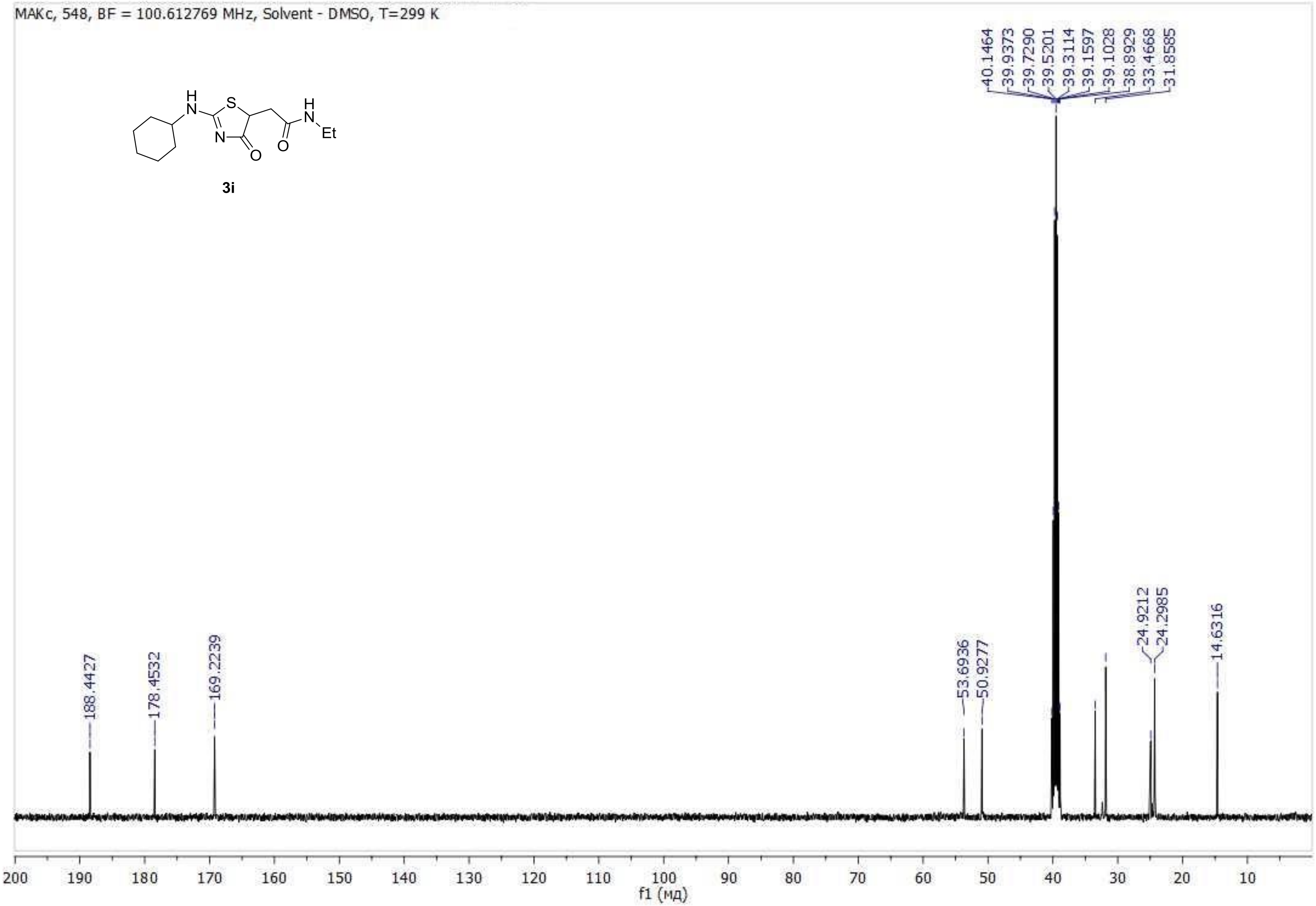
MAKc, 548, BF = 100.612769 MHz, Solvent - DMSO, T=299 K
S
H
N
N
O
H
N
Et
O
3i
40.1464
39.9373
39.7290
39.5201
39.3114
39.1597
39.1028
38.8929
33.4668
31.8585
188.4427
178.4532
169.2239
53.6936
50.9277
24.9212
24.2985
14.6316
200
190
180
170
160
150
140
130
120
110
100
90
80
70
60
50
40
30
20
10
f1 (мд)

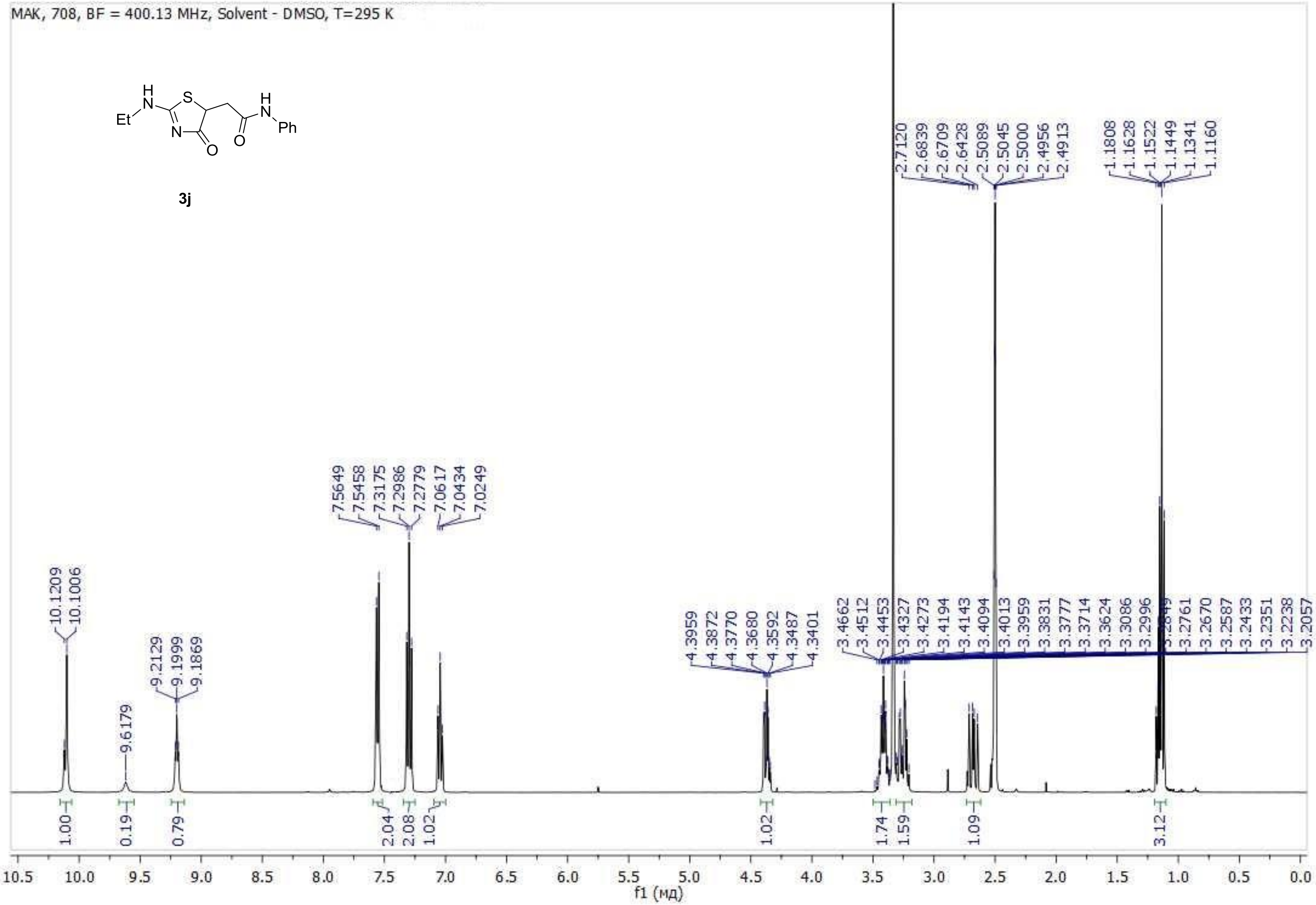
MAK, 708, BF = 400.13 MHz, Solvent - DMSO, T=295 K
3j
f1 (мд)

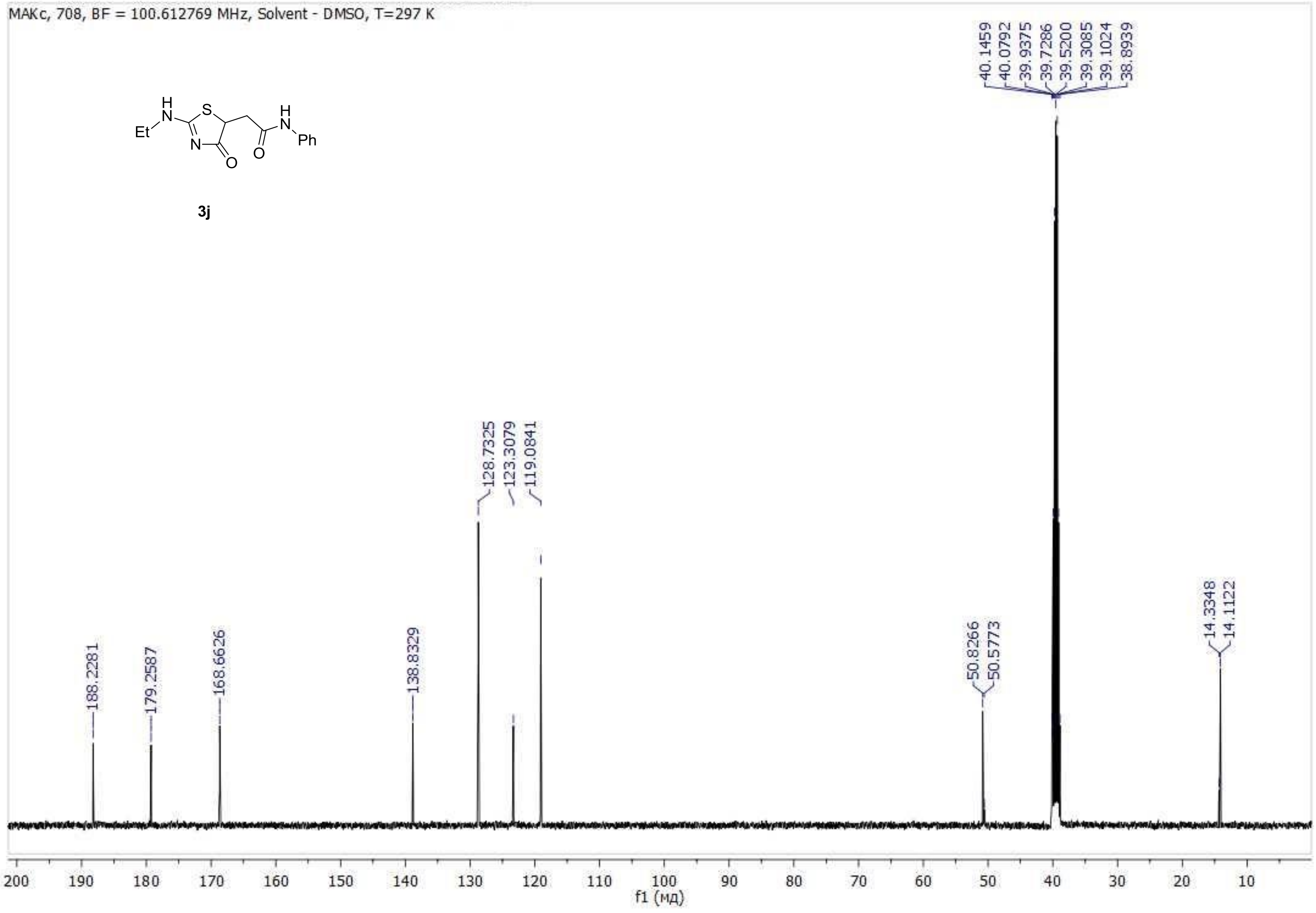
MAKc, 708, BF = 100.612769 MHz, Solvent - DMSO, T=297 K
3j
40.1459
40.0792
39.9375
39.7286
39.5200
39.3085
39.1024
38.8939
128.7325
123.3079
119.0841
188.2281
179.2587
168.6626
138.8329
50.8266
50.5773
14.3348
14.1122
f1 (мд)

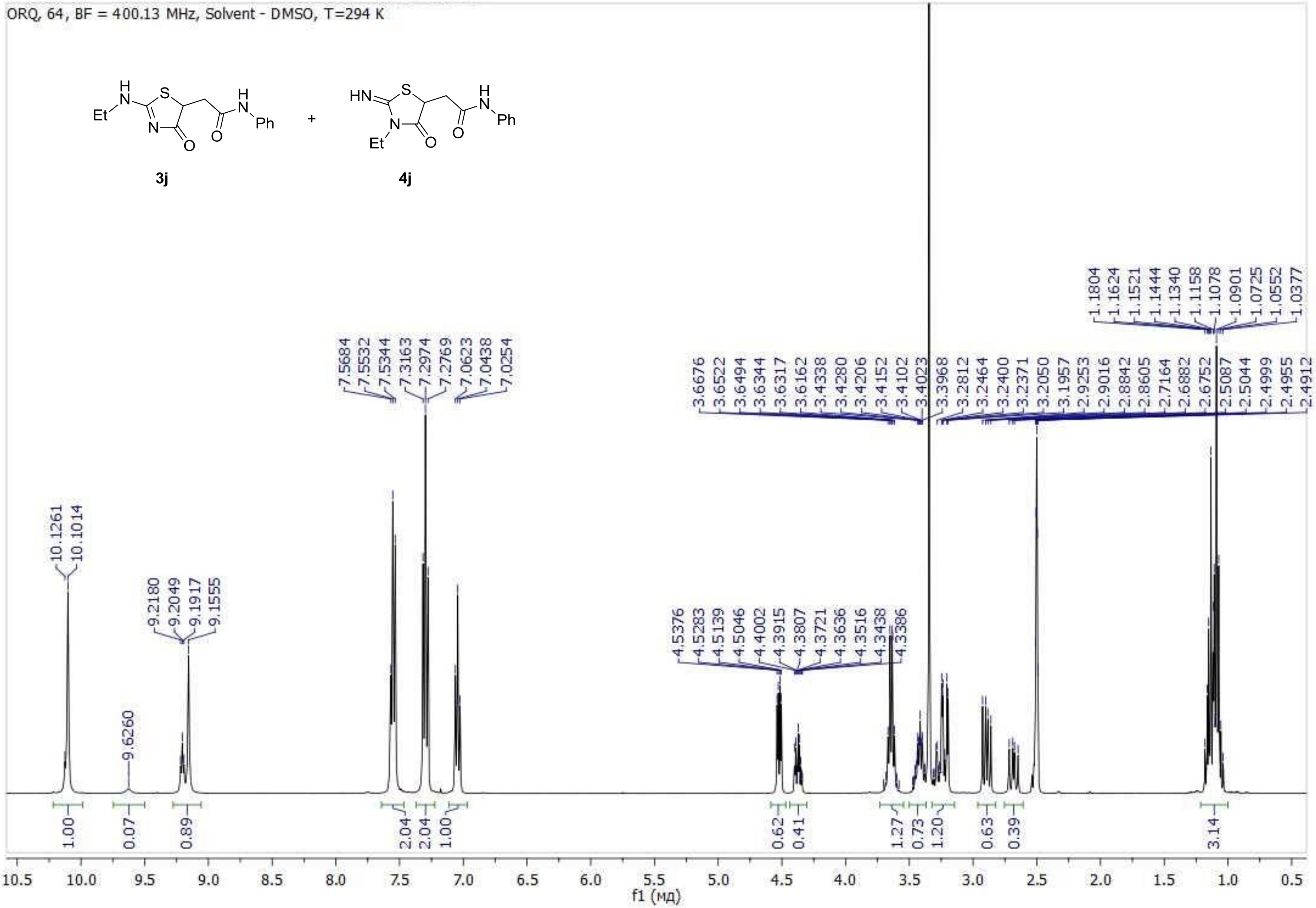
ORQ, 64, BF = 400.13 MHz, Solvent - DMSO, T=294 K
3j
4j
f1 (мд)

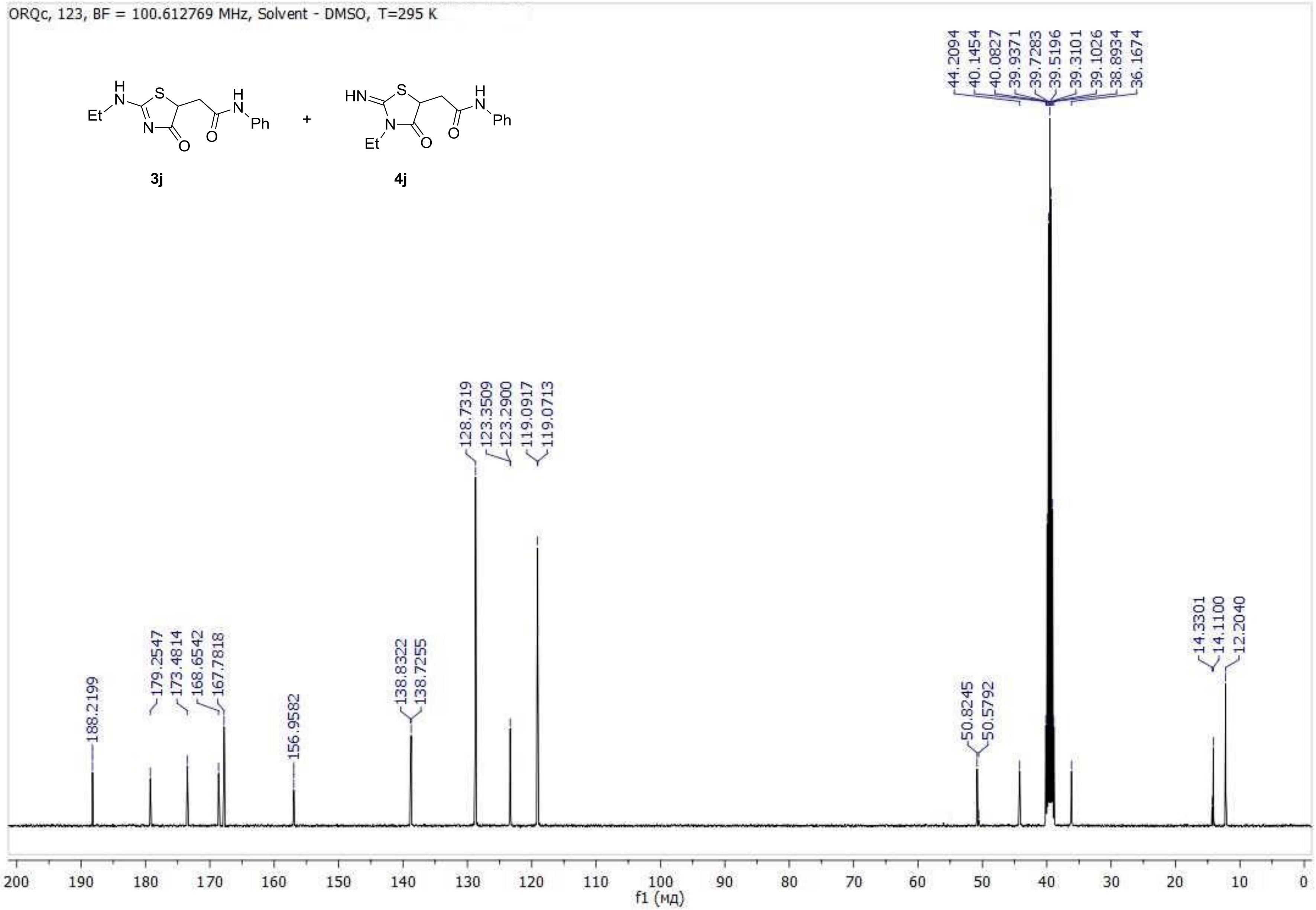
ORQc, 123, BF = 100.612769 MHz, Solvent - DMSO, T=295 K
3j
4j
188.2199
179.2547
173.4814
168.6542
167.7818
156.9582
138.8322
138.7255
128.7319
123.3509
123.2900
119.0917
119.0713
50.8245
50.5792
44.2094
40.1454
40.0827
39.9371
39.7283
39.5196
39.3101
39.1026
38.8934
36.1674
14.3301
14.1100
12.2040
f1 (мд)

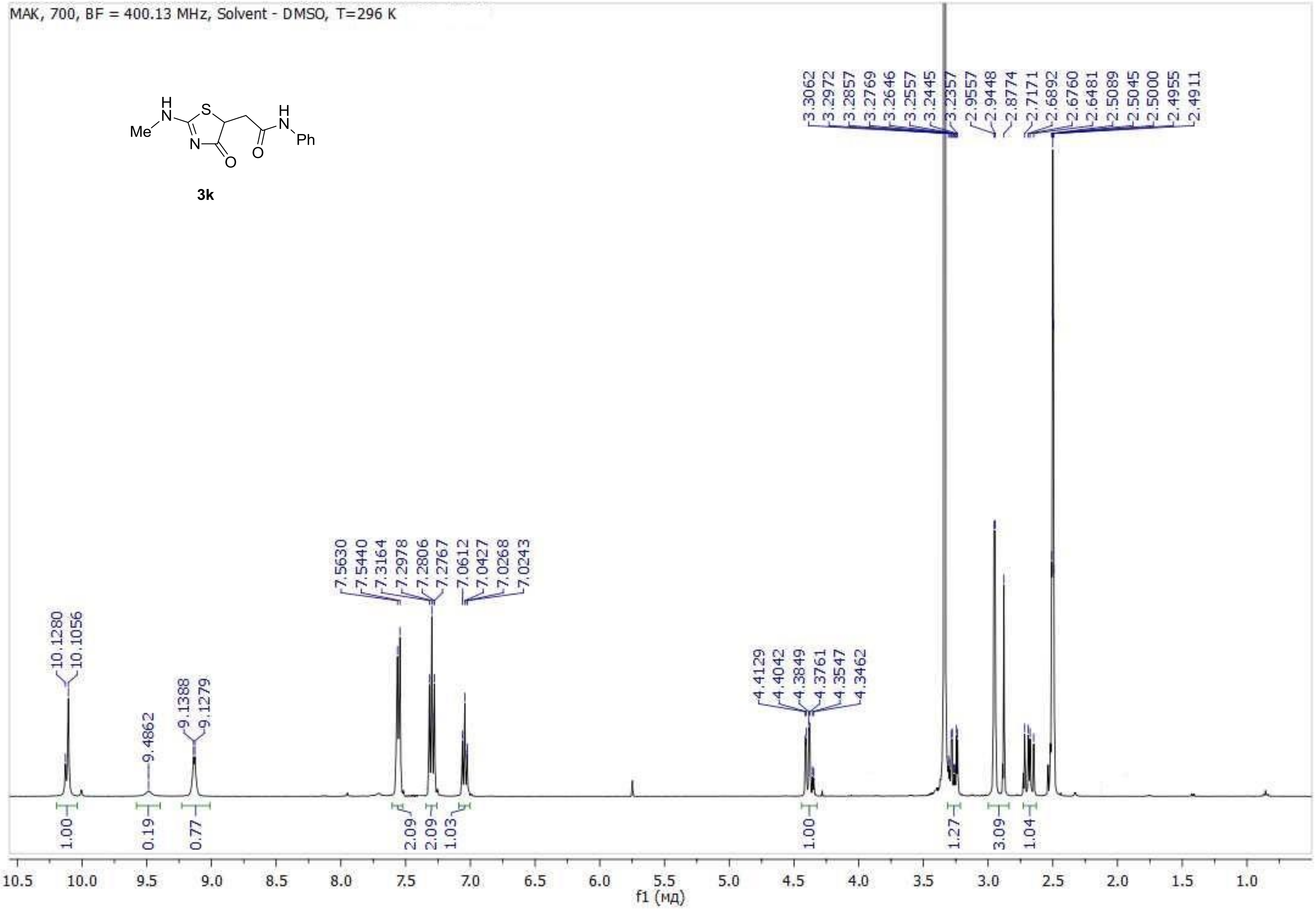
MAK, 700, BF = 400.13 MHz, Solvent - DMSO, T=296 K
3k
10.1280
10.1056
9.4862
9.1388
9.1279
7.5630
7.5440
7.3164
7.2978
7.2806
7.2767
7.0612
7.0427
7.0268
7.0243
4.4129
4.4042
4.3849
4.3761
4.3547
4.3462
3.3062
3.2972
3.2857
3.2769
3.2646
3.2557
3.2445
3.2357
2.9557
2.9448
2.8774
2.7171
2.6892
2.6760
2.6481
2.5089
2.5045
2.5000
2.4955
2.4911
1.00
0.19
0.77
2.09
2.09
1.03
1.00
1.27
3.09
1.04
f1 (мд)

ORQc, 312, BF = 100.612769 MHz, Solvent - DMSO, T=297 K

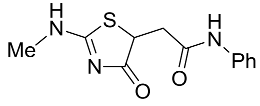

3k

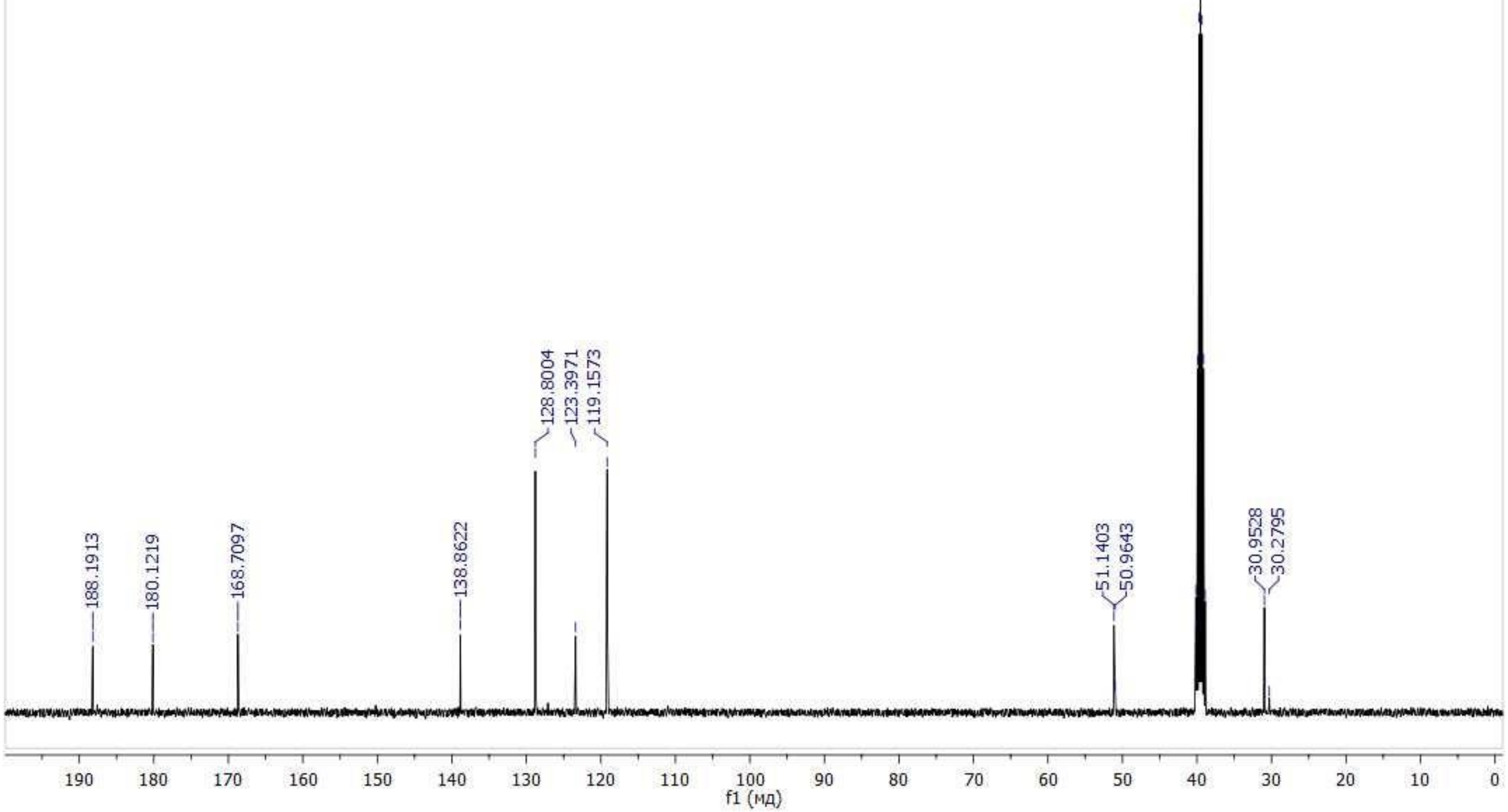

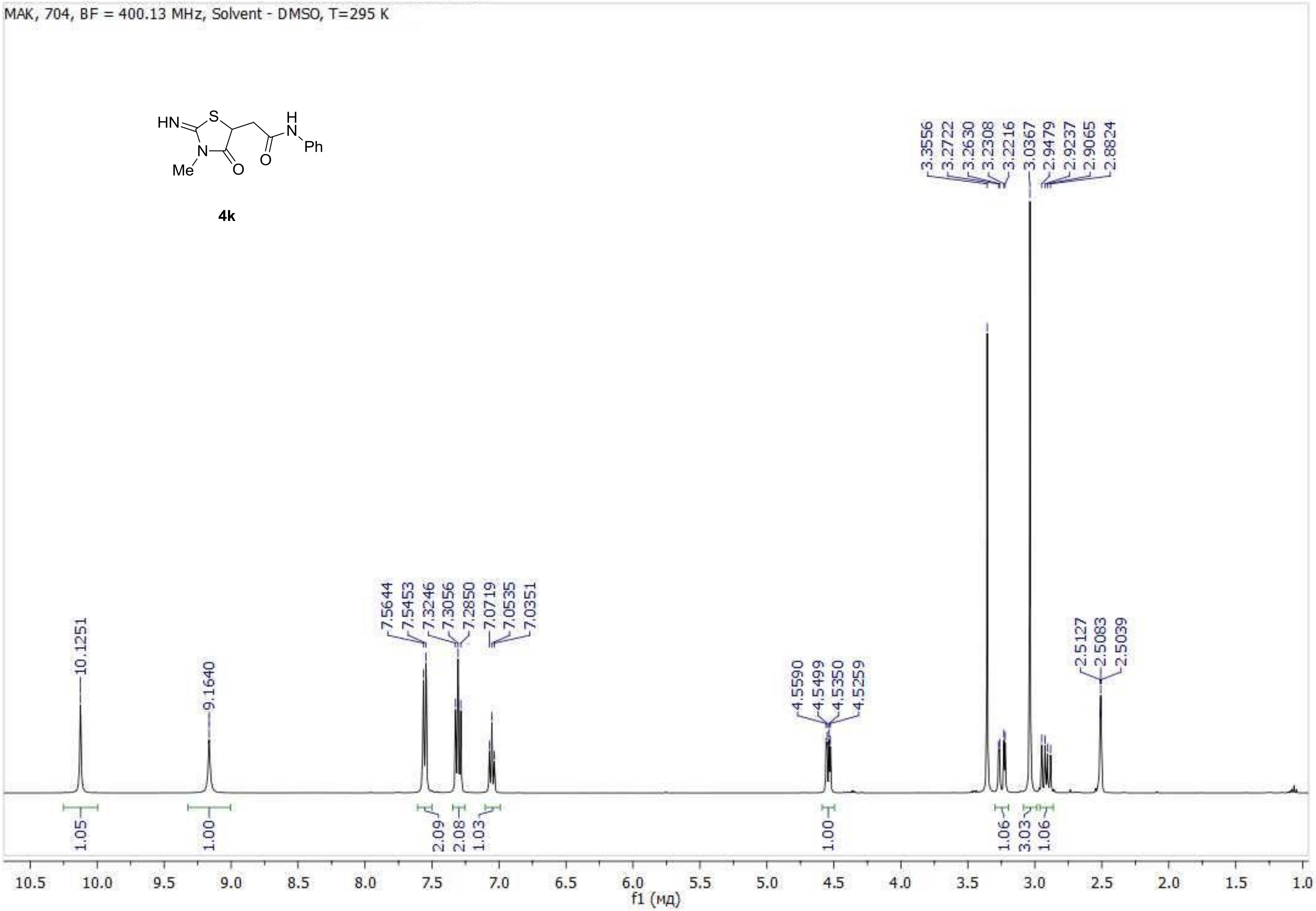
MAK, 704, BF = 400.13 MHz, Solvent - DMSO, T=295 K
HN
S
N
H
N
Ph
Me
O
O
4k
3.3556
3.2722
3.2630
3.2308
3.2216
3.0367
2.9479
2.9237
2.9065
2.8824
10.1251
9.1640
7.5644
7.5453
7.3246
7.3056
7.2850
7.0719
7.0535
7.0351
4.5590
4.5499
4.5350
4.5259
2.5127
2.5083
2.5039
1.05
1.00
2.09
2.08
1.03
1.00
1.06
3.03
1.06
f1 (мд)

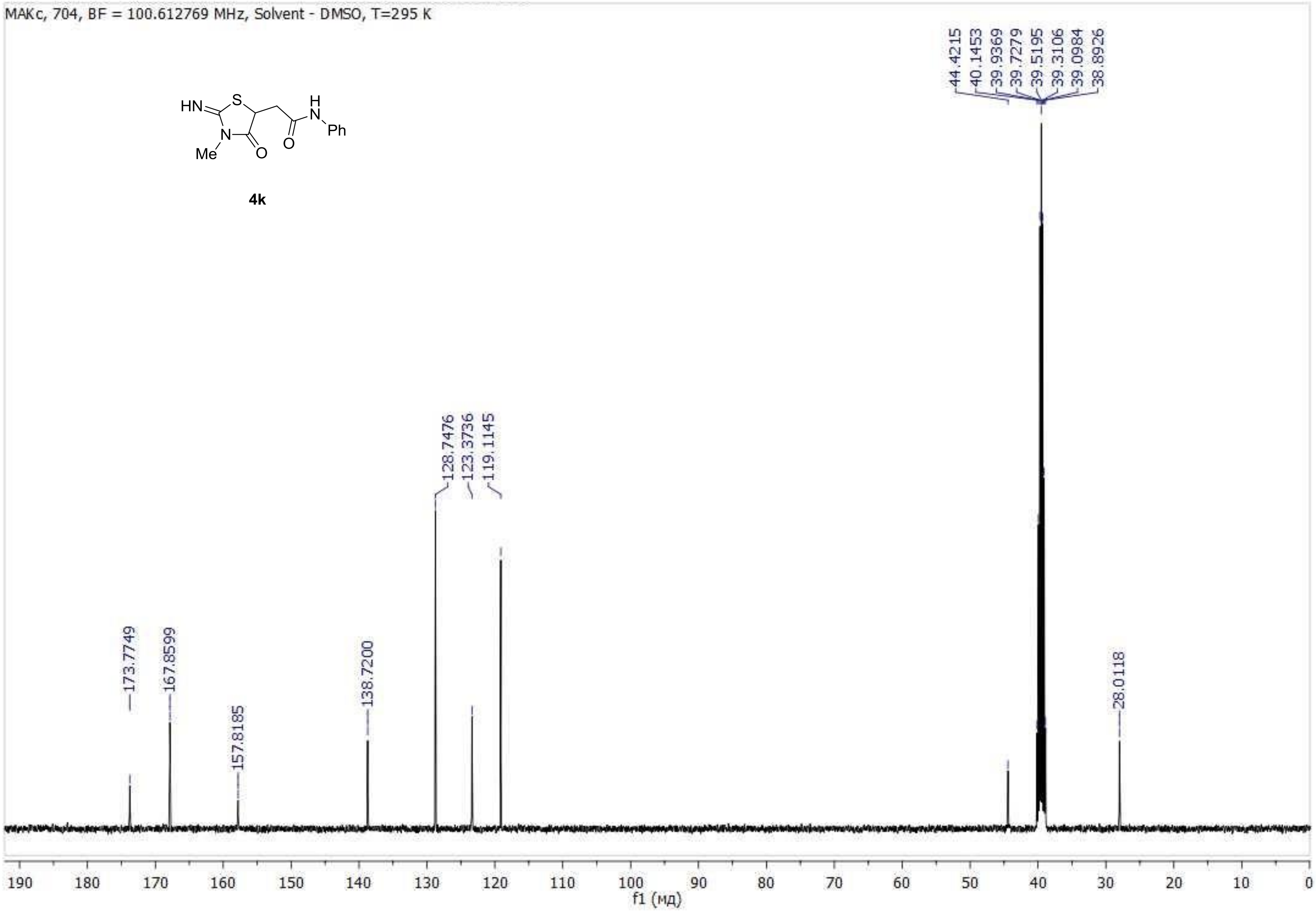
MAKc, 704, BF = 100.612769 MHz, Solvent - DMSO, T=295 K
HN S N Me O O H N Ph
4k
44.4215
40.1453
39.9369
39.7279
39.5195
39.3106
39.0984
38.8926
128.7476
123.3736
119.1145
173.7749
167.8599
157.8185
138.7200
28.0118
190 180 170 160 150 140 130 120 110 100 90 80 70 60 50 40 30 20 10 0
f1 (мд)

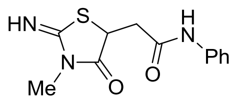

4k

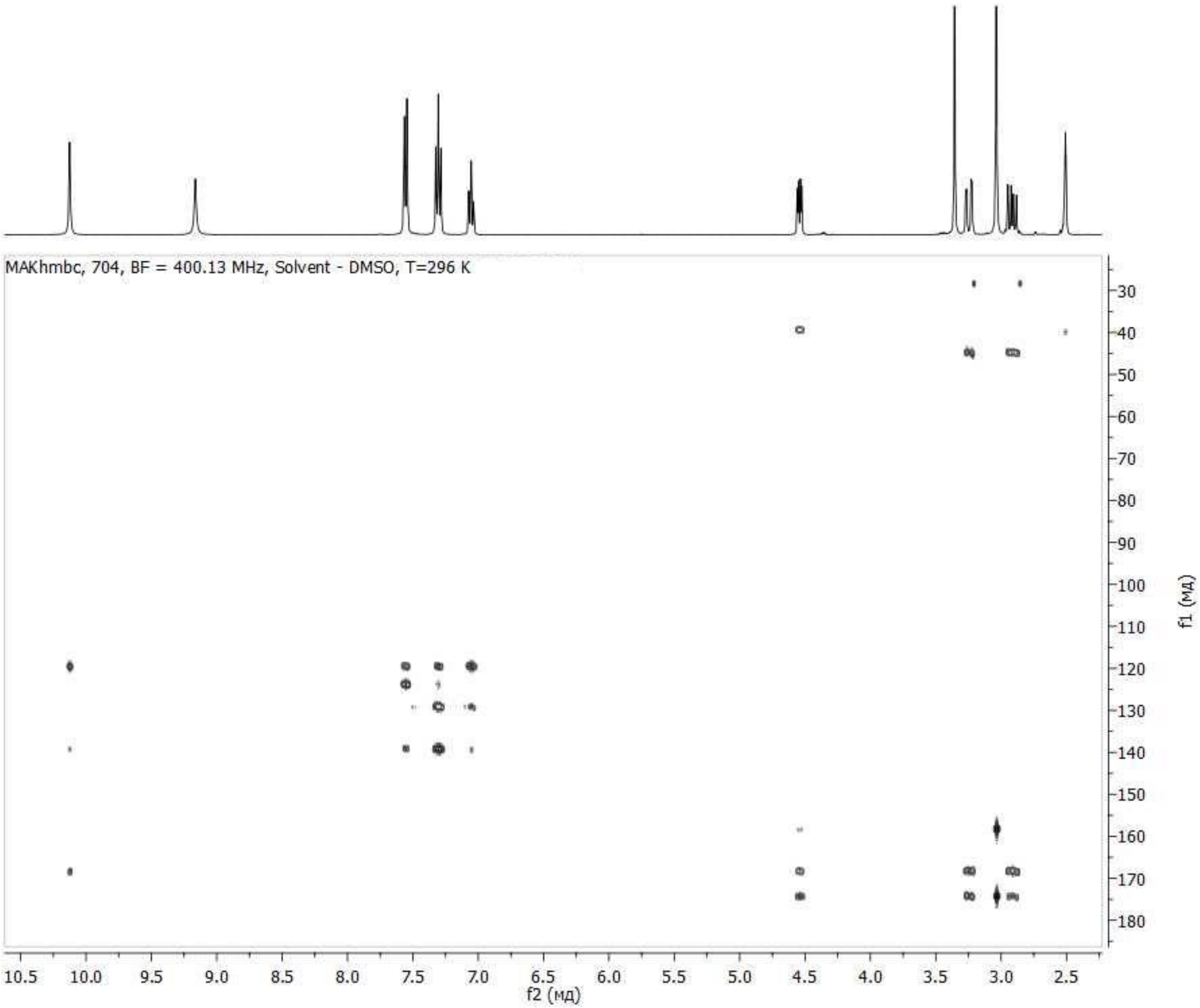

X-ray analysis data for thiazolidine 3b

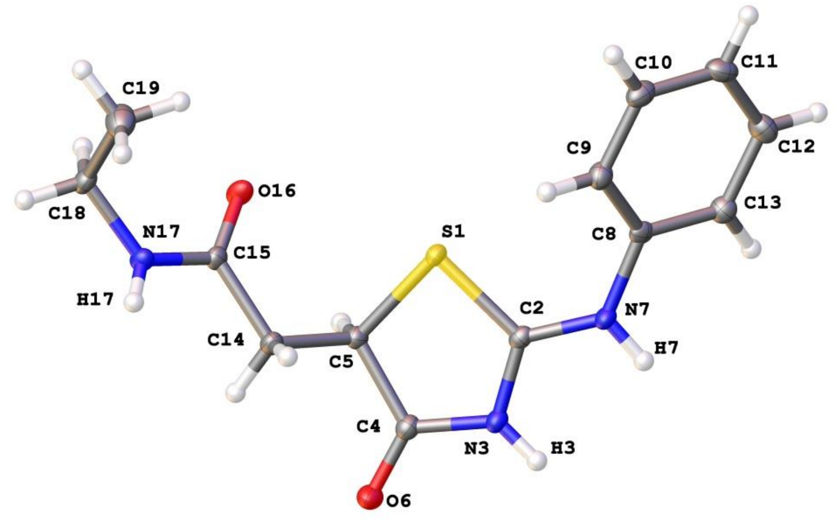

Figure S1: View to the $C_{13}H_{15}N_3O_2S$ molecule in the structure of **3b**. Carbon, nitrogen, oxygen and sulfur atoms are grey, light-blue, red and yellow, respectively. Thermal ellipsoids are drawn at the 50% probability level.

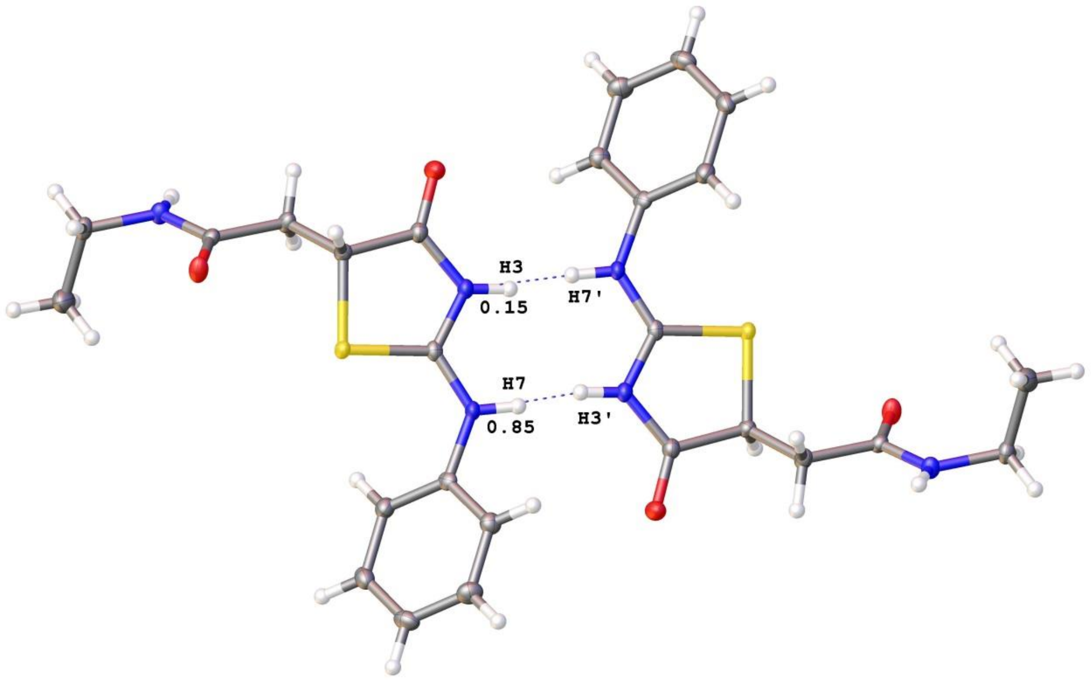

Figure S2: $(C_{13}H_{15}N_3O_2S)_2$ dimers formation via "jumping" hydrogen atom bonding between equivalent N3 and N7 atoms in the structure of **3b**.

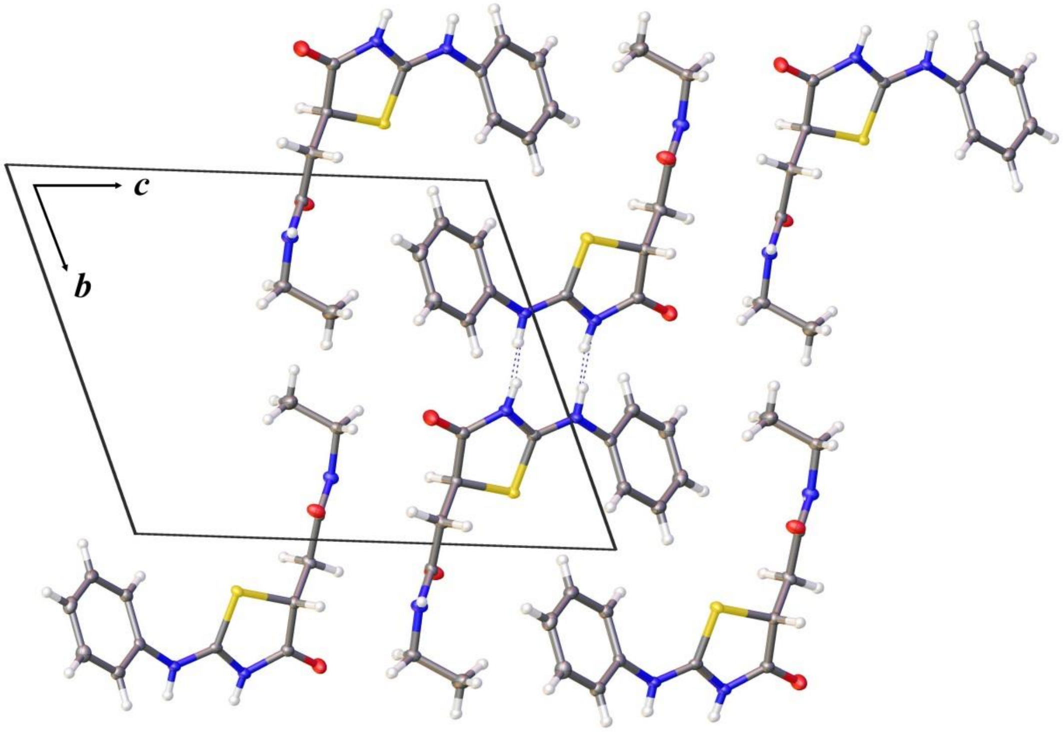

Figure S3: Crystal structure of **3b**, projection onto the (100) plane.

For the single crystal X-ray diffraction experiment, a crystal of **3b** was fixed on a micro mount, placed on a Agilent Technologies Supernova diffractometer equipped with an Atlas CCD detector and measured at a temperature of 100 K using micro-focused monochromated CuKα radiation. The unit cell parameters (Table S1) were refined by least square techniques using 11203 reflections in the 2θ range of 7.14–144.98°. The structure has been solved by the direct methods and refined R_1 = 0.031 (wR_2 = 0.077) for 2290 unique reflections with $|F_o| \geq 4\sigma_F$ by means of the SHELXL–97 program [2] incorporated in the *OLEX2* program package [3]. The carbon-bound H atoms were placed in calculated positions and were included in the refinement in the 'riding' model approximation, with U_{iso}(H) set to 1.5U_{eq}(C) and C–H 0.96 Å for CH_3 groups, with U_{iso}(H) set to 1.2U_{eq}(C) and C–H 0.97 Å for CH_2 groups, U_{iso}(H) set to 1.2U_{eq}(C) and C–H 0.93 Å for the CH groups. Nitrogen-bound hydrogen atoms were localized objectively with U_{iso}(H) set to 1.2U_{eq}(N). There are two partially occupied positions (0.15 and 0.85 a.p.f.u. for H3 and H7, respectively) of "jumping" hydrogen atom in the structure of **3b** with the total s.o.f. equal to 1.0. Empirical absorption correction was applied in CrysAlisPro [4] program complex using spherical harmonics, implemented in SCALE3 ABSPACK scaling algorithm.

Table S1: Crystallographic data for **3b**.

Compound	**3b**
Formula	$C_{13}H_{15}N_3O_2S$
Crystal System	Triclinic
a (Å)	4.8155(2)
b (Å)	10.8952(6)
c (Å)	13.2827(6)
α (°)	68.803(5)
β (°)	89.576(4)
γ (°)	82.881(4)
V (Å^3)	644.20(6)
Molecular weight	277.34
Space group	*P*-1
μ (mm^{-1})	2.259
Temperature (K)	100(2)
Z	2
D_{calc} (g/cm^3)	1.430
Crystal size (mm^3)	0.26×0.17×0.11
Radiation	Cu*K*α
Total reflections	11203
Unique reflections	2532
Angle range 2*θ* (°)	7.14–144.98
Reflections with $\|F_o\| \geq 4\sigma_F$	2290
R_{int}	0.0414
R_σ	0.0294
R_1 ($\|F_o\| \geq 4\sigma_F$)	0.0310
wR_2 ($\|F_o\| \geq 4\sigma_F$)	0.0770
R_1 (all data)	0.0357
wR_2 (all data)	0.0799
S	1.065
ρ_{min}, ρ_{max}, e/Å^3	–0.235, 0.352

$R_1 = \Sigma||F_o| - |F_c||/\Sigma|F_o|$; $wR_2 = \{\Sigma[w(F_o^2 - F_c^2)^2]/\Sigma[w(F_o^2)^2]\}^{1/2}$;
$w = 1/[\sigma^2(F_o^2)+(aP)^2 + bP]$, where $P = (F_o^2 + 2F_c^2)/3$; $s = \{\Sigma[w(F_o^2 - F_c^2)]/(n - p)\}^{1/2}$ where n is the number of reflections and p is the number of refinement parameters.

References

1. Marrian, D. H. *J. Chem. Soc.* **1949**, 1797–1799.
2. Sheldrick, G. M. *Acta Cryst.* **2008**, A64, 112.
3. Dolomanov, O. V.; Bourhis, L. J.; Gildea, R. J.; Howard, J. A. K.; Puschmann, H. *OLEX2: A complete structure solution, refinement and analysis program*, *J. Appl. Cryst.* **2009**, *42*, 339–341.
4. CrysAlisPro, Agilent Technologies, Version 1.171.36.32 (release 02-08-2013).
